# Diversity of electron-bifurcating CO_2_-fixing supercomplexes in methanogens

**DOI:** 10.1126/sciadv.aed3711

**Published:** 2026-09-16

**Authors:** Pablo San Segundo-Acosta, Shunsuke Nomura, Joao Pedro Fernandes-Queiroz, Evgenii Protasov, Jörg Kahnt, Masanori Kaneko, Georg Hochberg, Seigo Shima, Bonnie J. Murphy

**Affiliations:** ^1^ Redox and Metalloprotein Research Group, Max Planck Institute of Biophysics, Frankfurt am Main, Germany.; ^2^ Microbial Protein Structure Group, Max Planck Institute for Terrestrial Microbiology, Marburg, Germany.; ^3^ Geological Survey of Japan, National Institute of Advanced Industrial Science and Technology (AIST), Tokyo, Japan.; ^4^ Evolutionary Biochemistry Group, Max Planck Institute for Terrestrial Microbiology, Marburg, Germany.

## Abstract

In the hydrogenotrophic methanogenic pathway, formylmethanofuran dehydrogenase (Fmd) reduces and fixes CO_2_, driven by low-potential electrons provided by electron-bifurcating heterodisulfide reductase (Hdr) complexed with electron-donating proteins such as Mvh hydrogenase. Here, we report the structure of a C2-symmetric (Mvh-Hdr)_2_-Fmd_4_ supercomplex from a Class I methanogen, *Methanothermobacter marburgensis*, which is architecturally different from the previously reported ring-shaped D3-symmetric supercomplex of a methanogen belonging to phylogenetically distinct Class II methanogens. In this C2-symmetric form, the redox active sites of Hdr and Fmd are connected by two MvhB polyferredoxins, whose branching electron paths appear to be available for electron transfer to/from other partners. The ancestral form was likely C2 symmetric, whereas D3-symmetric supercomplexes were acquired by horizontal gene transfer, a transition probably helpful for growth in substrate-poor environments.

## INTRODUCTION

Methanogenic archaea are strictly anaerobic microorganisms that produce the potent greenhouse gas methane as a catabolic end product, contributing nearly three-quarters of the total annual global methane emissions (*1*, *2*). Most methanogens can produce methane by reduction of CO_2_ with H_2_, a metabolism known as hydrogenotrophic methanogenesis. Methanogens are highly diverse. Traditional classification included methanogens only in the superphylum Euryarchaeota, but recent studies show that members of other phyla like Thermoproteota (formerly TACK phylum) and Asgardarchaeota may be involved in methanogenesis, primarily by reduction of methylated compounds with H_2_ rather than through the reduction of CO_2_ (*3*–*6*). Among Euryarchaeota, methanogens are traditionally divided into two classes: Class I, containing Methanobacteriales, Methanococcales, and Methanopyrales; and Class II, containing Methanosarcinales, Methanocellales, and Methanomicrobiales (*7*).

In the first reaction of the hydrogenotrophic methanogenic pathway, CO_2_ is reduced and fixed as formylmethanofuran, a reaction catalyzed by molybdenum- or tungsten-based formylmethanofuran dehydrogenase (Fmd or Fwd, generalized here as Fmd) (*8*). The available electron donors, H_2_ and formate, are not sufficiently reducing to render CO_2_ fixation favorable; therefore, the low-potential electrons for the Fmd reaction are generated by flavin-based electron bifurcation (FBEB) in heterodisulfide reductase (Hdr) (*9*, *10*). FBEB is a form of bioenergetic coupling between two reduction reactions, allowing a more favorable reaction to provide driving force for a less favorable one (*11*, *12*). In Hdr, reduction of the high-potential electron acceptor heterodisulfide (*12*, *13*), which is produced as a by-product of methane release (*14*), is coupled to the energetically demanding reduction and fixation of CO_2_. Hdr forms a complex with an electron-donating module consisting of Hdr-associating [NiFe]-hydrogenase (Mvh), formate dehydrogenase (Fdh), or F_420_-dependent electron-donating proteins (Elp), which accept electrons from H_2_, formate, or the reduced form of F_420_, respectively (*9*, *15*–*17*).

In the standard scheme of methanogenic metabolism, ferredoxin is reduced by low-potential electrons from FBEB and diffuses through the cytosol to Fmd to drive CO_2_ fixation (*10*). However, copurification of Mvh/Fdh-Hdr and Fmd by pull-down assays has been reported in *Methanococcus maripaludis* (*16*). More recently, we reported the purification, characterization, and cryo–electron microscopy (cryo-EM) structures of a D3-symmetric hexameric Fdh-Hdr-Fmd complex from *Methanospirillum hungatei* (Fdh-Hdr-Fmd)_6_ (*15*), in which Fdh-Hdr is bound to Fmd. In that complex, low-potential electrons can be transferred directly from the bifurcating Hdr module to the CO_2_-reducing active site in Fmd without the need for diffusible ferredoxin, as confirmed by enzyme assays. This is accomplished by a long chain of [4Fe-4S]-clusters in the polyferredoxin subunit FmdF. Polyferredoxins are proteins with three to seven ferredoxin modules, each binding two [4Fe-4S] clusters, that are present in methanogenic and sulfate-reducing archaea and some bacteria (*18*). FmdF is one of several different polyferredoxins encoded in the genomes of diverse methanogenic species; additional polyferredoxin genes are found associated with gene clusters involved in central energy metabolism, including the *mvh* (Hdr-associated hydrogenase), *eha/ehb* (membrane-bound hydrogenase), and *rnf* (membrane-bound, sodium-translocating oxidoreductase) (*18*). The purified complex from *M. hungatei* was also able to reduce diffusible ferredoxin, but the site of ferredoxin interaction is unclear.

Recently, we purified a 1-MDa complex from *Methanothermobacter marburgensis* containing Mvh, Hdr, and Fmd. The complex catalyzes H_2_-dependent CO_2_ reduction via FBEB to form formylmethanofuran (*19*). Notably, in addition to FmdF, the 1-MDa fraction also contains polyferredoxin MvhB, which is only present in Class I methanogens (*18*). Here, we report the cryo-EM structure of this 1-MDa supercomplex, in which the electron-output domain of the HdrA subunits and electron-input domain of the FmdF subunits are connected via MvhB, forming a path of [4Fe-4S]-clusters for direct electron transfer (ET). In addition to this primary electron pathway, additional clusters within MvhB form an alternative path, which suggests a potential role for MvhB in receiving low-potential electrons from, or transferring them to, unknown partners in the cell. The architecture of the complex, which displays (Mvh-Hdr)_2_-Fmd_4_ stoichiometry and C2 symmetry, is different from that of the D3-symmetric (Fdh-Hdr-Fmd)_6_ complex from *M. hungatei* and dictates a different ratio of electron-bifurcating to CO_2_-fixing enzymes. However, the functional consequence of complexation is conserved: Low-potential electrons from the bifurcating HdrA subunits can be transferred to the multiple CO_2_-reducing active sites of Fmd. Phylogenetic and ancestral sequence reconstruction analyses suggest that the architecture of the complexes evolved divergently to efficiently transfer low-potential electrons from Hdr to Fmd under distinct environmental conditions.

## RESULTS

### Architecture of the Mvh-Hdr-Fmd complex from *M. marburgensis*

**Fig. 1.**
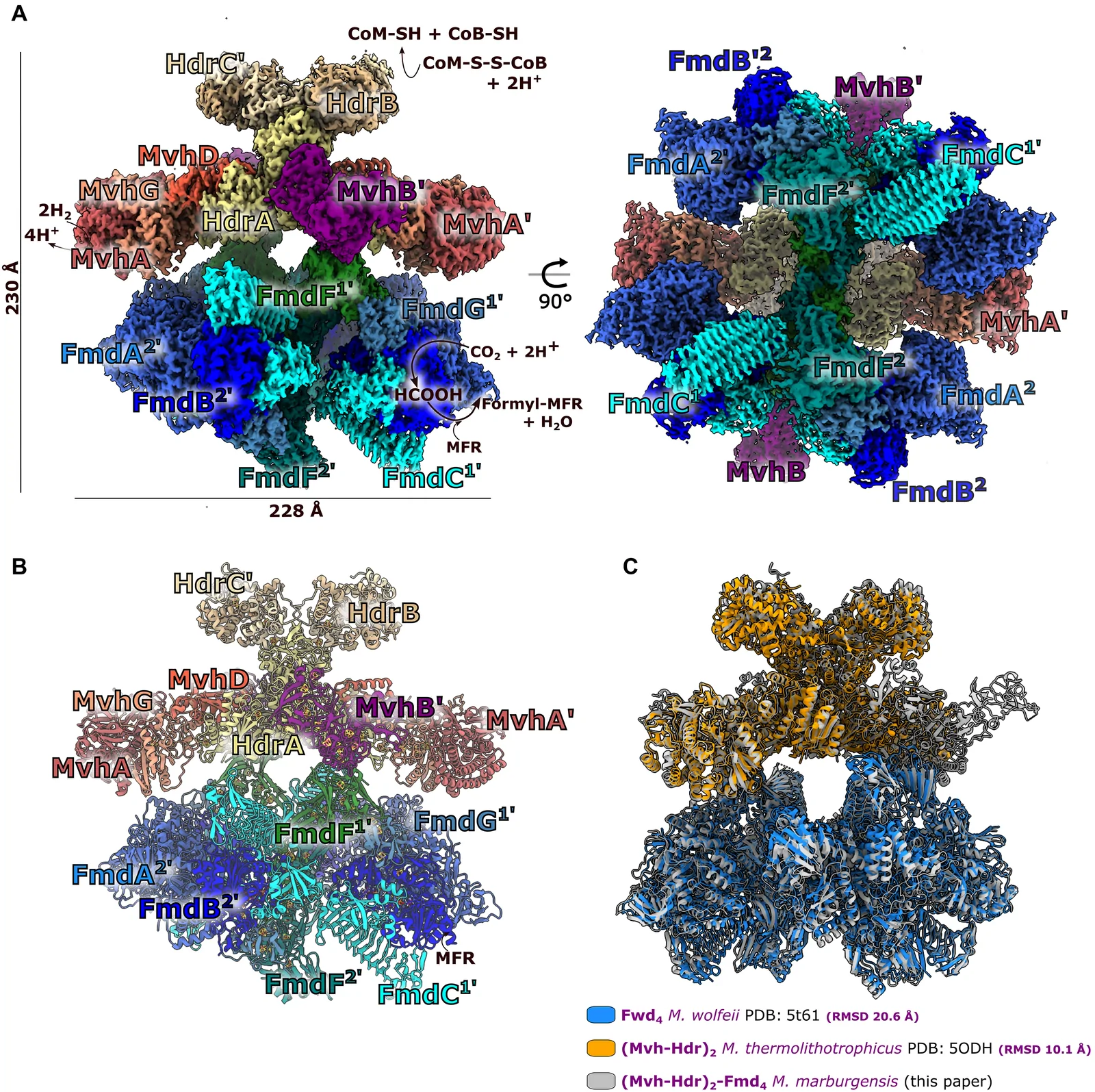
*M. marburgensis* Mvh, Hdr, and Fmd enzymes form a C2-symmetric supercomplex with (Mvh-Hdr)_2_-Fmd_4_ stoichiometry. (**A**) Side and bottom views of the composite cryo-EM map of the Mvh-Hdr-Fmd supercomplex colored by subunit. The same coloring is used throughout the paper unless otherwise stated. (**B**) Side view of the atomic model of the Mvh-Hdr-Fmd supercomplex in cartoon representation. (**C**) Crystal structures of the Fwd tetramer from *M. wolfeii* (PDB: 5T61; blue) (*8*) and the Mvh-Hdr dimer from *M. thermolithotrophicus* (PDB: 5ODH; orange) (*9*) are superimposable to the Mvh-Hdr-Fmd structure (gray), which indicates the high structural similarity.

**Fig. 2.**
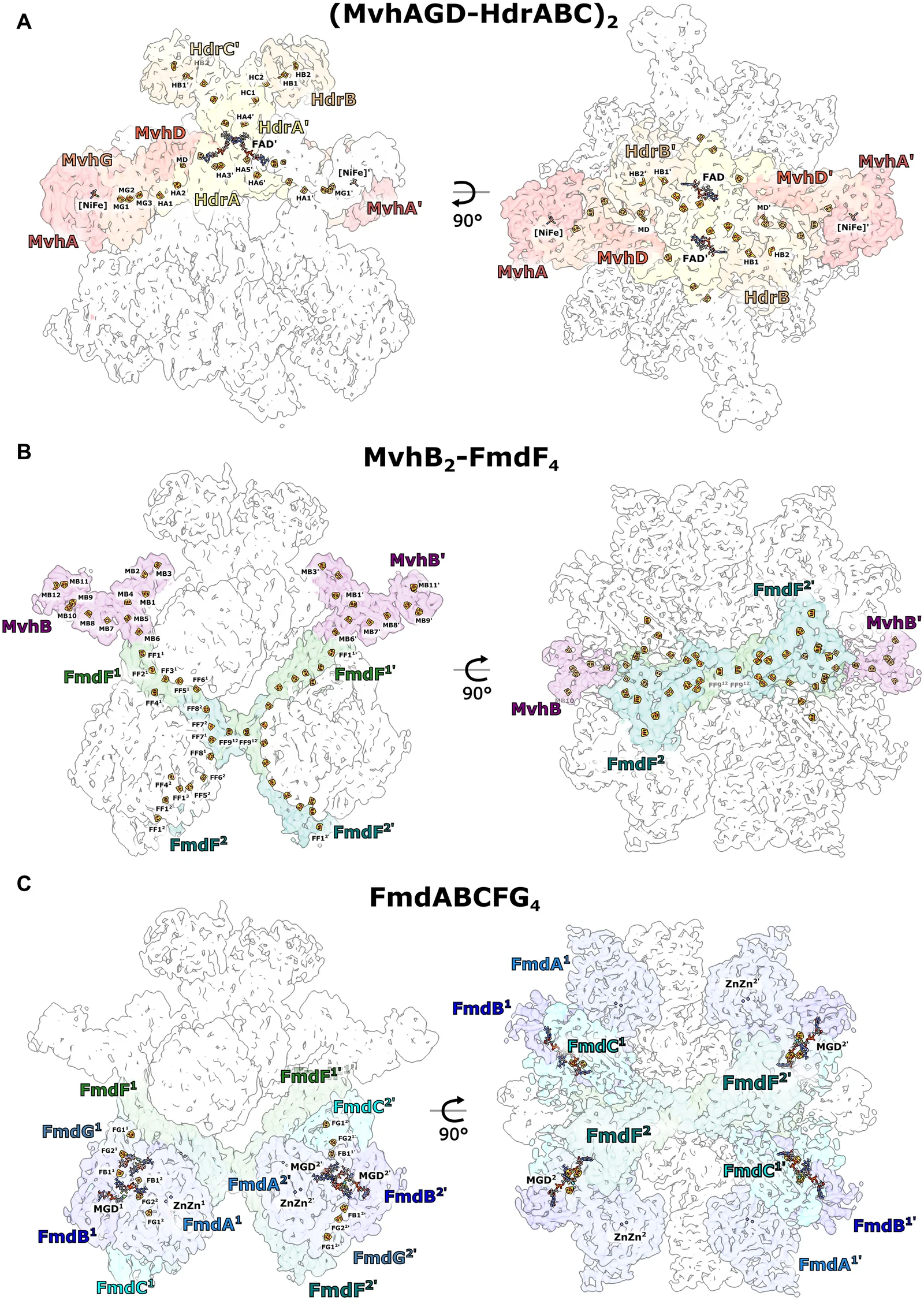
Side and bottom views of the composite map of the (Mvh-Hdr)_2_-Fmd_4_ supercomplex. The individual subcomplexes are depicted in colors to highlight the electron transfer pathway via the iron-sulfur clusters and FAD. (**A**) Hydrogenase–heterodisulfide reductase subcomplex, (Mvh-Hdr)_2_. (**B**) Polyferredoxin unit, MvhB_2_-FmdF_4_. (**C**) CO_2_-fixing tetrameric Fmd subcomplex, (FmdABCFG)_4_. Iron-sulfur clusters, FAD, and molybdopterin (MGD) are depicted as stick models. In molybdenum-based Fmd, subunits C and D are often fused. Dinuclear zinc centers (ZnZn) are shown as gray balls. The cofactors of the different subunits are shown as stick or ball models and named following the conventions established in previous studies (*8*, *9*, *15*).

**Fig. 3.**
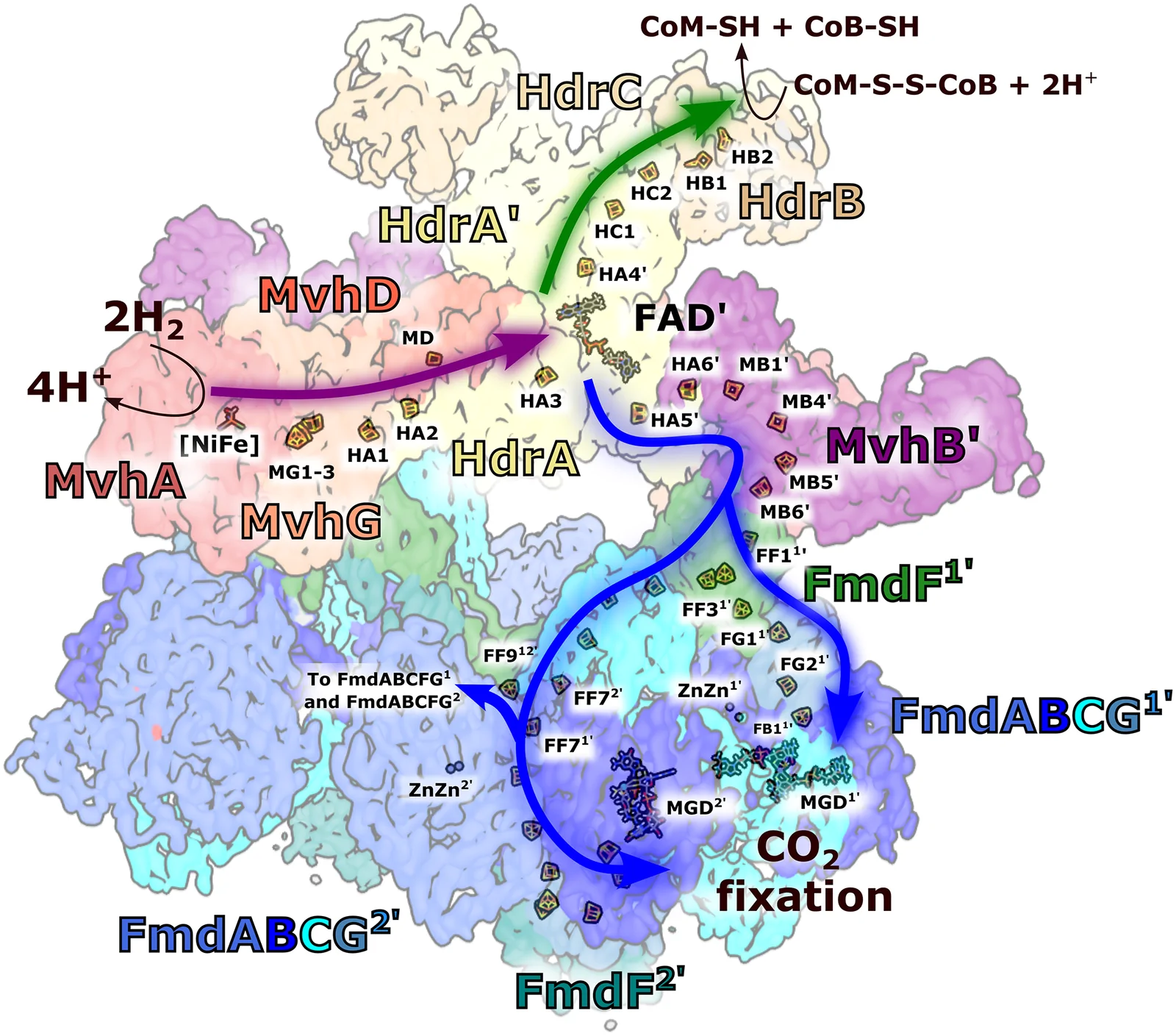
Schematic representation of the CO_2_-reducing electron transfer pathways in the (Mvh-Hdr)_2_-Fmd_4_ supercomplex. Electrons originating from H_2_ oxidation by the Mvh hydrogenase (purple arrow) are transferred to the bifurcating FAD in the Hdr module. Following FBEB, a high-potential electron is directed toward heterodisulfide reduction (green arrow), whereas low-potential electrons are transferred through polyferredoxins MvhB and FmdF to the CO_2_-reducing Fmd complex (blue arrows). More details into the FBEB mechanism and distances between cofactors are shown in Fig. 4. Abbreviations are defined in the legend of Fig. 2.

We purified the Mvh-Hdr-Fmd complex from the cell extract of *M. marburgensis* cultivated under a H_2_ and CO_2_ atmosphere and in standard nickel-sufficient medium containing molybdenum, as previously described (*19*). Under these conditions, Mvh is produced as an electron-donating module of Hdr and both isoenzymes of Fmd form an enzyme complex with Mvh-Hdr of ∼1 MDa (see Materials and Methods and fig. S1) (*17*). The sample was anaerobically prepared for cryo-EM analysis (figs. S2 to S22 and tables S1 to S4). Preliminary data processing revealed two-dimensional (2D) classes and an ab initio model of a C2-symmetric supercomplex (fig. S2A). Three-dimensional (3D) refinements gave a map of the whole complex showing clear densities for the HdrABC and Fmd units (fig. S2B) but with smeared densities for the N- and C-terminal domains of HdrA in complex with MvhA, MvhG, and MvhD (here referred to as the “mobile arm”). Using 3D classifications followed by masked focused refinements, we resolved high-resolution maps of all parts of the complex (fig. S3). These focused maps were combined to give the C2-symmetric composite map (Fig. 1A and fig. S4) with (Mvh-Hdr)_2_-Fmd_4_ stoichiometry (Fig. 1B and figs. S23 and S24). The complex is architecturally different from the previously reported *M. hungatei* (Fdh-Hdr-Fmd)_6_ complex (fig. S23). In *M. marburgensis*, the Fmd subcomplexes form an “hourglass” tetramer highly similar to that observed in the crystal structure of the *Methanothermobacter wolfeii* enzyme (Figs. 1C and 2, B and C) (*8*), and the Mvh-Hdr closely resembles the crystal structure of the Mvh-Hdr dimer from *Methanothermococcus thermolithotrophicus* (Figs. 1C and 2A) (*9*). A schematic overview of the electron transfer pathways linking H_2_ oxidation to CO_2_ reduction in the (Mvh-Hdr)_2_-Fmd_4_ supercomplex is presented in Fig. 3. The (Mvh-Hdr)_2_-Fmd_4_ structure exhibits a nonstoichiometric interaction of the Hdr and Fmd modules, such that only two of the four FmdF subunits interact with HdrA via MvhB, leaving two potential interaction sites of FmdF unfilled. No additional density indicating partial occupancy of bound partners was observed at these sites (figs. S2 and S3). This poses the question of why interaction of a further Hdr/MvhB dimer is not seen at this site. A possible explanation is that this interaction is transient and therefore not observed under the purification and/or imaging conditions. It could also be due to the fact that the Fmd tetramer is flexed somewhat, breaking symmetry around one axis (fig. S24, A and B) and posing steric constraints on the interaction of an additional Hdr dimer to form a fully tetrameric Mvh-Hdr-Fmd complex (fig. S24, C and D). This finding suggests that the specific interactions of FmdF (FwdF) within the tetrameric structure enforce a nonstoichiometric interaction between electron-bifurcating and CO_2_-reducing subcomplexes. We note that the purified and crystallized FwdABCDFG tetramer from *M. wolfeii* also shows this symmetry breaking, even though that complex was crystallized in the absence of an interacting Hdr dimer (fig. S24B).

### Conserved Cys residues of HdrA bind an unknown ligand

Two well-defined conformational states of the mobile arm have been previously characterized for the *M. hungatei* (Fdh-Hdr-Fmd)_6_ complex (*15*) and the *M. marburgensis* Elp-Hdr complex (*17*). Although these states provide important insight into the conformational gating of electron transfer in the complex, a critical gap exists in our understanding of the mechanism of electron transfer from the [2Fe-2S] cluster MD of MvhD to the bifurcating flavin of HdrA. This is complicated by the fact that the high-energy flavosemiquinone state invoked in the proposed mechanism of FBEB (*12*) seems to be incompatible with flavin reduction in two one-electron steps, as should be the case for electron transfer steps mediated by a chain of Fe-S clusters. Conserved additional cysteine residues (Cys^202^ and Cys^205^ in HdrA and Cys^16^ in MvhD) (*9*) that lie between the [2Fe-2S] cluster and FAD in conformational state 1 (*15*) may play a role in this electron transfer.

**Fig. 4.**
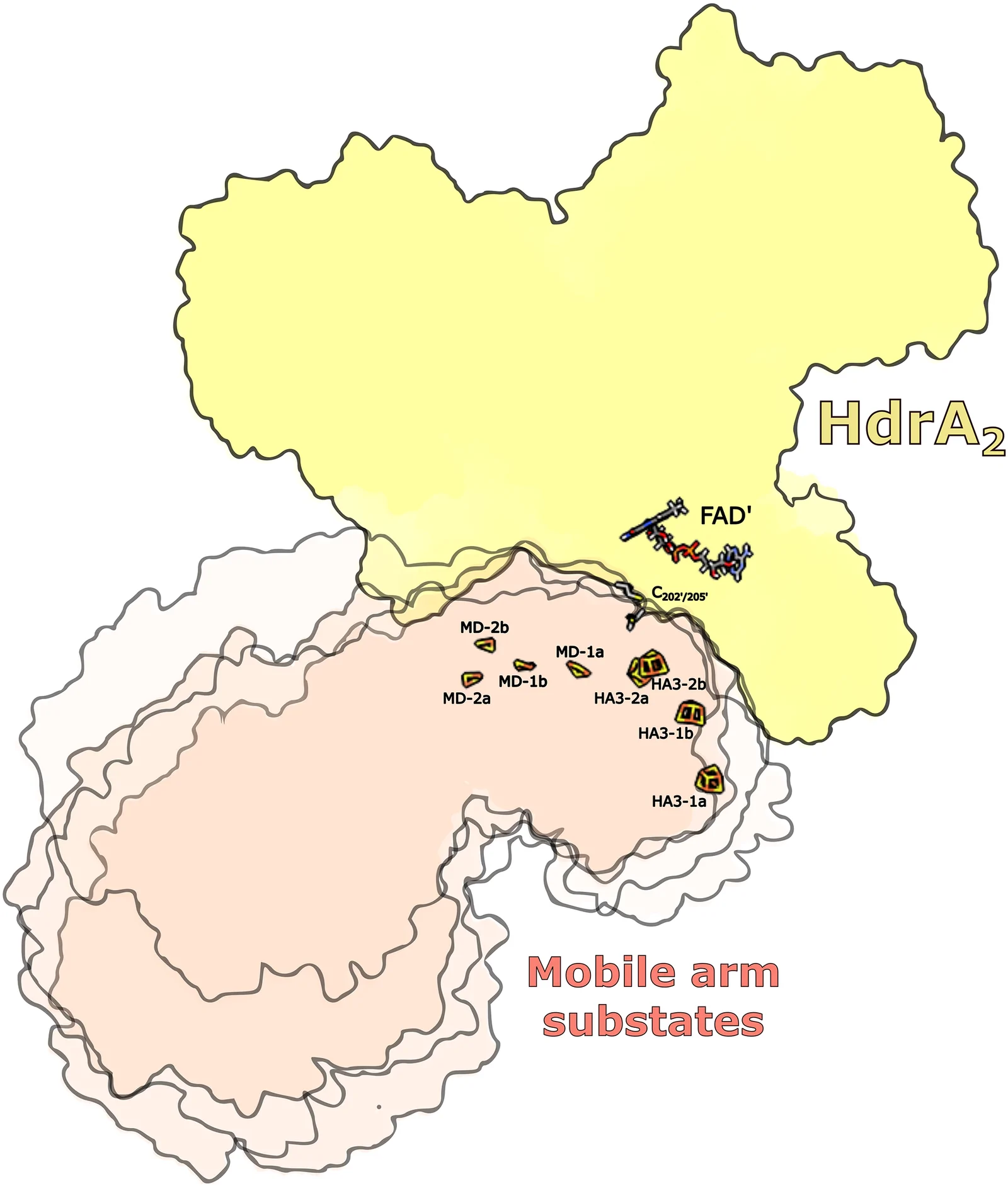
Conformational states of the Mvh-Hdr mobile arm obtained by symmetry expansion, focused 3D classification, and local refinement. The [2Fe-2S] cluster of MvhD (MD), [4Fe-4S] “shuttle” cluster (HA3), conserved cysteine residues Cys^202^/Cys^205^ and bifurcating flavin (FAD) groups are shown for states 1a, 1b, 2a, and 2b.

**Fig. 5.**
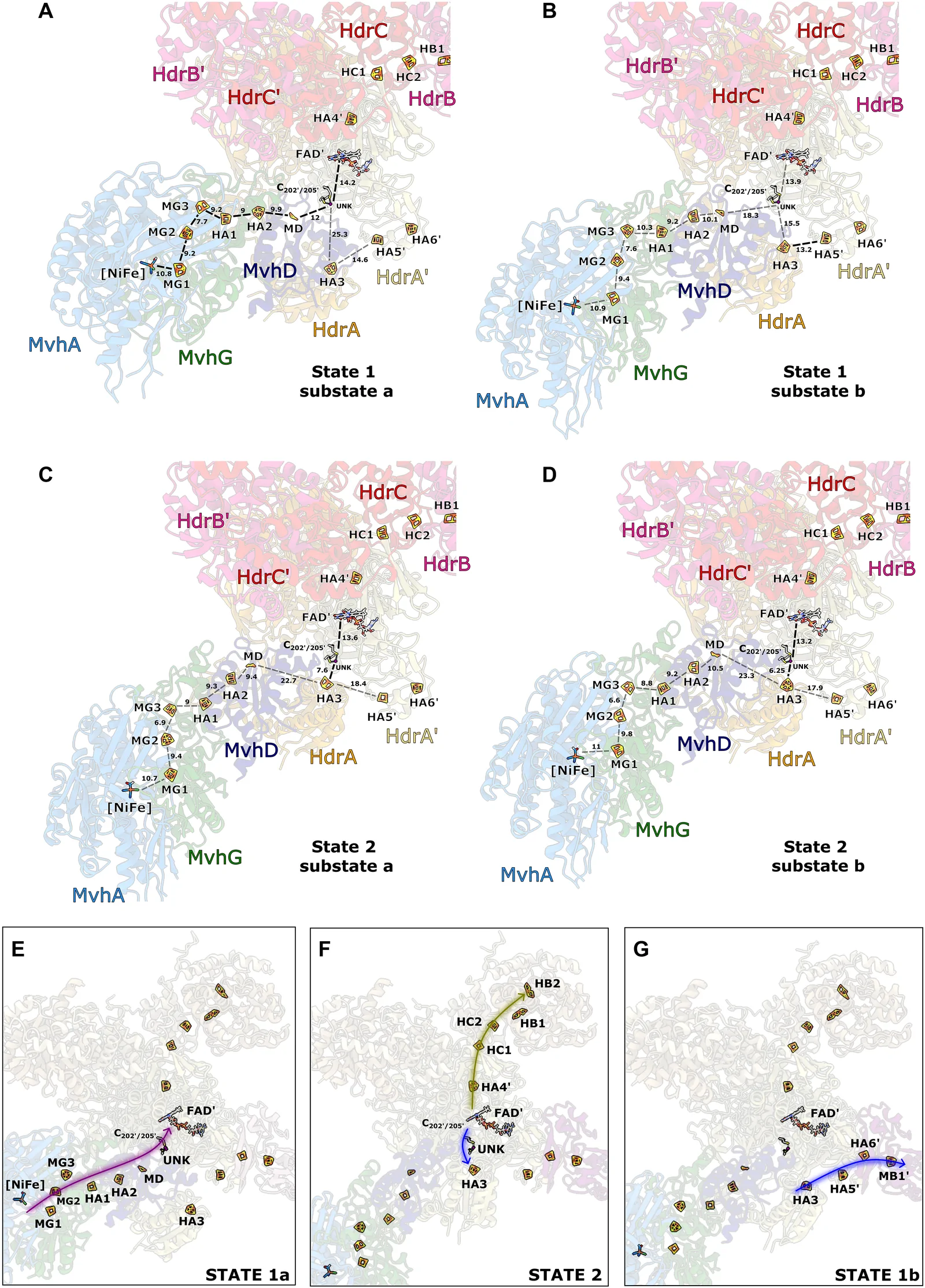
Conformational states of the Mvh-Hdr mobile arm and proposed FBEB reaction. These conformations (**A** to **D**) were obtained by symmetry expansion, 3D classification, and local refinement. Distances between cofactors are indicated with dashed lines and measured in Å. (**E**) In state 1a, MD approaches Cys^202′^ and Cys^205′^ of HdrA and the bifurcating FAD′ of HdrA′, which is thought to allow electron transfer by an unknown mechanism. The electron transfer possibly involves Cys^202′^ and Cys^205′^ of HdrA and the coordinated unknown (UNK) ligand (purple arrow). (**F**) Rotation of the mobile arm brings the complex into state 2a or 2b. A high-potential electron is transferred from the fully reduced FAD′ to the noncubane [4Fe-4S] clusters of HdrB (HB1 and HB2) via cluster HA4′ for reduction of CoM-S-S-CoB (green arrow). The low-potential electron is transferred from the FAD′ semiquinone to the isolated cluster HA3, which acts as an electron shuttle (blue arrow). (**G**) Conformational change back to state 1b brings HA3 back within electron transfer distance of cluster HA5′ of HdrA′, thereby forming a conductive pathway to the CO_2_-reducing site of Fmd via MvhB MB1′ (blue arrow). Subunits MvhA, D, and G and HdrB and C are colored differently from Fig. 1 to facilitate visualization. Abbreviations are defined in the legend of Fig. 2.

**Fig. 6.**
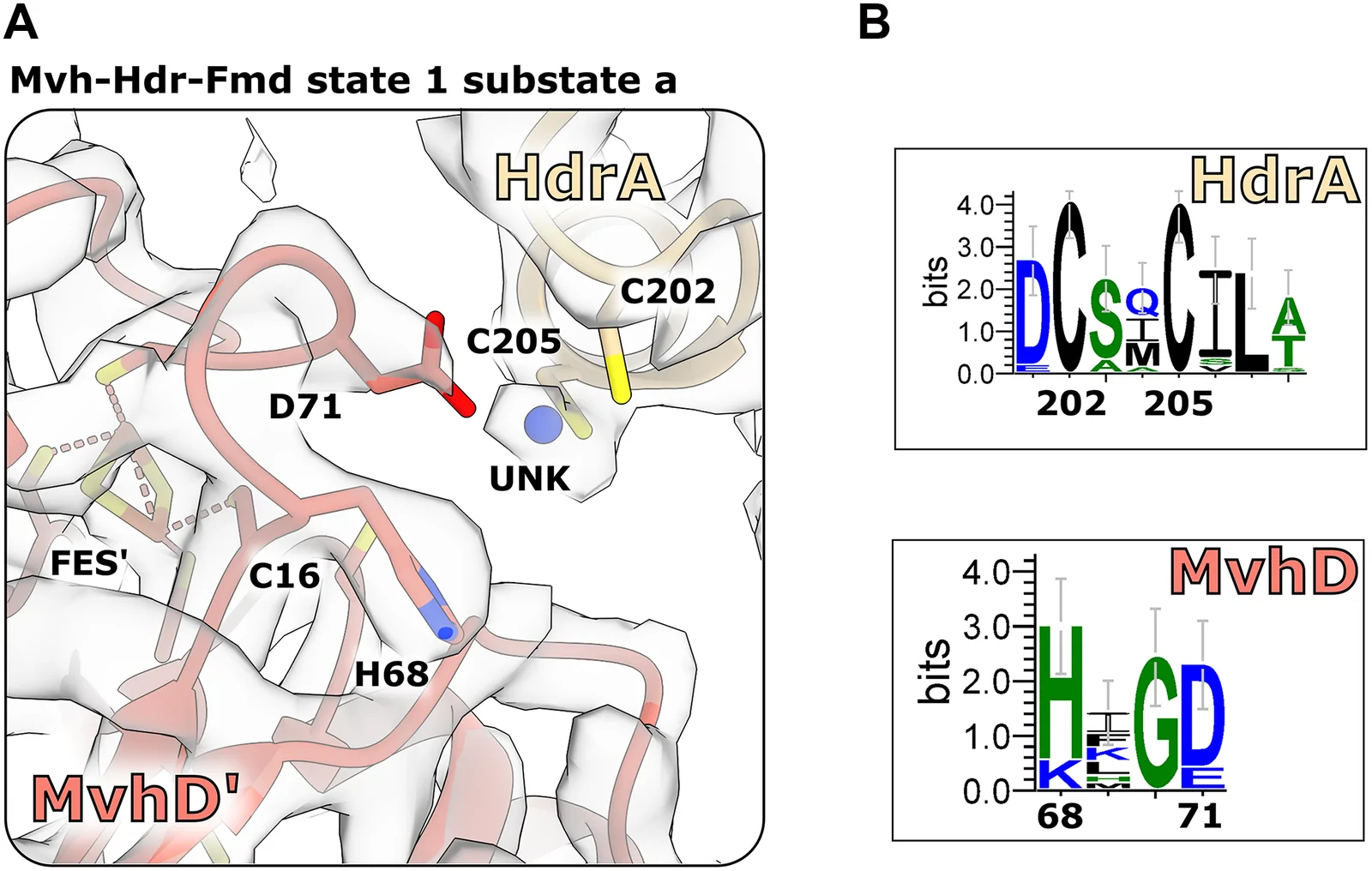
An unknown ligand (UNK) bound to the conserved cysteines of HdrA in the (Mvh-Hdr)_2_-Fmd_4_ supercomplex. (**A**) In conformational state 1a, the ligand appears also to be bound by conserved Asp^71^ of MvhD. His^68^ is near but does not coordinate. (**B**) Sequence conservation across the selected residues (*M. marburgensis* HdrA Cys^202^/Cys^205^ and MvhD His^68^/Asp^71^). Further information on the alignments is found in the Materials and Methods section. A similar UNK ligand was observed in other conformational states in this complex and in the Elp-Hdr complex from *M. marburgensis* (fig. S25) (*17*). Abbreviations are defined in the legend of Fig. 2.

Focused 3D classification revealed four distinct substates of the mobile arm (Figs. 4 and 5 and figs. S2, S5, and S6). Two of these resemble mobile arm state 1 previously described for the *M. hungatei* Fdh-Hdr-Fmd complex (*15*) and the *M. marburgensis* Elp-Hdr complex (*17*) (here termed substates 1a and 1b), whereas the other two are similar to state 2 (substates 2a and 2b). All reconstructions show a strong, well-defined density coordinated by the conserved HdrA cysteine pair Cys^202^/Cys^205^ (Fig. 6A and fig. S25A). In substate 1a, this density additionally appears to be coordinated by MvhD Asp^71^ and is near His^68^ (Fig. 6A). These residues are highly conserved among Euryarchaeota methanogens, with only conservative substitutions observed in some species (His to Lys and Asp to Glu; Fig. 6B and fig. S26). Comparable densities are also present in the Elp-Hdr structures (EMD-19533 and EMD-19538) (*17*), where coordination by His^67^ and Asp^70^ in ElpC, an MvhD homolog, appears likely in state 1 (fig. S25B). The identity and specific role of this ligand are unclear; nevertheless, our observation further strengthens the idea, based on sequence conservation (Fig. 6B) (*9*), that the cysteine pair Cys^202^/Cys^205^ is dynamic and could play a functional role in the mechanism of FBEB, specifically in mediating electron transfer from the MvhD [2Fe-2S] cluster to the bifurcating flavin group. Our observations widen the scope of mechanistic possibilities to include also bound cofactors and, in conformational state 1, conserved acidic (Asp^71^) and basic (His^68^) residues.

The proposed electron transfer pathway involving FBEB is depicted in Fig. 5 (E to G). In state 1a, electrons from H_2_ travel via MvhG to the MvhD [2Fe-2S] cluster MD and from there are predicted to be transferred to the bifurcating FAD′ of HdrA′ (Fig. 5E). Although the distance between MD and FAD′ of HdrA′ (22 Å) is too far for rapid, direct electron transfer, the position of the conserved Cys^202′^/Cys^205′^ pair between these groups, and the coordinated ligand, suggest that these may play a role in facilitating the electron transfer, although further studies will be needed to clarify the mechanism. When the complex transitions to state 2 (Fig. 5F)—either substate 2a or 2b, as both are very similar and could be functionally equivalent (Fig. 5, C and D)—the fully reduced FAD′ can transfer a weakly reducing electron via HA4′ to the HdrB active site (HB1 and HB2). Then, a strongly reducing electron from the FAD^•−^′ semiquinone can be transferred to HA3, the “shuttle cluster.” Last, when the complex transitions to state 1b, this electron can be transferred to HA5′ and onward via HA6′ to MvhB MB1′.

### Polyferredoxin MvhB and proposed electron pathways of the Mvh-Hdr-Fmd complex

The *mvh* operon of Class I methanogens contains the genes encoding the Mvh hydrogenase subunits (MvhAGD) and polyferredoxin MvhB in the *mvhDGAB* gene cluster (fig. S27). MvhB had been previously characterized as a soluble protein with high affinity for Mvh-Hdr and Fmd, potentially acting as a diffusible low-potential electron carrier (*18*, *20*). However, we recently described the structure of the *M. marburgensis* Elp-Hdr complex, in which the N-terminal domain of MvhB could be observed bound to HdrA in a subset of particles (*17*). Nevertheless, Fmd subunits in the Elp-Hdr could not be resolved, most probably due to complex instability (*19*).

**Fig. 7.**
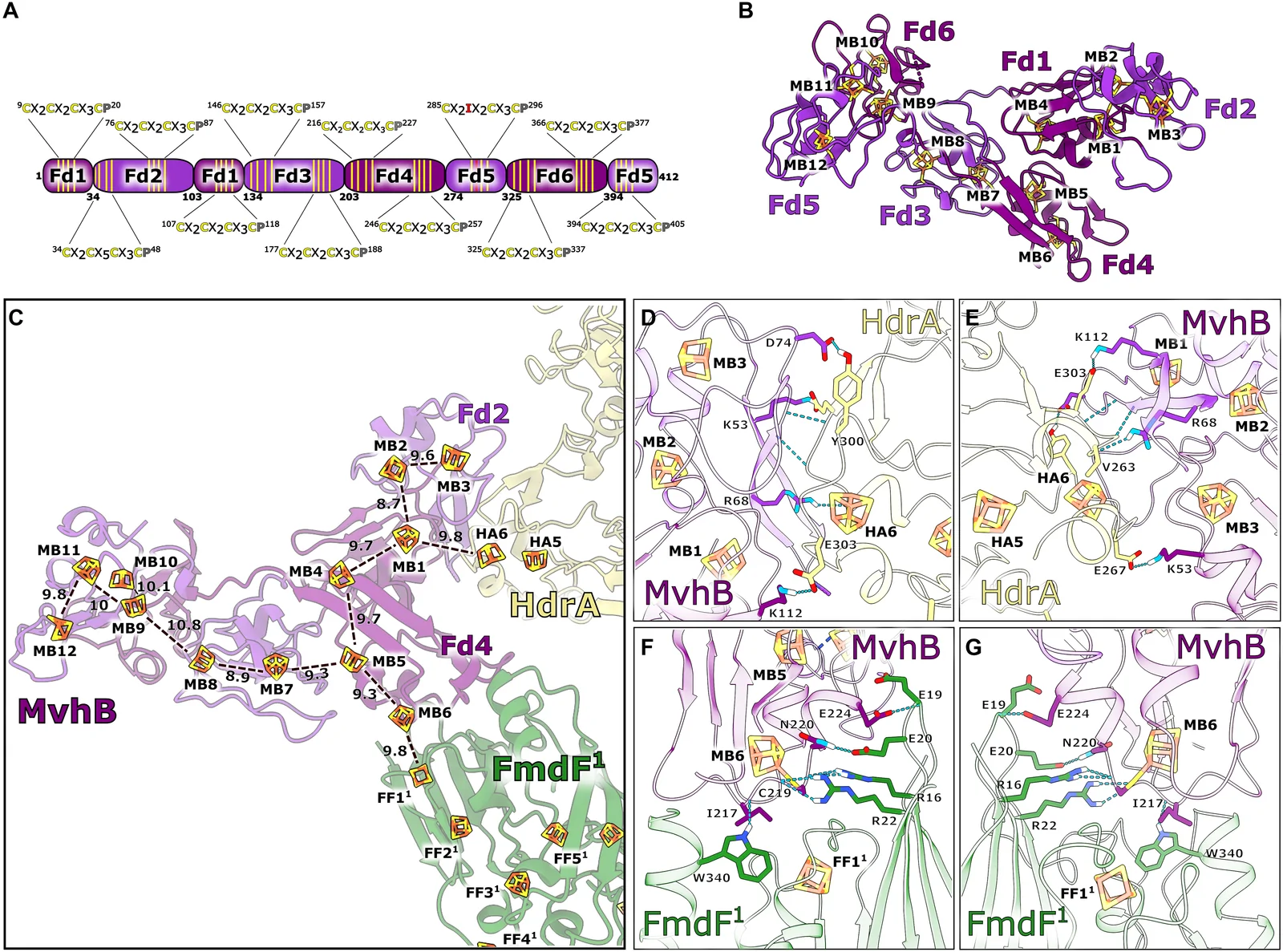
Polyferredoxin MvhB connects HdrA and FmdF electronically in the (Mvh-Hdr)_2_-Fmd_4_ supercomplex. (**A**) Schematic view of the six ferredoxin domains (Fd1 to Fd6) of MvhB, indicating the position of the iron-sulfur-cluster-binding motifs. (**B**) Atomic model of MvhB, showing the positions of the 11 [4Fe-4S] clusters (MB1 to MB11) and the [3Fe-4S] cluster (MB12). (**C**) MvhB interacts with HdrA through Fd2 and with FmdF via Fd4. Distances between clusters (shown in Å) suggest that HA6 transfers electrons to MvhB via MB1, whereas MB6 transfers electrons to FmdF via FF1′. (**D** and **E**) Detailed view of the MvhB and HdrA interface. (**F** and **G**) Detailed view of the MvhB and FmdF interface. Side chains are shown as sticks. Atoms within H-bond distance are marked with cyan dashed lines. Abbreviations are defined in the legend of Fig. 2.

Our cryo-EM analysis shows that the (Mvh-Hdr)_2_-Fmd_4_ complex contains two MvhB subunits connecting HdrA to FmdF (Fig. 1 and fig. S4), confirming that MvhB plays a similar role to FmdF in mediating direct transfer of low-potential electrons. Our results further suggest that our previous observation of Elp, Hdr, Fmd, and MvhB components copurifying is the result of a similar complex, (Elp-Hdr)_2_-Fmd_4_, being formed in *M. marburgensis* under nickel-limited conditions (*17*). MvhB has six ferredoxin-like domains (Fd1 to Fd6), with domains Fd2 and Fd6 inserted between Fd1 and Fd5 (Fig. 7A). The protein binds a total of 12 Fe-S clusters: 10 [4Fe-4S] clusters via the canonical CX_2_CX_2_CX_3_CP motif, a [4Fe-4S] cluster via an elongated CX_2_CX_5_CX_3_CP motif in Fd2, and a [3Fe-4S] cluster via a CX_2_IX_2_CX_3_CP motif in Fd5 (Fig. 7, A and B). In some species of Class I methanogens, the [3Fe-4S]-binding motif is present as a canonical [4Fe-4S]-binding motif or a motif containing a single carboxylate at the position of Ile^288^ in *M. marburgensis* (fig. S28). The interaction between HdrA and MvhB occurs through MvhB’s Fd2 and HdrA’s ferredoxin-like domain, bringing clusters MB1 and HA6 close enough for rapid electron transfer (9.8 Å; Fig. 7, C to E). FmdF domain Fd1 interacts with the MvhB Fd4 domain, bringing MB6 and FF1 near enough for electron transfer (9.8 Å; Fig. 7, C, F, and G). Although many residues contribute to these interactions via hydrogen bonds and salt bridges (Fig. 7, C to F), only some residues, including Lys^53^ and Glu^224^, are conserved in Class I methanogens (fig. S28), suggesting nonconserved interactions should also be important for complex formation. AlphaFold 3 (AF3) accurately predicted the interaction between *M. marburgensis* HdrA, MvhB, and FmdF with a root mean square deviation (RMSD) of 1.8 Å (fig. S29, A and B). Predicting the full complex presented challenges due to its size and to the lack of a priori knowledge of subunit stoichiometry. Nevertheless, the predicted interaction of HdrA, MvhB, and FmdF likely provides a good proxy for the formation of a similar structure in other species, and such an interaction was predicted for other methanogens encoding *mvhB*, supporting a role of MvhB in mediating the formation of (Mvh-Hdr)_2_-Fmd_4_ complexes in Class I methanogens (fig. S29C). In contrast, AF3 did not predict an interaction between *M. marburgensis* HdrA and FmdF, suggesting that this interaction, observed in the D3-symmetric (Fdh-Hdr-Fmd)_6_ supercomplex of *M. hungatei*, a Class II methanogen (fig. S30), is unlikely to occur in *M. marburgensis* (Class I).

One exception within Class I methanogens is the hyperthermophilic *Methanopyrus kandleri*, which can grow at 110°C (*21*) and lacks an *mvhB* gene in the *mvh* operon. Instead, a truncated MvhB-like polyferredoxin, which lacks the Fd5 and Fd6 domains, is present elsewhere in the genome (fig. S27) (*22*). We investigated the possibility of a similar complex in *M. kandleri* by anion-exchange chromatography and/or size exclusion chromatography (SEC) and ammonium sulfate fractionation (Supplementary Text and figs. S31 to S34). In these experiments, the Mvh, Hdr, and Fmd subunits were coeluted with the MvhB-like polyferredoxin. In spite of low sequence similarity to the *M. marburgensis* MvhB, AF3 predicts a structure of the MvhB-like protein with a fold similar to the Fd domains contributing to the electron transfer between HdrA and FmdF in MvhB and predicts a similar complex between HdrA, MvhB, and FmdF (fig. S35). On the basis of these, we hypothesize that *M. kandleri* uses the truncated MvhB-like polyferredoxin for transfer of electrons from FBEB to CO_2_ fixation. We note that such a complex would lack the branching electron transfer pathway observed in *M. marburgensis*, discussed below.

**Fig. 8.**
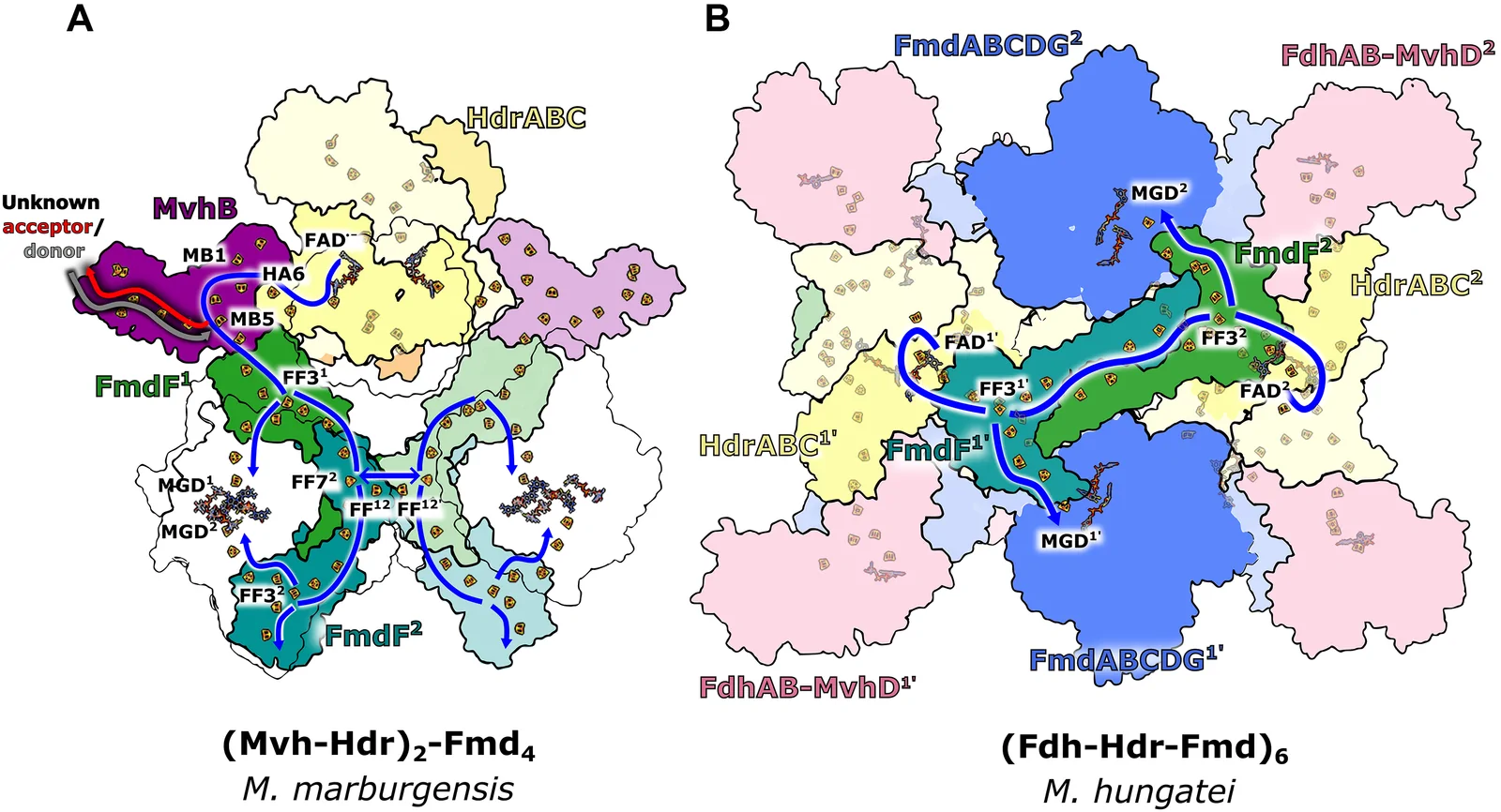
Comparison of the direct electron transfer paths from bifurcating FAD to CO_2_ reduction. In both the (Mvh-Hdr)_2_-Fmd_4_ and the (Fdh-Hdr-Fmd)_6_ supercomplexes, a strongly reducing electron donated by the semiquinone state of the bifurcating flavin (FAD^•−^) in HdrA can be transferred to the CO_2_-reducing sites of the Fmd subcomplex (blue arrows). (**A**) In the case of the (Mvh-Hdr)_2_-Fmd_4_, two FAD molecules in the HdrA dimer are electronically connected to four CO_2_-reducing sites. In addition, electrons may flow from (black arrow) or to (gray arrow) MvhB through an electron path formed by the clusters at the C-terminal domains Fd3, Fd5, and Fd6. (**B**) In contrast, the (Fdh-Hdr-Fmd)_6_ complex provides a path from two FAD molecules in the HdrA dimer to two CO_2_-reducing centers. Abbreviations are defined in the legend of Fig. 2.

The possible pathways for electron transfer in the C2 (Mvh-Hdr)_2_-Fmd_4_ and the D3 (Fdh-Hdr-Fmd)_6_ supercomplexes are compared in Fig. 8. Although the architecture of these supercomplexes is different, low-potential electrons can be transferred from two HdrA domains to the FmdF polyferredoxin and distributed to at least two CO_2_-reducing sites in (Fdh-Hdr-Fmd)_6_ or up to four sites in (Mvh-Hdr)_2_-Fmd_4_. Unexpectedly, only 4 of 12 Fe-S clusters of MvhB are part of the electron pathway from HdrA to FmdF (Fig. 8A); the other clusters appear to form two branching pathways to Fd2 and Fd5/Fd6 (Figs. 7, B and C, and 8A). In particular, the C-terminal region of MvhB consisting of Fd5 and Fd6, connected electronically via Fd3, appears not to be required either for electron transfer from Hdr to Fmd or for structural reasons. In our structures, these domains appear less well resolved due to local flexibility (figs. S3 and S9). Given that these domains have not been eliminated by simple truncation of the *mvhB* gene, they likely have functional importance. We predict a role for these domains in transferring low-potential electrons with partner proteins in the cell. A main candidate would be ferredoxin, which transfers low-potential electrons by diffusing in the cytosol (*10*). It is also possible that MvhB can form dimers, producing higher-order assemblies that could have been lost during the purification procedure. AF3 predicts dimer formation between the C-terminal domains of MvhB with high confidence (fig. S29, D and E). Other potential electron acceptors include redox enzymes requiring low-potential electrons (e.g., carbon monoxide dehydrogenase, pyruvate:ferredoxin oxidoreductase, or the activation enzyme system for the methane-forming enzyme methyl-coenzyme M reductase) (*23*, *24*). The site may also serve as an alternative entry point of low-potential electrons from other ferredoxin-reducing enzymes (Fig. 8A), like the membrane-associated hydrogenases Eha and Ehb. These hydrogenases, less abundant than the Mvh-Hdr-Fmd complexes (*17*), contain polyferredoxin subunits (*18*) and provide low-potential electrons by oxidation of H_2_ coupled to dissipation of the chemiosmotic gradient (*25*). Specifically, Eha seems to be directly involved in hydrogenotrophic methanogenesis by providing electrons for CO_2_ fixation via Fmd in an anaplerotic manner (*26*).

### The dimeric and tetrameric FmdF covaries with the absence or presence of MvhB

The Hdr-Fmd complexes from *M. marburgensis* and *M. hungatei* show major differences in quaternary structure (Fig. 8 and fig. S23). As the crucial interfaces of the high-order assembly are formed by subunits HdrA, FmdF, and MvhB, we reasoned that changes in the encoded sequence of FmdF and HdrA, in addition to the presence of MvhB, could explain the disparate Hdr-Fmd complexation in *M. marburgensis* (Class I methanogen) and *M. hungatei* (Class II methanogen). First, we analyzed the amino acid sequences and the AF3-predicted structure of FmdF homologs. We found that FmdFs classified into six major structural groups whose differences resided mainly in the number of Fd domains and the length and sequence of loops 1 and 2 connecting Fd2 and Fd3 (figs. S36 to S40). All Class I methanogens encode a Group 1 FmdF, which includes two conserved cysteine residues in loop 2. These residues bind a [4Fe-4S] cluster upon dimerization (“FF9”; Fig. 2B and fig. S36) (*8*) and are part of the tetramerization interface observed in *M. marburgensis* and *M. wolfeii* FmdF (fig. S38A). We note that AF3 does not correctly predict a tetramer for some FmdFs of Group 1, but because this includes the FmdF of *M. marburgensis*, shown here to form a tetramer, we infer that the AF3 prediction is not reliable in this case (fig. S38E). Because the presence of the FF9 [4Fe-4S] cluster appears to be essential for the tetramerization of FmdF, and because the cluster-ligating function of these cysteine residues requires tetramerization, we use the presence of the cysteine residues in loop 2 as the most reliable indicator of a tetrameric FmdF.

Methanomicrobiales and Methanocellales (Class II methanogens) encode Group 2 FmdFs. Members of this group, represented by *M. hungatei* FmdF, lack the loop 2 cysteines that bind the dimer-bridging [4Fe-4S]-cluster FF9 and, in *M. hungatei*, enable the formation of a hexameric supercomplex without MvhB (*15*). The distribution of Group 1 FmdFs in Class I methanogens and Group 2 FmdFs in Methanomicrobiales and Methanocellales agrees with the prediction, based on the presence or absence of an *mvhB* gene, of the formation of C2-symmetric Hdr_2_-Fmd_4_ (fig. S29) and D3-symmetric (Hdr-Fmd)_6_ complexes in these methanogenic groups, respectively, and AF3 predictions of HdrA-(MvhB-)FmdF subcomplexes from several species of each group are consistent with this prediction (figs. S29 and S30). AF3 analysis indicated the presence of Group 3–6 FmdFs, which are not present in either Class I methanogens or the Class II Orders Methanomicrobiales and Methanocellales. Group 3–6 FmdFs are predicted to form monomers or truncated dimers (figs. S38 and S41 and Supplementary Text), which are inconsistent with the C2-symmetric or D3-symmetric supercomplexes characterized here and previously (*15*). The structural and functional roles of FmdF in the microorganisms encoding Group 3–6 FmdFs have not been characterized, but it seems unlikely that these organisms form supercomplexes with an architecture similar to Groups 1 and 2.

### Complex evolutionary history of the FmdF polyferredoxin

Phylogenetic analysis of the FmdF polyferredoxin subunit suggests that FmdF in Class I methanogens evolved vertically from the ancestral methanogen; however, the FmdF sequences in Methanomicrobiales and Methanocellales (Class II) originated from Asgardarchaea or Bathyarchaeales via horizontal gene transfer (fig. S42). We sought to construct a phylogenetic tree using an outgroup to confirm the direction of these horizontal gene transfers. To achieve this, we divided the FmdF sequence into its four ferredoxin domains and used this dataset to create a phylogenetic tree (fig. S43). As each ferredoxin domain within the protein can be treated as a separate paralog, all the domains in this tree can be used to root each other. This confirms that the root of FmdF was likely present in the last common ancestor of TACK and Euryarchaea, which is presumed to have been a hydrogenotrophic methanogen. Subsequently, the history of the gene becomes more complex and nonvertical. The Class II FmdF branch is a sister group to the TACK sequences. This suggests that these organisms underwent an ancient horizontal gene transfer, acquiring an FmdF that was probably unable to support tetramerization, as is observed in Class I methanogens.

We next used ancestral sequence reconstruction to ask whether the last common ancestor of all FmdFs would have been capable of this interaction. To do this, we inferred the sequence of the last common ancestor of all Fd3 domains on our Fd-domain tree. Because the bridging interaction of conserved cysteines in the Fd3 domain is a key factor in the FmdF tetramerization mechanism associated with a C2-symmetric complex, we asked whether the ancestral state of those sites in the Fd-domain phylogeny support the presence of such an interaction in the FmdF last common ancestor. On our maximum likelihood tree, our ancestral sequence reconstruction analysis showed both sites as cysteines with high confidence (fig. S44). On this tree, however, an *Archaeoglobus* Fd3 domain was placed as a sister group to all other Fd3 and Fd2 sequences, which would require a horizontal transfer of this Fd3 domain from a very early archaeon to the stem lineage of *Archaeoglobus*. An alternative explanation for this branching order is that it is an artifact resulting from either long-branch attraction or a lack of phylogenetic signal. In this case, the *Archaeoglobus* Fd3 could bias the inference of ancestral states for the common ancestor of all FmdFs because the *Archaeoglobus* Fd3 domain contains both cysteines. Accordingly, removing the *Archaeoglobus* Fd3 clade from the tree lowered our confidence in the second cysteine site as the posterior probability decreased to 0.67, whereas the first cysteine is still inferred to have been present with very high confidence (fig. S45). On balance, the deep root of FmdF in the archaeal phylogeny, the nonvertical relationships of FmdF in Class II methanogens and the strict conservation of the key cysteine residues in modern Class I methanogens suggest the presence of a supercomplex in the ancestor of all methanogens with a Class I type architecture. In this scenario, Class I methanogens conserved the ancestral architecture whereas Class II methanogens acquired a different architecture, likely enabled by a horizontal transfer of FmdF from TACK archaea. More structures of diverse Hdr complexes, in particular from TACK archaea, and/or increased availability of sequencing data, should allow this theory to be further tested in the future.

## DISCUSSION

Large supercomplexes of methanogenic enzymes allow strongly reducing electrons from electron bifurcation to be directed to the energetically demanding step of CO_2_ reduction and fixation, reducing the likelihood of wasteful side reactions that could occur when electrons are carried by a diffusible electron donor. A potential disadvantage of such architectures is that they define a fixed stoichiometry between the different redox modules, in this case, the ratio between hydrogen/formate-oxidizing and CO_2_-fixing enzymes. Such rigid architectures may constrain adaptability to differing substrate availabilities. Our structural, phylogenetic, and ancestral reconstruction analyses suggest that, over the course of methanogen evolution, the ability to form electron-bifurcating, CO_2_-reducing supercomplexes has been maintained, whereas the architecture and subunit stoichiometry of the supercomplexes have been altered. We therefore asked the question whether this evolutionary change could provide a physiological advantage in the distinct environments inhabited by methanogens with the different supercomplex stoichiometries. Available physiological and ecological evidence suggests that several Methanomicrobiales and Methanocellales (Class II) species are capable of growth at very low electron donor availability (H_2_ or formate), in some cases close to the thermodynamic limit of hydrogenotrophic methanogenesis, as inferred from the natural environments where these species are found and the culture conditions used for their isolation (*27*, *28*). We therefore speculate that, under environments of limited electron donor availability such as those occupied by many Class II methanogens, the 1:1 stoichiometry of Hdr (electron input) to Fmd (electron output) modules dictated by the D3-symmetric supercomplexes may confer a competitive advantage over the 1:2 stoichiometry imposed by the C2-symmetric complex described here for Class I methanogens by allowing them to maintain a higher total rate of H_2_ oxidation.

Another difference, whose physiological relevance is difficult to evaluate with the present data, is that the C2-symmetric complex appears to allow electron transfer (ET) with other cellular partners through the branching paths for ET through MvhB (Figs. 7C and 8A). In contrast, the hexameric complex of Class II methanogens does not present any obvious points for electron exchange with other partners. Notably, in spite of the differences, both groups use polyferredoxin proteins (FmdF alone or with MvhB), highlighting the universal advantage of such “nanowires,” recently also shown to be involved in CO_2_ reduction in acetogens (*29*), for direct ET between redox enzymes. It is perhaps unexpected that the Class II hexameric structure shows no obvious points for electron exchange with other partners in the cell, given that such a function could aid survival of Methanomicrobiales and Methanocellales, for example, by allowing entry of electrons from Eha/Ehb-type complexes (*26*). However, we note that the purified Fdh-Hdr-Fmd supercomplex did reduce soluble ferredoxin (*15*), suggesting that the D3 supercomplex may also interact with other electron carriers, although the structural basis is unclear.

We hypothesize that the last common ancestor of methanogens had conserved cysteine residues in FmdF and thus formed a C2-symmetric supercomplex (figs. S44 and S45). The last archaeal common ancestor is suggested to be a (hyper)thermophile (*30*, *31*) that inhabited environments similar to those inhabited by basal Class I methanogens, namely, deep-water hydrothermal vents with high concentrations of H_2_ provided by geological processes (*32*, *33*). Among the Class I methanogens, the hyperthermophilic *M. kandleri* contains an MvhB-like polyferredoxin that is shorter than that from other Class I methanogens (fig. S35, B and C) that we show here to cofractionate with Mvh, Hdr, and Fmd proteins. The AF3-predicted structure of this MvhB-like polyferredoxin shows a fold similar to the Fd domains participating in electron transfer between HdrA and FmdF but lacking the Fd5 and Fd6 domains that form a branching path for electrons (fig. S35, B and D). On the basis of these data, we hypothesize that the ancestor of hydrogenotrophic methanogens, growing at high H_2_ concentrations, used a similar truncated polyferredoxin. Evolution, likely via duplication, then led to elongation of MvhB polyferredoxin to allow transfer of electrons with other partners in the cell or via a dimeric MvhB-MvhB bridge. These observations highlight the relative ease with which functional and architectural changes in such supercomplexes can be mediated by simple mutational pathways, including through duplication, gene fusion, and horizontal gene transfer of electron-transferring subunits.

## MATERIALS AND METHODS

### Materials

*M. marburgensis* DSM 2133 was purchased from the German Collection of Microorganisms and Cell Cultures (DSMZ). *M. kandleri* AV19 cells were a gift from K. O. Stetter (University of Regensburg). Most chemicals are from Sigma-Aldrich (Taufkirchen). Heterodisulfide (CoM-S-S-CoB) was synthesized as described previously with some modifications (*10*, *34*).

### Cultivation methods

Cultivation of *M. marburgensis* was performed as reported previously (*35*). The standard medium of *M. marburgensis* contains KH_2_PO_4_ [6.8 g/liter (50 mM)], Na_2_CO_3_ [2.544 g/liter (24 mM)], NH_4_Cl [2.12 g/liter (40 mM)], 0.2 mM MgCl_2_·6H_2_O, 50 μM FeCl_2_·4H_2_O, 5 μM NiCl_2_·6H_2_O, 1 μM CoCl_2_·6H_2_O, 1 μM NaMoO_4_·2H_2_O, and Titriplex I (0.09 g/liter). A concentrated trace element solution containing 0.2 M MgCl_2_·6H_2_O, 50 mM FeCl_2_·4H_2_O, 5 mM NiCl_2_, 1 mM CoCl_2_·6H_2_O, 1 mM NaMoO_4_·2H_2_O, and Titriplex I (90 g/liter) was prepared separately and adjusted to pH 6.7 by addition of NaOH. The trace element solution (0.1%, v/v) was added to the medium. A 0.2% water solution of resazurin sodium salt was lastly added to the medium (final concentration: 0.6 mg/liter).

We used a 10-liter jar fermenter for the cultivation of *M. marburgensis*, which was agitated at 1000 rpm at 65°C. For cultivation, 80% H_2_/20% CO_2_/0.2% H_2_S mix gas was supplied (2 liters/min) without overpressure. The cells were harvested by anaerobic centrifugation using a Beckman JA-25.50 rotor at 13,000*g* for 15 min at 4°C.

### Purification of the Mvh-Hdr-Fmd complex from *M. marburgensis*

All steps were performed anaerobically in an anaerobic chamber under 3 to 5% H_2_ in N_2_ (Coy Laboratories, Grass Lake, MI, USA). The purification was performed as described previously (*19*). Frozen or fresh *M. marburgensis* cells (20 g) were suspended in 20 ml of 50 mM tris-HCl (pH 7.6) containing 2 mM dithiothreitol (buffer A). The cell suspension was subjected to ultrasonication on ice/water for 2 min using a SONOPULS GM200 (Bandelin, Berlin, Germany) with 72D tip with 60% cycles 12 times, with 2-min breaks between sonication cycles. The supernatant was collected by centrifugation in a Sorvall WX Ultra centrifuge (Thermo Fisher Scientific, Dreieich, Germany) with a T-880 rotor at 41,000 rpm for 30 min at 4°C. The supernatant (cell extract) contained 670 mg of protein (16.7 mg/ml). All the supernatant was loaded on a Q Sepharose column (HiTrapQ HP, 5 ml, Cytiva) equilibrated with buffer A. The proteins bound on the column were eluted with a step gradient using 50 mM tris-HCl (pH 7.6) containing 2 mM dithiothreitol and 1 M NaCl (buffer B). The step gradient was 30, 40, 44, 48, 52, 56, 60, and 100% buffer B with a flow rate of 2 ml/min. The proteins with Hdr activity were mainly eluted in the 48% buffer B fraction. The fraction was collected and diluted with the same volume of 50 mM tris-HCl (pH 7.6), containing 2 mM dithiothreitol and 1.2 M ammonium sulfate (buffer C). The diluted sample was loaded on a Phenyl Sepharose column (HiTrap Phe-HP, 5 mL, Cytiva) equilibrated with buffer C. The proteins bound on the column were eluted with a step gradient using 50, 58, 67, 83, and 100% buffer A with a flow rate of 2 ml/min. The buffer solution of the 33% buffer C fraction of the Phenyl Sepharose column containing 0.4 M ammonium sulfate was exchanged with 50 mM tris-HCl (pH 7.6) containing 2 mM dithiothreitol and 150 mM NaCl by an Amicon Ultra 0.5 (3-kDa cutoff) filter. The sample was lastly concentrated to ∼0.5 ml, and half of the sample was applied to an SEC column, Superose 6 Increase (10/300 GL, 24 ml, Cytiva), and eluted with a flow rate of 0.5 ml/min. The SEC column was calibrated with thyroglobulin (bovine) at 670 kDa, γ-globulin (bovine) at 158 kDa, ovalbumin (chicken) at 44 kDa, myoglobin (horse) at 17 kDa, and vitamin B12 at 1.35 kDa. The standard materials were from Bio-Rad (Feldkirchen, Germany).

### Fractionation of proteins from *M. kandleri*

In the first experiment, frozen *M. kandleri* cells (4.9 g) were suspended in 5 ml of buffer A. The cells were disrupted by three passes through a French Press Cell Disrupter (Thermo Electron Corporation) at 110 MPa. The unbroken cells and cell debris were removed by centrifugation using a Beckman JA-25.50 rotor at 27,000*g* for 30 min at 4°C. The supernatant was subjected to ultracentrifugation using a Beckman Coulter 70Ti rotor at 150,000*g* for 2 hours at 4°C. The supernatant was applied to an anion-exchange chromatography column (HiTrapQ HP, 5 ml, Cytiva) equilibrated with buffer A. After washing with 20 ml of buffer A, proteins were eluted with a 180-ml linear gradient of NaCl (0 to 1 M) at a flow rate of 2 ml/min. The eluted solution was collected each 7.5 ml and used for the enzyme assay and mass spectrometry (MS)–based proteomic analysis. The fractions were analyzed by the Fmd and Hdr activities and by SDS–polyacrylamide gel electrophoresis (PAGE) using 8 to 16% gradient polyacrylamide gel from Bio-Rad Laboratories. In the first purification, the Q Sepharose fractions were subjected to proteomic analysis.

In the second experiment, the 5.6-g *M. kandleri* cells were suspended in 5-ml buffer A and disrupted by six passes through a French Press Cell Disrupter at 110 MPa. Q Sepharose chromatography was performed as described for the first purification, and the fractions exhibiting the Hdr activity were pooled; the buffer was exchanged into buffer A containing 2 mM dithiothreitol and 150 mM NaCl in an Amicon Ultra 0.5 (3-kDa cutoff) filter. The sample was lastly concentrated to ∼0.5 ml and applied to an SEC column, Superose 6 Increase (10/300 GL) and eluted at a flow rate of 0.5 ml/min. The eluate was collected in 0.5-ml fractions. The SEC fractions were subjected to SDS-PAGE and proteomic analysis. The SEC column was calibrated with thyroglobulin (bovine) 670 kDa, γ-globulin (bovine) 158 kDa, ovalbumin (chicken) 44 kDa, myoglobin (horse) 17 kDa, and vitamin B12 1.35 kDa. The standard materials were from Bio-Rad (Feldkirchen, Germany) as described previously (*17*).

In the third experiment, the 2.0-g *M. kandleri* cells were suspended in 2-ml buffer A and disrupted by six passes through a French Press Cell Disrupter at 110 MPa. Unbroken cells and cell debris were removed by centrifugation using a Beckman JA-25.50 rotor at 27,000*g* for 30 min at 4°C. The supernatant was designated as cell extract in this experiment. The cell extract was subjected to fractionation by ammonium sulfate precipitation, in which ammonium sulfate powder was added into the cell extract on ice. After a ten-minute incubation on ice, the solution was centrifuged using a Beckman JA-25.50 rotor at 27,000*g* for 30 min at 4°C. The supernatant was further precipitated by addition of ammonium sulfate powder in the same manner. The concentrations used for the fractionation were 40, 60, 80, and 100% saturation of ammonium sulfate. The supernatant of each fraction was subjected to the Fmd and Hdr activity assay. The 60% ammonium sulfate precipitated fraction, which contained major Hdr activity, was concentrated, and the buffer was exchanged with buffer A containing 2 mM dithiothreitol and 150 mM NaCl in an Amicon Ultra 0.5 (30-kDa cutoff) filter. The sample was lastly concentrated to ∼0.5 ml and applied to an SEC column, Superose 6 Increase (10/300 GL), and eluted at a flow rate of 0.5 ml/min. The eluate was collected in 0.5-ml fractions. The SEC fractions were subjected to SDS-PAGE and proteomic analysis.

### Cryo-EM sample preparation and data collection

The cryo-EM sample preparation was performed immediately after the Superose 6 Increase (10/300 GL) purification step inside an anaerobic chamber (<1 part per million O_2_; Coy Laboratory Products). The fraction corresponding to the 1-MDa peak was used for freezing (fig. S1). For each grid (glow-discharged UltrAuFoil 1.2/1.3 300 mesh), 3 μl of the sample (1.2 mg/ml) was applied, blotted for 4 to 5 s with Whatman 595 filter paper (Sigma-Aldrich) at 4°C under 100% humidity, and plunge frozen in liquid ethane using a Vitrobot Mark IV (Thermo Fisher Scientific). Two datasets from two different grids (9633 and 9144 movies) were collected at 215,000x magnification using aberration-free image shift (AFIS) (*36*) with a Titan Krios G4 equipped with a cold field-emission gun, Selectris X filter, and Falcon 4 direct electron detector. EPU (version 3.2) was used for automated data acquisition of dose-fractionated movies with a total dose of 60 e^−^/Å^2^ in electron-event representation (EER) format (*37*) and a defocus range between −0.8 and −2.2 μm.

### Image processing and model building

A detailed workflow of the steps for data processing is displayed in figs. S2 and S3. An initial dataset of 9633 movies (with 854 EER frames) was processed by importing the movies into RELION-4.0 (*38*). The raw movies were fractionated to 0.98 electrons/Å^2^ per frame and motion corrected and dose weighted using RELION’s implementation of MotionCor2 (5 x 5 patches) (*39*). CTF parameters were estimated using CTFFIND 4.1 (*40*). Corrected micrographs were then selected on the basis of a maximum 4.0-Å CTF resolution estimate, and micrographs with substantial crystalline ice contamination were manually removed. A total of 5882 curated micrographs were then used for particle picking. An initial set of particles was picked by RELION’s Laplacian-of-Gaussian (LoG) picker (250 and 500 Å in minimum and maximum diameters, −3.0 and 5.0 default and upper thresholds). A total of 163,206 particles were picked and extracted using a box size of 700 pixels downscaled to 180 pixels. The particles were imported into Cryosparc v.4.1 (*41*) and subjected to 2D classification (50 classes, default). A total of 21 classes corresponding to 52,683 particles were selected. The particles were reimported into RELION-4.0, reextracted, and used to train a Topaz model (*42*) (300 Å in diameter, 70 particles per micrograph), which allowed 525,622 particles to be picked. These particles were reduced to 222,870 during extraction (700-pixel box down to 180 pixels) by applying a figure-of-merit (FOM) cutoff of −2.5. The particles were imported into Cryosparc and subjected to 2D classification, and 23 classes corresponding to 174,983 particles were selected and used for ab initio reconstruction (C1, two classes, default). One of the classes showed a reasonable map, and the corresponding particles were reimported into RELION and reextracted, and a random subset of 4000 particles was used to train a final Topaz model (250 Å in diameter, 200 particles per micrograph) (fig. S2A).

For the final data processing, the raw movies of the two datasets (854 and 847 EER frames and independent optics groups) were combined and fractionated to 0.98 and 0.99 electrons/Å^2^ per frame and motion corrected and dose weighted using RELION’s implementation of MotionCor2 (5 x 5 patches) (*39*). CTF parameters were estimated using CTFFIND 4.1 (*40*). Corrected micrographs were then selected on the basis of a maximum 4.0-Å CTF resolution estimate, and micrographs with substantial crystalline ice contamination were manually removed. A total of 12,279 micrographs were then used for particle picking with the Topaz model generated as described above. Of the 1,395,829 particles picked, 917,008 particles could be extracted using a 700-pixel box size downscaled to 352 pixels and an FOM value of −1.5. The particles were imported to Cryosparc and used for 2D classification (50 classes, default), from which 44 classes corresponding to 853,131 particles were selected and used for a two-class ab initio reconstruction job (C1, default). The maps obtained were further used as initial models for a two-class heterogeneous refinement job (C1, default). The map for one of the classes (361,297 particles) showed a clear density for the HdrABC dimer and blurred regions for the mobile arms and the Fmd subunits. The particles were then subjected to nonuniform (NU) refinement (C2, default), and the refined particles imported to RELION for Bayesian polishing. The polished particles were reextracted in a 700-pixel box size downscaled to 480 pixels and reimported in Cryosparc for NU refinement (C2, default), which gave a 2.61-Å map (consensus map of the complex) with blurred density for the different regions. The particles were first refined using masks for the HdrABC dimeric core or the Fmd tetramer, giving maps in which the mobile arm and one of the FmdABCG cores in the asymmetric unit remained fuzzy, indicating partial occupancy and/or flexibility. Particles were C2-symmetry expanded, imported to RELION, and subjected to focused 3D classifications without alignment followed by 3D refinements and CTF refinements, which allowed for the generation of high-resolution focused maps of all parts of the complex to be resolved (Fig. 1 and figs. S2 to S23), including the FmdF dimer, the FmdABCG^1^ and FmdABCG^2^ catalytic cores, and MvhB (figs. S2 to S4 and S7 to S9). For the mobile arm and the HdrABC dimer, the C2-expanded particles were subjected to 3D classification without alignment using six classes (*T* = 8, 25 iterations). Particles of each class showing clear density for the mobile arm were reimported to Cryosparc and subjected to local and CTF refinements. Two of the classes showed identical maps, and the particles were combined and used for final local refinements resulting in the focused and consensus maps of the mobile arm in state 2 substate a. The particle subsets of three other classes were further subjected to 3D classification without alignment (*T* = 100, 25 iterations) and Blush regularization using RELION-5.0 (*43*). Further local and CTF refinements in Cryosparc resulted in the focused and consensus maps of mobile arm in state 2 substate b (figs. S6 and S12) and mobile arm state 1 substates a and b (figs. S5, S10, and S11).

The focused maps corresponding to FmdABCG^1^ and FmdABCG^2^, FmdF dimer, MvhB, MvhB C-terminal region, and the MvhAGD-focused map of mobile arm state 2 were used for automatic model building using the machine learning–based tool ModelAngelo (*44*). For HdrABC subunits, the *M. marburgensis* HdrABC dimer [Protein Data Bank (PDB): 8RWN] (*17*) was used as initial model. The program Coot (*45*) was then used to place cofactors and to inspect and manually adjust the models. Then, the models of each region were rigid body fitted into the maps using ChimeraX (*46*) to generate combined models of the whole complex and the mobile arm in the different states. Last, composite maps were generated with Phenix (*47*) and its tool combine focused maps using as inputs: the consensus maps, the focused maps, and the combined models (figs. S4 to S6). Iterative rounds of Phenix real-space refinement and manual inspection and readjustment in COOT were performed to optimize the model stereochemistry and the fit to the cryo-EM density map as assessed with Phenix, MolProbity (*48*), and *Q*-score (*49*). The RMSD values were calculated by the matchmaker command of ChimeraX.

### AF3 predictions and sequence alignments

Prediction of the structure of protein complexes were performed using AF3 (*50*) via the AF3 server using default settings (https://alphafoldserver.com). The size of the predicted complexes was limited by the maximum token number of the AF3 server (5000 tokens). The sequences used for the prediction were obtained from the UniProtKB database (*51*) or the NCBI protein database (https://ncbi.nlm.nih.gov/protein/). A list of copy numbers and biological molecules for each run can be found in tables S3 and S4. Output models were assessed to determine whether a credible complex was generated, and subunit interfaces were inspected manually for surface complementarity and the absence of clashing atoms.

Sequence alignments shown in figs. S26, S28, S35, and S37 were performed using Clustal Omega and visualized using the ESPript 3.0 (*52*). The sequences used for each alignment are indicated in the figure or in the figure legend. For the analysis of the conservation of *M. marburgensis* His^68^ and Asp^71^ in other species, the sequences shown in fig. S26 were used. For the analysis of conservation of *M. marburgensis* HdrA Cys^202^ and Cys^205^, all the HdrA sequences shown in table S3 and the HdrA sequences from *Methanosarcina barkeri* (A0A0E3SIV6), *Methanosarcina acetivorans* (Q8TLB0; https://www.uniprot.org/uniprotkb/Q8TLB0/entry), and *Archaeoglobus profundus* (P84626; https://www.uniprot.org/uniprotkb/P84626/entry) were used. The LoGo images were generated using the Clustal Omega alignment as input of WebLogo3 (*53*).

### Hdr activity assay

Hdr activity was determined by recording the oxidation of reduced benzyl viologen (BV) with heterodisulfide (CoM-S-S-CoB) (*54*). For the assay, 0.7 ml of 800 mM potassium phosphate buffer (pH 7.0) was preincubated at 65°C for 5 min. Seven microliters of 200 mM BV was added to a 1-ml quartz cuvette (1-cm light path) to give a 2 mM final concentration, and then 10 to 20 μl of 20 mM sodium dithionite was added. Ten microliters of the sample was added to the cuvette. The enzyme reaction was started by addition of 7 μl of 100 mM CoM-S-S-CoB to give a 1 mM final concentration at 65°C. The activity was calculated using the extinction coefficient of BV at 578 nm (8.6 mM^−1^ cm^−1^) (*55*). One unit of enzyme activity is defined as oxidation of 2 μmol of BV/min.

### MS-based proteomic analysis

The protein concentration was measured using the bicinchoninic acid (BCA) method. Carbamidomethylation of the cysteines was performed using 5 mM tris(2-carboxyethyl)phosphine (TCEP)/100 mM ammonium bicarbonate at 90°C for 10 min and 10 mM iodoacetamide at 25°C for 30 min. Then, 50-μg aliquots of the samples were diluted to 0.5% DOC and digested overnight at 30°C with trypsin, MS approved (Serva). Before liquid chromatography–MS (LC-MS) analysis, samples were desalted using a Chromabond Spin C18 WP cartridge (Macherey-Nagel) according to the manufacturer’s instructions. Dried and reconstituted peptides were then analyzed using LC-MS carried out on an Orbitrap Exploris 480 instrument connected to an Ultimate 3000 RSLC nano and a nanospray ion source (Thermo Fisher Scientific). Peptide separation was performed on a reversed-phase high-performance liquid chromatography column (75 μm by 42 cm) packed with C18 resin (2.4 μm; Dr. Maisch) run with a 45-min gradient (0.15% formic acid/2% acetonitrile to 0.15% formic acid /50% acetonitrile). MS data were searched against an in-house *M. marburgensis* protein database using SEQUEST embedded into the Proteome Discoverer 1.4 software (Thermo Fisher Scientific). In the case of the analysis of the protein fraction of the SEC, the purified fraction was directly used for the MS-based analysis. Proteomic data were quantified using the DIA-NN software (*56*). To compare the intensity between different proteins, we calculated intensity-based absolute quantification (iBAQ) values (*57*).

### Phylogenomic analysis

A maximum likelihood tree based on the genomes of various archaea was inferred from a concatenated alignment of 53 archaeal single-copy marker genes generated by the GTDB toolkit (v. 2.4.0) (*58*, *59*) using the IQ-Tree 2 tool with LG+F+R4 best-fit substitution model selected by the ModelFinder tool with ultrafast bootstrap branch support (*60*).

### Structural classification, ancestral sequence reconstruction, and conformational analysis of the FmdF subunit

For structural classification, the structures of at least four FmdF sequences of the different phylogenetic groups were predicted by AF3 (tables S3 and S4). The predictions were carefully inspected, and the proteins were grouped depending on their domain composition and the presence of cysteines in domain Fd3.

The FmdF subunit maximum likelihood tree was inferred using IQ-TREE (version 2.2.2.7) (*61*) with WAG+G+I determined as the best fit model using the BIC criterion by ModelFinder (*60*). Branch support values were calculated using 10,000 replicates of the ultrafast bootstrap approximation (*62*). Ancestral sequence reconstruction was carried out through the empirical Bayes approach and marginal reconstruction option with the package phangorn (version 3.0) (*63*). Indel positions were identified using the parsimony method with gaps as an additional character state (*64*). We reconstructed the sequence of the ancestral Fd3 domain in two ways: first, on a tree in which we separated FmdF into its four independent ferredoxin domains. On this tree, the *Archaeoglobus* sequences branch outside of their respective Fd domain clades. In particular, *Archaeoglobus* Fd3 appears as sister to all other Fd3 domains, which is very incongruent with *Archaeoglobus*’ position in the archaeal species phylogeny. This is important because this Fd3 domain has the two cysteines that allow the formation of the tetramer. Because it branches outside of a group of Methanomicrobiales Fd3s (which do not have the cysteines) and Methanobacteriales (which have the cysteines), this anomalous placement of the *Archaeoglobus* Fd3 domain could inflate our confidence in the inference of two cysteines in the last common ancestor of Methanomicrobiales and Methanobacteriales Fd3 domains. On this tree, cysteines at positions 160 and 164 are inferred with posterior probabilities of 0.997 and 0.975, respectively. We therefore repeated the inference on the same tree topology but with the *Archaeoglobus* sequences removed. This yielded a lower confidence for the prediction of two cysteines because the cysteine at position 164 is now reconstructed with a posterior probability of 0.666 instead of 0.975.

## Supplementary Materials

### This PDF file includes:

Supplementary Text Figs. S1 to S47 Tables S1 to S5 References
