# Supplementary material for: Diversity of electron-bifurcating CO2-fixing supercomplexes in methanogens
Source: Sci Adv. 2026 Sep 16;12(38):eaed3711. doi: 10.1126/sciadv.aed3711 (PMC13580544; doi:10.1126/sciadv.aed3711)
Supplement: Supplementary file 1 [file sciadv.aed3711_sm.pdf]

Science Advances
AAAS

# Supplementary Materials for

## Diversity of electron-bifurcating $CO_2$-fixing supercomplexes in methanogens

Pablo San Segundo-Acosta *et al.*

Corresponding author: Georg Hochberg, georg.hochberg@mpi-marburg.mpg.de;
Seigo Shima, shima@mpi-marburg.mpg.de; Bonnie J. Murphy, bonnie.murphy@biophys.mpg.de

*Sci. Adv.* **12**, eaed3711 (2026)
DOI: 10.1126/sciadv.aed3711

**This PDF file includes:**

Supplementary Text
Figs. S1 to S47
Tables S1 to S5
References

**Supplementary text**

**Classification of FmdF**

FmdF was classified into six groups on the basis of sequence and predicted structure (fig. S36-S37). Group 1 includes FmdF in Class I methanogens and a subgroup in the order *Archaeoglobales*. The latter is fused at the C-terminus with a ferredoxin-like Fmd subunit (FmdG) containing two [4Fe-4S]-clusters, with a predicted architecture similar to the Group 1 FmdF-FmdG subcomplex in Class I methanogens. Phylogenetic analysis clusters *Archaeoglobales* Group 1 FmdF with those from Class I methanogens (fig. S44 and S45). The gene encoding *Archaeoglobales* Group 1 FmdF is present in a gene cluster that does not contain other *fmd* genes. These two features suggest that some sulfate-reducing *Archaeoglobales* obtained a Group 1 FmdF by horizontal gene transfer from *Methanopyrales.* In *Archaeoglobales*, HdrABC complexes probably contribute to sulfur metabolism but do not catalyse reduction of heterodisulfide (*65*), and it is unknown whether FmdF forms a complex with HdrABC in these species. AF3 does not predict complexation of FmdF and HdrA of *Archaeoglobus fulgidus*. Group 2 FmdFs are present in Methanomicrobiales and Methanocellales, Class II methanogens, and are represented by *M. hungatei*, in which FmdF dimers interact with HdrA dimers to form hexameric supercomplexes (Fig. 4B). Group 3 FmdFs, present in *Methanosarcinales,* lack the Fd3 ferredoxin domain inserted into Fd2 that forms the interface for dimerization. As in *Archaeoglobales* Group 1 FmdF, members of Group 3 are C-terminally fused to subunit FmdG. Group 4 FmdFs, present in *Methanosarcinales*, *Archaeoglobales*, and some members of the *Thermoproteota* phylum*,* have an Fd3 domain which is predicted to interact directly with Fd2, breaking the interface present in Group 1 and 3 FmdFs that allows domain-swapping-based dimerization (fig. S38D). The AF3-based predictions of monomeric and dimeric FmdF structures of Group 4 also suggested that this group do not form a dimer. *Methanosarcinales* genomes, in addition to encoding a Group 3 FmdF, encode a Group 4 member FmdF in a gene cluster containing an Hdr homolog (HdrA2) and none of the other Fmd-related genes. A complex between HdrA2 and Group 4 FmdFs is predicted by AF3 (fig. S41).

**Results of the fractionation of the Hdr complex from *M. kandleri***

In *M. kandleri*, the *mvhB* gene is not encoded in the *mvh* operon. Comparative genomic and AF3 analyses suggested that the MvhB-like polyferredoxin (Q8TWN1), which is encoded outside the operon, may play a role as an electron bridge in the predicted Mvh-Hdr-Fmd complex in this hyperthermophilic methanogenic archaeon (see fig. S35). To investigate the

role of the MvhB-like polyferredoxin in the Mvh-Hdr-Fmd supercomplex, the proteins from *M. kandleri* were fractionated and Hdr and Fmd activity was assessed. In *M. kandleri*, two isoenzymes of Fmd are encoded in the genome. The growth of *M. kandleri* requires tungstate, whereas molybdate does not support growth. Therefore, we expect both isoenzymes to contain tungstopterin. The gene sequences of the B subunits of the isoenzymes indicated that one contained selenocysteine, while the other contained a cysteine. These selenocysteine- and cysteine-containing isoenzymes are designated FwuB and FwcB, respectively(*66*). The fwu gene cluster contains the fwuABCDG genes. The polyferredoxin F subunit, which is designated here as FwuF, is encoded separately in the genome. The *fwcB* gene is also encoded outside the *fwu* gene cluster, in the absence of other fwd/fmd genes, which suggests that FwcB forms a functional hexameric enzyme with FwuACDGF. For simplicity, we will refer to these two formylmethanofuran dehydrogenase isoenzymes in *M. kandleri* as 'Fmd' as in the main text. However, the elution profiles of the subunits are depicted in fig. S31-S34 using their specific subunit names, 'FwuABCDFGH' and 'FwcB'.

In the first experiment, 4.9 g of *M. kandleri* cells were disrupted using a French press. The supernatant after ultracentrifugation, designated as cell extract, contained a much lower amount of protein (9.2 mg) than expected from the amount of the cells (Table S5), most likely due to the rigid pseudomurein cell wall of this archaeon (*21*). The cell extract exhibited Hdr (0.35 U/mg) and Fmd (0.47 U/mg) specific activities comparable to those from other methanogens (*54, 67, 68*). The cell extract was applied to an anion-exchange column (Q-Sepharose) and the proteins were eluted with a linear NaCl gradient. The Hdr enzyme activity was eluted as two peaks (fig. S31A). The yield of the Hdr activity was 25%, which indicates that substantial Hdr was lost during Q-Sepharose chromatography. The Fmd activity, which totaled only 9% of the activity measured in the cell extract, partially coeluted with the Hdr activity. The Q-Sepharose fractions exhibiting the Hdr and/or Fmd activities were subjected to proteomic analysis, which indicated that Hdr eluted in two separate fractions at 0.37–0.42 M and 0.51–0.60 M NaCl. The former and latter fractions contained enzymatically inactive and active Hdr, respectively. The proteomic intensity of Fmd subunits was found almost exclusively in enzymatically active Hdr fractions, though only low Fmd activity was detected (fig. S31B, C). The proteomic intensity of MvhGD was weak and comigrated with both enzymatically active and inactive Hdr fractions. MvhB-like polyferredoxin was found in both active and inactive Hdr fractions (fig. S31D). Another polyferredoxin-like Fe-S protein (Q8TXE9) was also eluted in the inactive Hdr fractions (fig. S31D). The hydrogenase large

subunit MvhA showed strong proteomic intensity and was mainly found in the fractions of the active Hdr fractions (fig. S31E).

In a second experiment, 5.6 g of *M. kandleri* cells were subjected to a more rigorous French press treatment. The protein amount in the cell extract (46 mg) was higher than in the first trial and the specific activity of Hdr (0.24 U/mg) was comparable to the first experiment. In contrast, the specific activity of Fmd (3.0 U/mg) was substantially higher than in the first experiment. The elution profile of the Hdr activity in Q-Sepharose chromatography was similar to the first experiment (fig. S32A). Most of the Fmd activity (~90%) was found in the flow-through fraction and ~10% of the Fmd activity was detected in the fractions eluted around 0.2 M NaCl. Proteomic analysis indicated again that the Hdr subunits eluted broadly in enzymatically inactive (~0.4 M NaCl) and active (~0.6 M NaCl) fractions (fig. S32B). The active Hdr fractions contained Fmd subunits but did not exhibit strong Fmd activity (Fig. S32C). MvhB-like polyferredoxin was eluted primarily with the active Hdr fractions, while the additional polyferredoxin-like Fe-S protein (Q8TXE9) eluted mostly with inactive Hdr (Fig. S32D). The MvhAGD proteins were found in fractions containing both the active and inactive Hdr proteins (fig. S32E).

The fractions exhibiting Hdr activity in the second Q-Sepharose chromatography (109–124 ml) were pooled and applied to a Superose-6 Increase size-exclusion chromatography (SEC) column. The Hdr activity and the proteomic intensities indicated that the HdrABC subunits are eluted with MvhAGD in a broad peak at 0.5–0.2 MDa (fig. S33A, E). In contrast, the Fmd subunits were eluted in fractions around 0.2 MDa and exhibited only weak enzymatic activity (fig. S33A, C). These findings suggest that the Mvh-Hdr from the Q-Sepharose fractions with the Hdr activity was eluted as an MvhAGD-HdrABC complex rather than as a supercomplex of Mvh-Hdr with Fmd, likely due to complex dissociation. The proteomic intensity of FmdF polyferredoxin eluted broadly across fractions at 1–0.2-MDa, which could be due to variable levels of complex dissociation. The highest proteome intensity of the MvhB-like polyferredoxin was observed in the smaller fractions (< 0.3 MDa); however, some traces of this 32 kDa protein were also found in the fractions containing Hdr and Mvh at 1–0.5-MDa (fig. S33D). These results suggest that MvhB-like polyferredoxin likely forms part of an Mvh-Hdr-Fmd supercomplex in *M. kandleri* but that the complex is fragile. Similar broad elution of MvhB was observed in the case of the Mvh-Hdr-Fmd and Elp-Hdr-Fmd complexes from *M. marburgensis* (*17, 19*). Coelution of an additional polyferredoxin-like Fe-S protein (Q8TXE9) observed broadly in fractions between 1–0.5

MDa, suggests the possibility that this Fe-S protein also interacts with the Mvh-Hdr-Fmd complex in *M. kandleri*.

Two fractionation experiments described above suggest that the possible Mvh-Hdr-Fmd complex in *M. kandleri* is unstable and decomposes during fractionation. These cells contain high concentrations of cyclic-2,3-diphosphoglycerate (cDPG) (> 1 M) as an osmotic solute, which stabilizes proteins inside the cell against high temperature (*69*), and the stability and/or activity of enzymes from *M. kandleri* depends on the presence of lyotropic salts (e.g. cDPG, potassium phosphate and ammonium sulfate) (*70*). Assuming that a postulated Mvh-Hdr-Fmd complex could be stabilized in the presence of high concentrations of a lyotropic salt, we performed fractionation of the *M. kandleri* proteins by ammonium sulfate precipitation followed by SEC.

In this experiment, we omitted the ultracentrifugation step, and the supernatant of a low-speed centrifugation of the French press lysate from 2.0 g of cells, containing 64 mg total protein, was used as the cell extract. The cell extract was fractionated by precipitation with 40%, 60%, 80% and 100% saturated solutions of ammonium sulfate. Most of the Hdr activity (~20% of the activity in the cell extract) was fractionated in 60% ammonium sulfate precipitation step (Table S5). In contrast, the Fmd activity (~20% of the activity in the cell extract) was observed across several precipitated fractions (60%, 80%, and 100% saturated solutions of ammonium sulfate). This finding indicated that most Hdr and Fmd sub-complexes are dissociated before or during ammonium sulfate precipitation. We applied the fraction precipitated by 60% saturated ammonium sulfate to SEC (fig. S34). Hdr activity assays and proteomic analysis of the SEC fractions indicated that MvhAGD, HdrABC, MvhB-like polyferredoxin, and Fe-S protein (Q8TXE9) were eluted as a broad peak at the elution volumes corresponding to 1.0–0.2 MDa (fig. S34B-E). The elution profile was similar to that found in the SEC experiments of the Mvh-Hdr-Fmd and Elp-Hdr-Fmd complexes from *M. marburgensis* (*17, 19*). FmdABCDG subunits were eluted in fractions corresponding to molecular weights less than 0.3 MDa. In contrast, the FmdF polyferredoxin subunit was eluted with MvhAGD, HdrABC and MvhB-like polyferredoxin, which suggests that FmdF is bound to the complexes. This finding supports the hypothesis that MvhB-like polyferredoxin and/or the additional Fe-S protein (Q8TXE9) form a complex with Mvh-Hdr and Fmd. The reason for elution of the Mvh-Hdr-MvhB-like-FmdF fraction at ~1 MDa SEC in spite of the lack of FmdABCDG subunits could be due to additional subunits including the additional Fe-S proteins.

Taken together, our fractionation experiments of proteins from *M. kandleri* using anion exchange and size exclusion chromatography, as well as salt-based precipitation, suggest the presence of a Mvh-Hdr-Fmd supercomplex, which involves MvhB-like polyferredoxin as an electron bridge. AF3 analysis predicts an interaction of the MvhB-like polyferredoxin in a bridging position between FmdF and HdrA (fig. S35). However, co-elution of an additional Fe-S protein (Q8TXE9) may indicate a different overall architecture of the supercomplex in *M. kandleri*. Notably, this Fe-S protein (Q8TXE9) is encoded in a large gene cluster containing the genes encoding pyruvate ferredoxin oxidoreductase (PFOR), acetyl-CoA synthase (ACS), and carbon monoxide dehydrogenase (CODH). In hydrogenotrophic methanogenic archaea, PFOR and CODH require low-potential electrons for production of pyruvate from acetyl-CoA and $CO_2$, and production of carbon monoxide from $CO_2$, respectively. Therefore, it is possible that this Fe-S protein (Q8TXE9) acts as a bridge between an Mvh-Hdr-Fmd supercomplex and other essential redox machineries in the cell, including PFOR and/or CODH. The proteomic data of SEC fractions in experiments 2 and 3 indicate the presence of ACS and CODH in the fractions at ~1 MDa with intensities similar to that of Fe-S protein (Q8TXE9) (fig. S46 and S47). In contrast, the subunits of PFOR were eluted in fractions containing smaller proteins (~0.2 MDa).

A

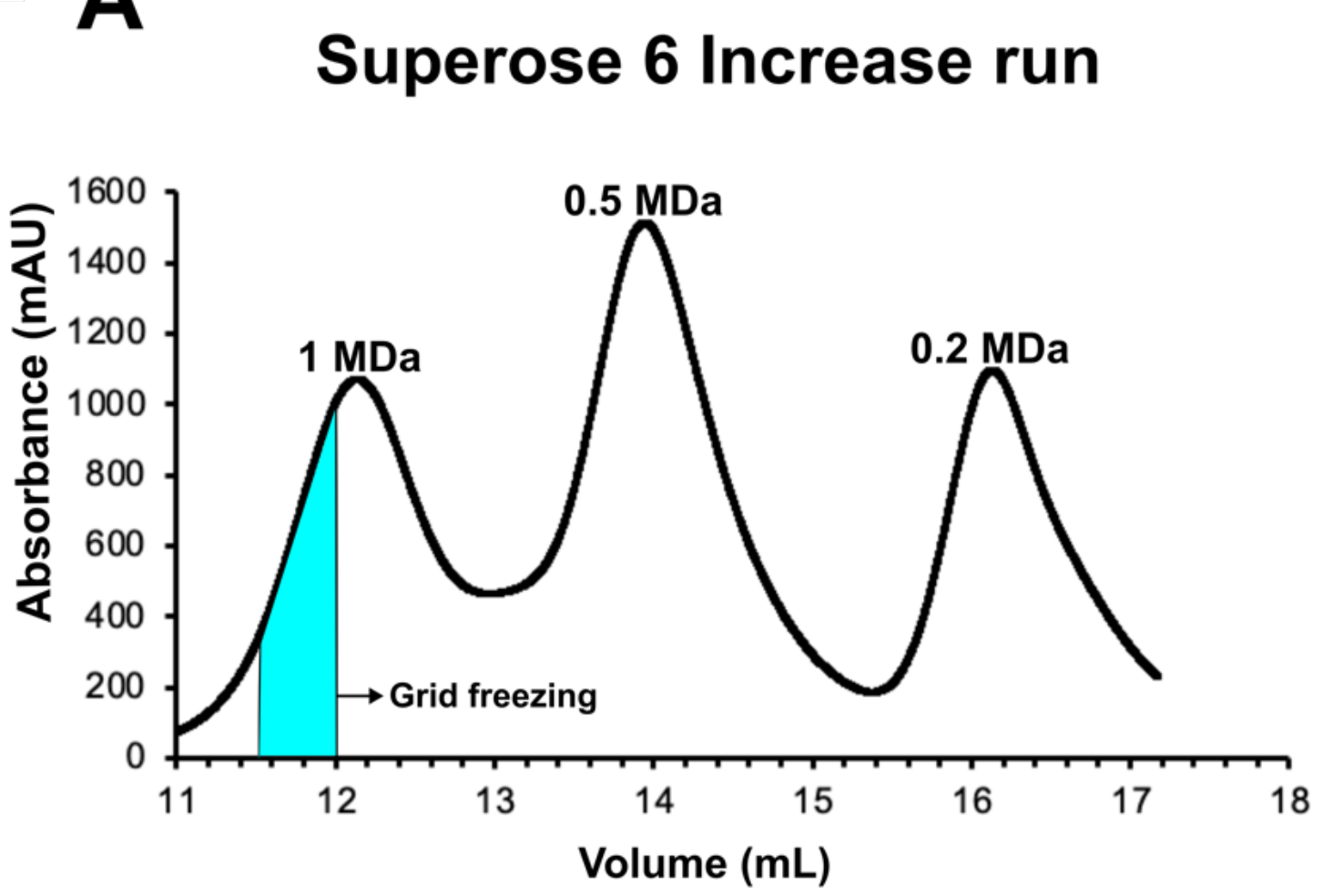


B

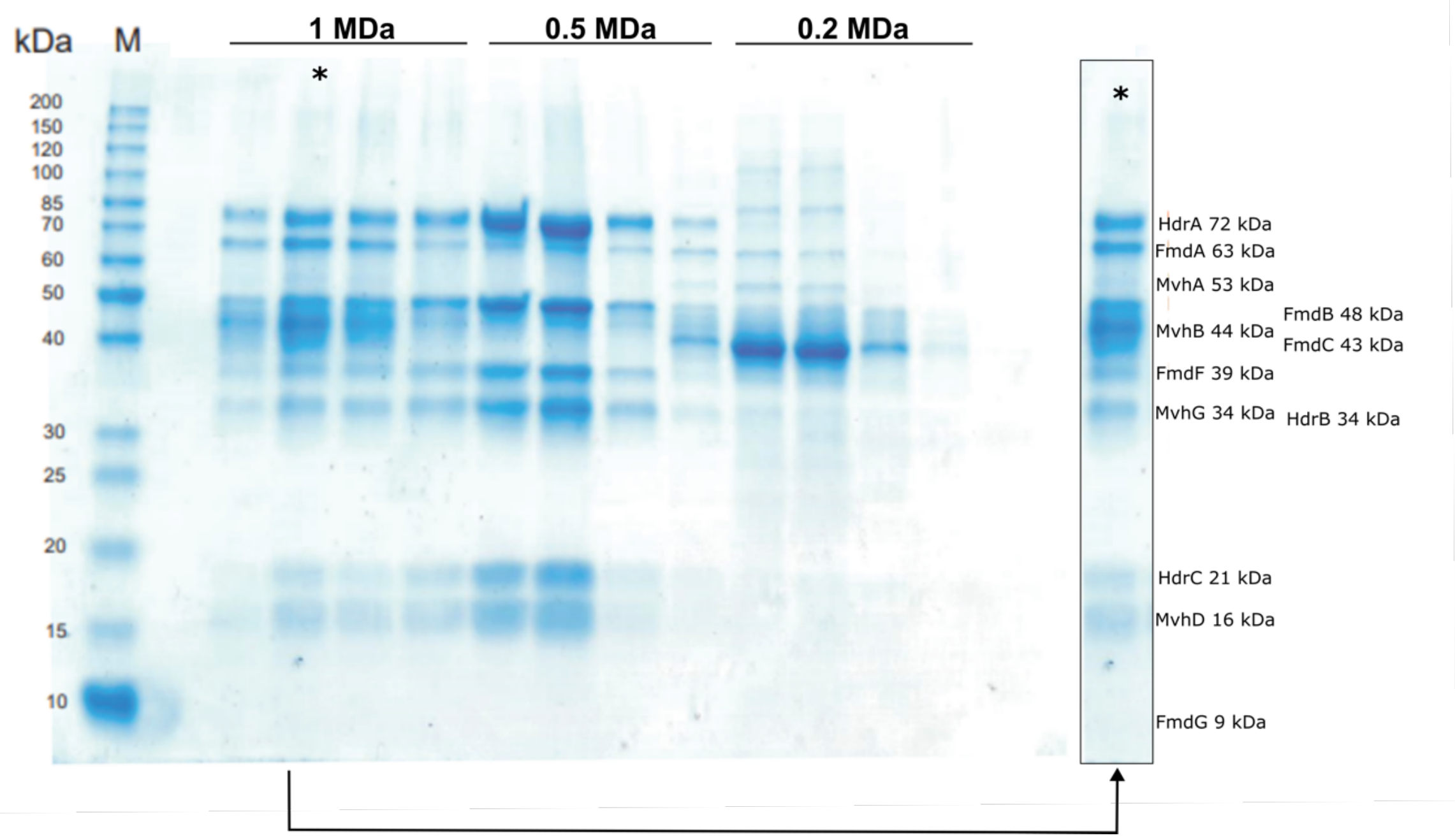


**Fig. S1. Purification of the Mvh-Hdr-Fmd complex for structural studies.** (**A**), Size-exclusion chromatography (SEC) profile using a Superose 6 Increase column. The 1-MDa fraction used for cryo-EM analysis is indicated. (**B**), SDS–polyacrylamide gel electrophoresis (PAGE) of the SEC fractions stained with Coomassie-blue. The lane corresponding to the fraction used for cryo-EM analysis is marked with *, and the expected identity of each band is indicated. (M) Protein-size ladder. The SEC elution peaks labelled in **A** are indicated above the corresponding gel lanes in **B**.

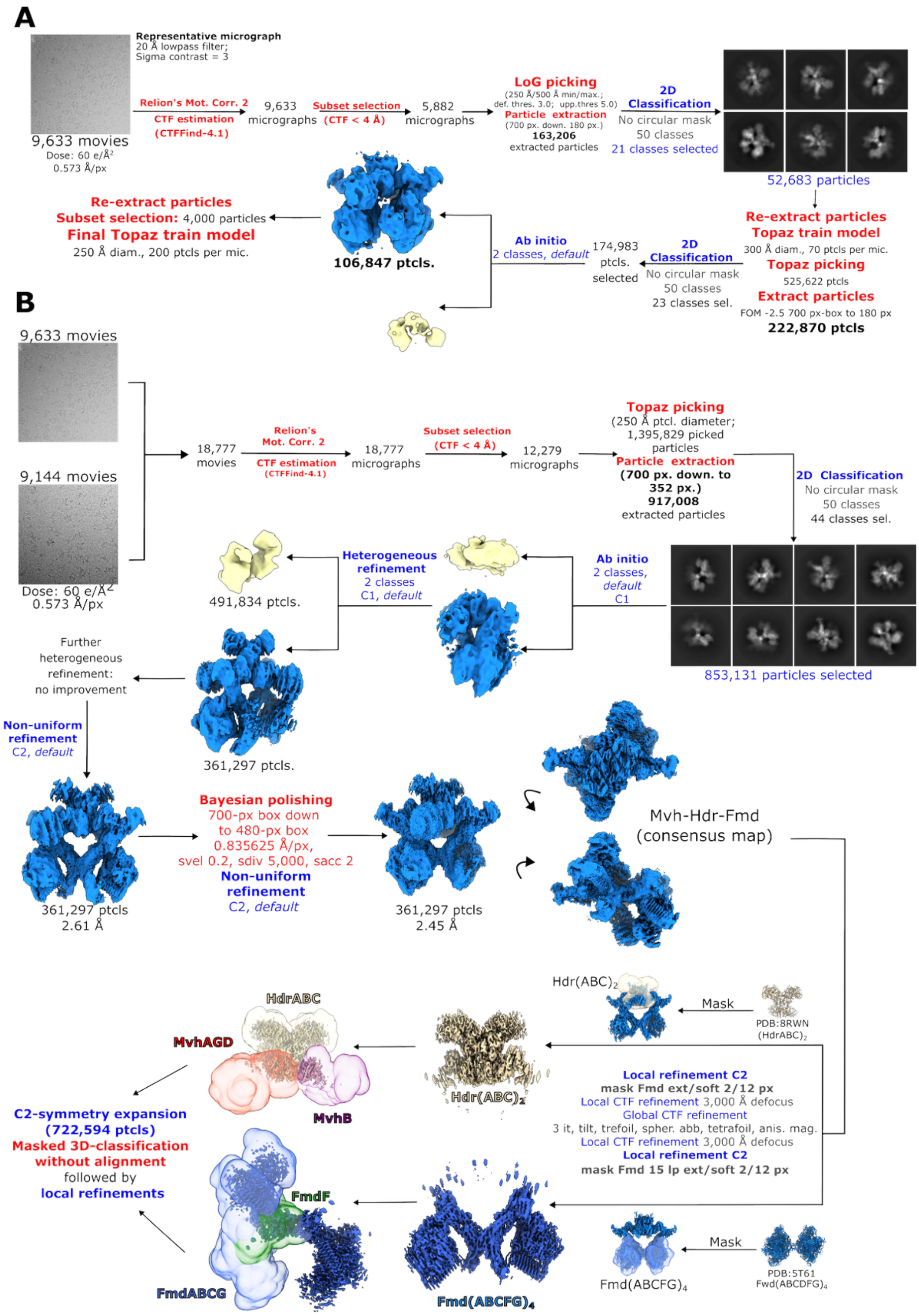


**Fig. S2. Initial steps of the cryo-EM processing workflow followed for the Mvh-Hdr-Fmd complex.** (**A**), Preliminary analysis of the first dataset to obtain a Topaz model for particle picking. (**B**), Initial data processing steps performed before C2-symmetry expansion and focused refinement of the particles. Processes run in RELION are colored red, while processes run in cryoSPARC are colored blue. Abbreviations used: External (ext.), pixel (px), per (p.), low-pass filter (lp.), particles (ptcls), extension (ext), soft-edge (soft).

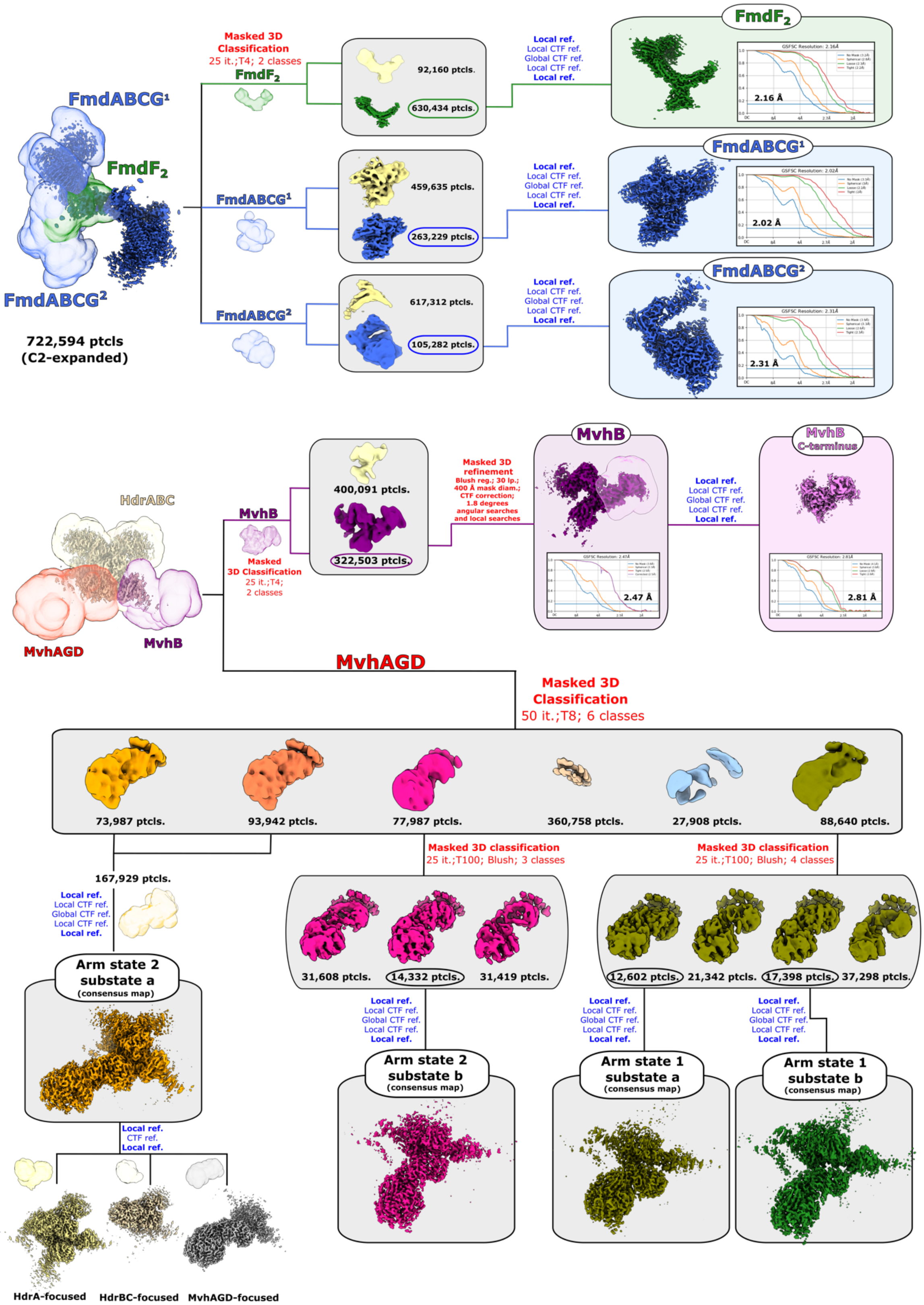


**Fig. S3. Further processing steps used to obtain focused maps of different regions of the full complex.** Processes run in RELION are colored red, while processes run in cryoSPARC are colored blue. Abbreviations used: Blush regularisation algorithm (Blush reg.), iterations (it.), refinement (ref.), diameter (diam.), pixel (px), low-pass filter (lp.), particles (ptcls).

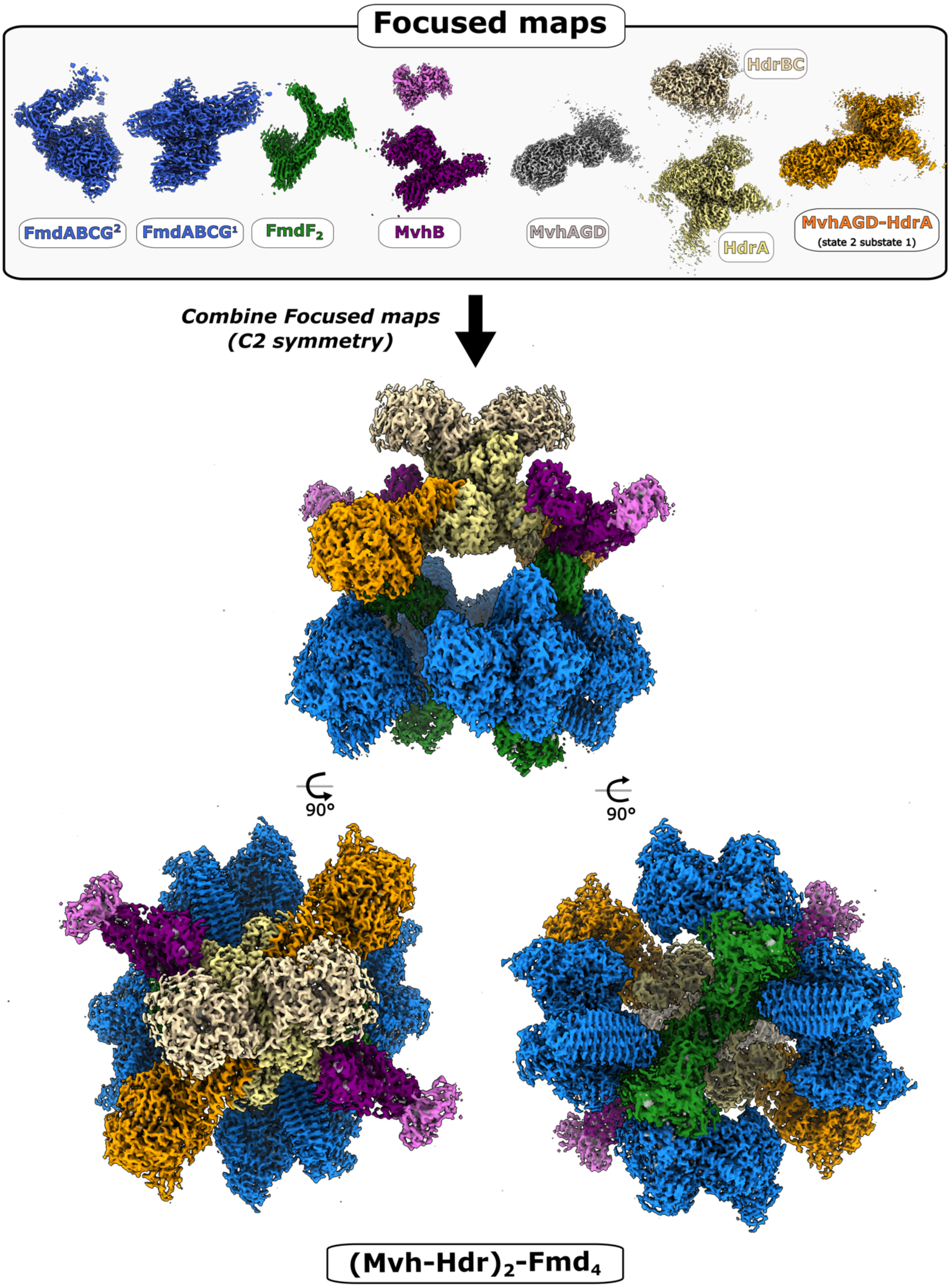


**Fig. S4.** The Phenix tool *combine focused maps(71)* was used for the generation of the C2-symmetric composite map of the Mvh-Hdr-Fmd complex with (Mvh-Hdr)$_2$-Fmd$_4$ stoichiometry.

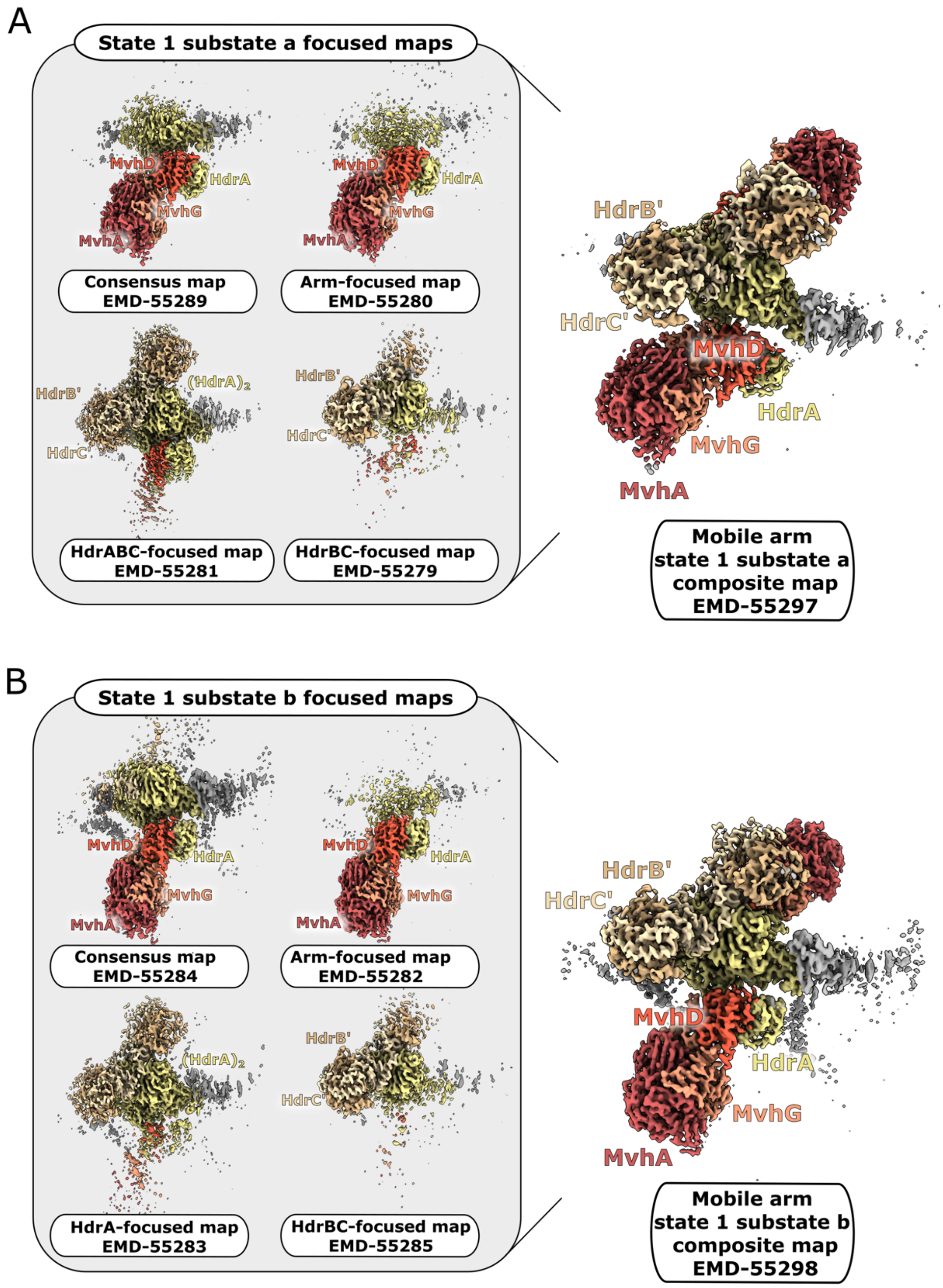


**Fig. S5. The Phenix tool *combine focused maps(71)* was used for the generation of the C2-symmetric composite maps of the Mvh-Hdr mobile arm in state 1, substates a (A) and b (B).**

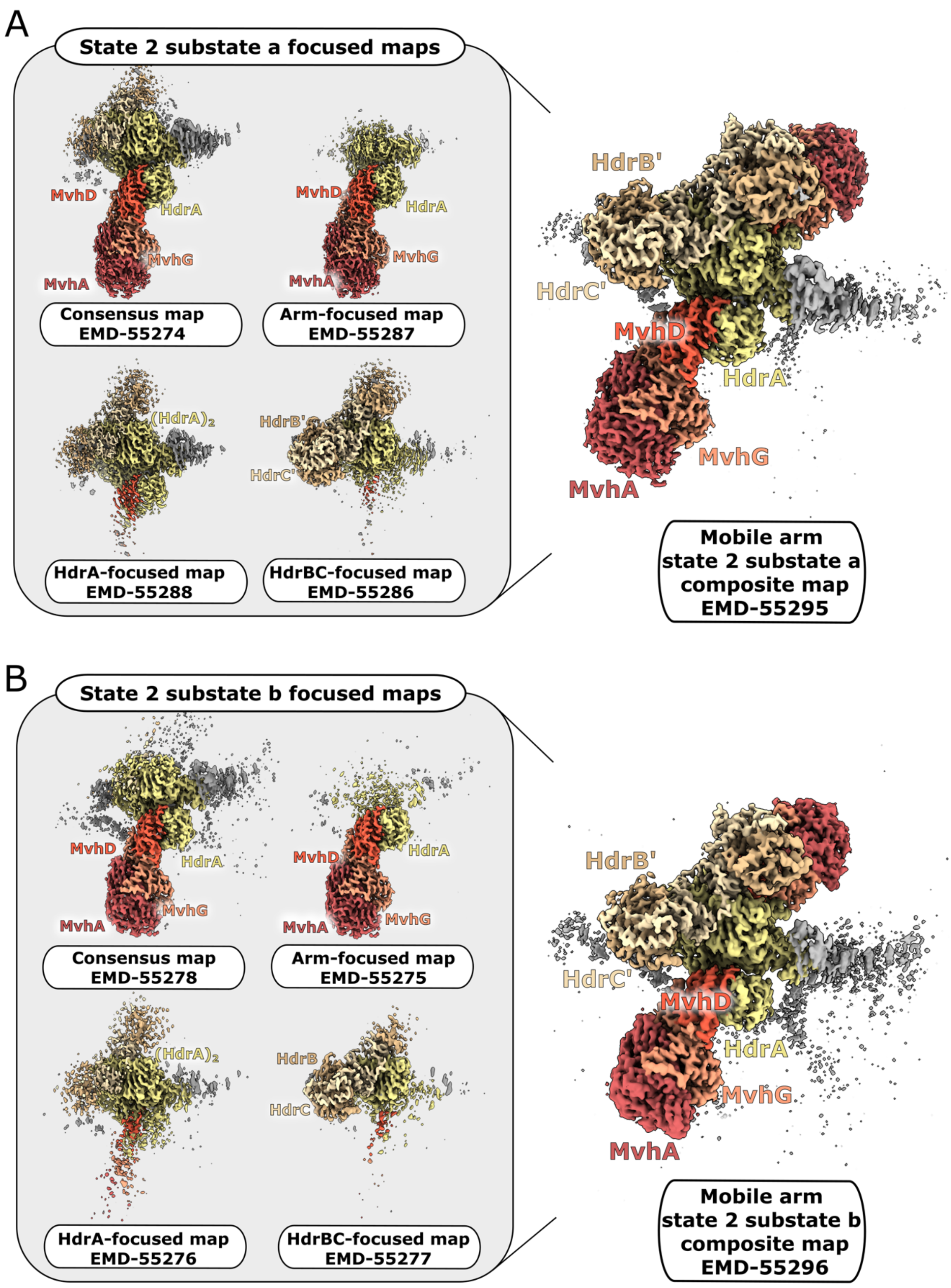


**Fig. S6. The Phenix tool *combine focused maps(71)* was used for the generation of the Mvh-Hdr mobile arm in state 2 substates a (A) and b (B).**

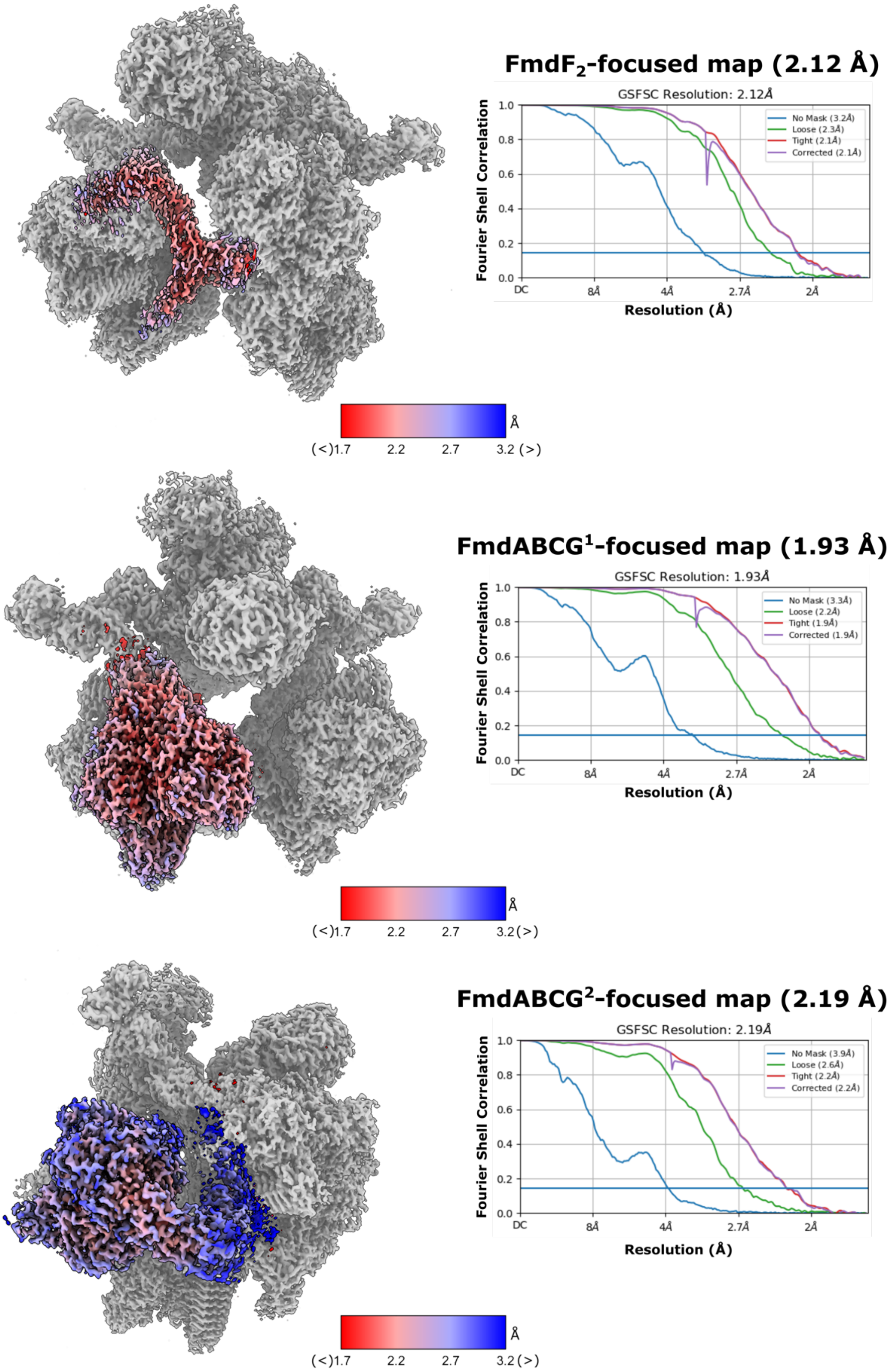


**Fig. S7. Local resolution and FSC curves of the Fmd-focused maps. Left:** Postprocessed maps of the Fmd reconstructions colored by local resolution**. Right:** Gold-standard Fourier Shell Correlation (FSC) curves of the 3D reconstructions indicating the resolution at FSC=0.143.

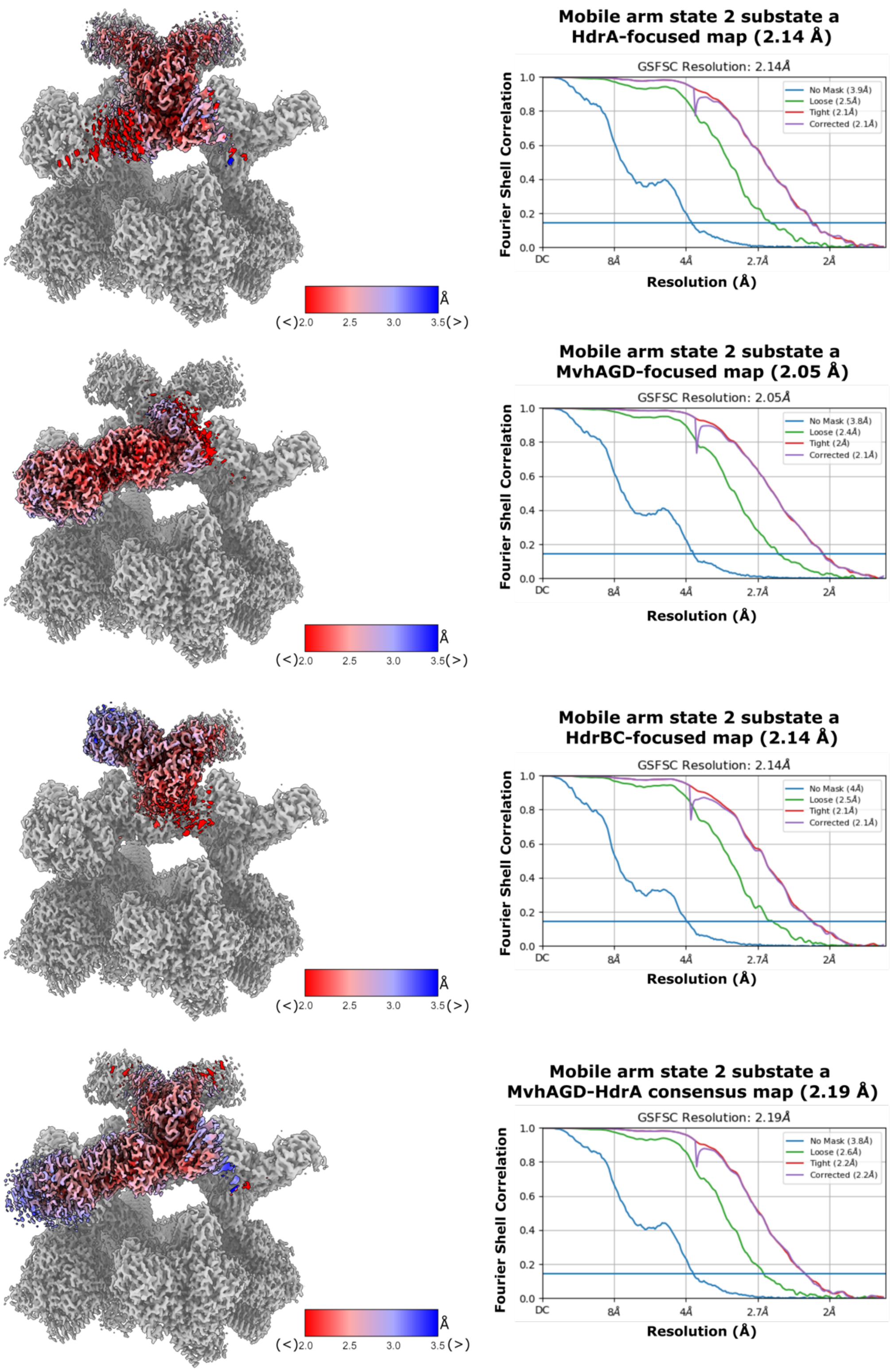


**Fig. S8. Local resolution and FSC curves of the mobile-arm-focused maps in state 2 substate a. Left:** Postprocessed maps of the reconstructions colored by local resolution**. Right:** Gold-standard Fourier Shell Correlation (FSC) curves of the 3D reconstructions indicating the resolution at FSC=0.143.

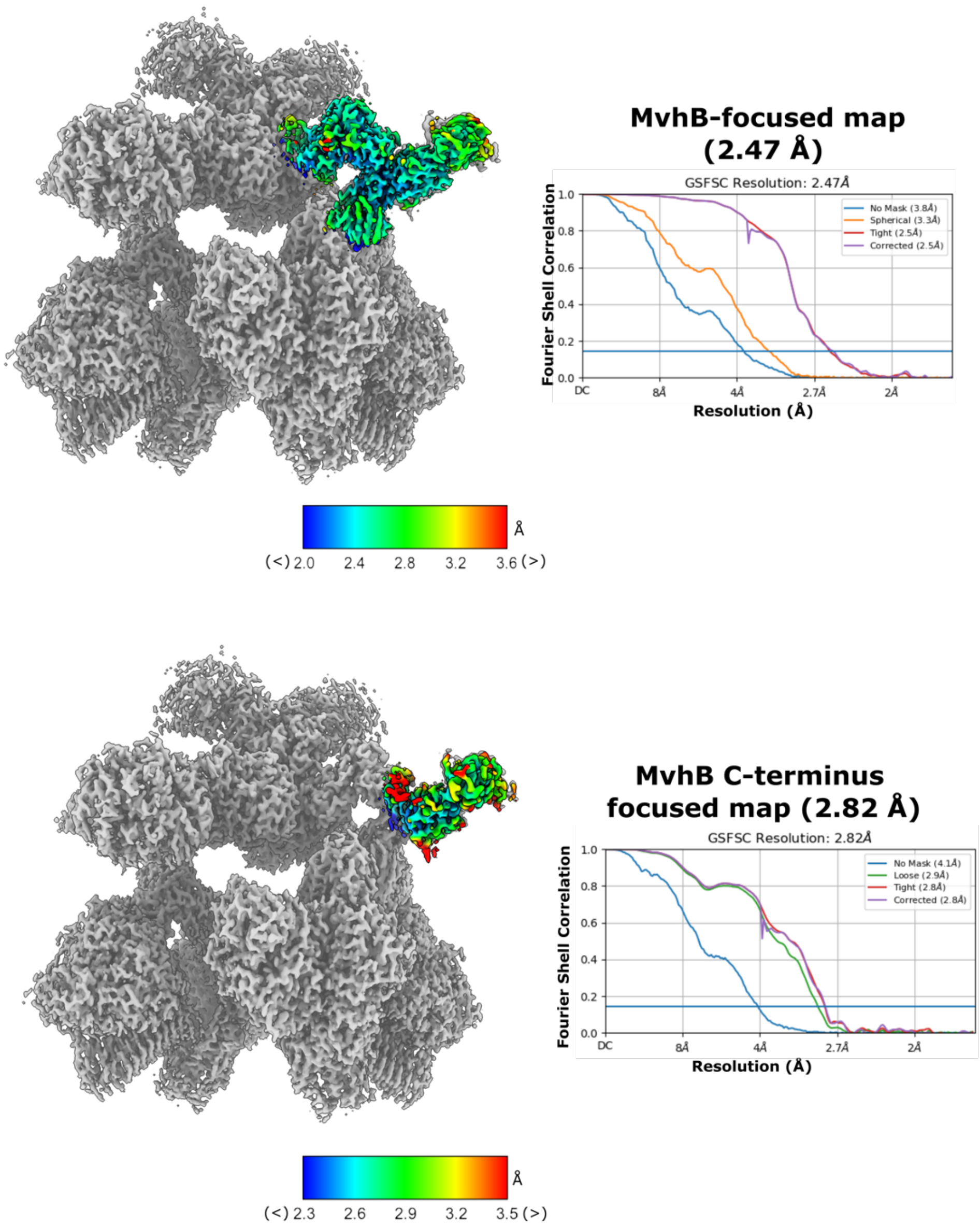


**Fig. S9. Local resolution and FSC curves of the MvhB-focused maps. Left:** Postprocessed maps of the reconstructions colored by local resolution**. Right:** Gold-standard Fourier Shell Correlation (FSC) curves of the 3D reconstructions indicating the resolution at FSC=0.143.

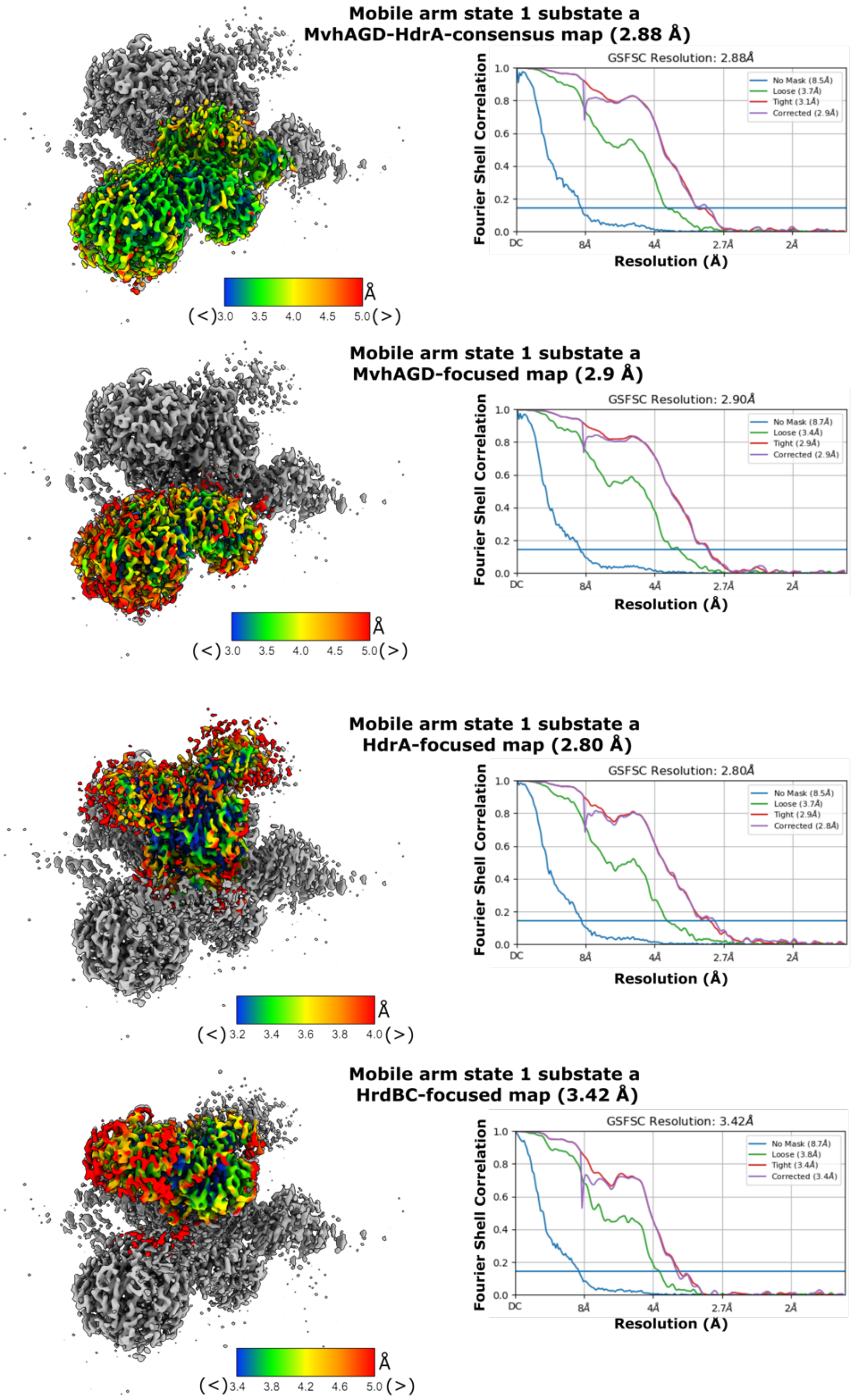


**Fig. S10. Local resolution and FSC curves of the mobile-arm-focused maps in state 1, substate a. Left:** Postprocessed maps of the reconstructions colored by local resolution**. Right:** Gold-standard Fourier Shell Correlation (FSC) curves of the 3D reconstructions indicating the resolution at FSC=0.143.

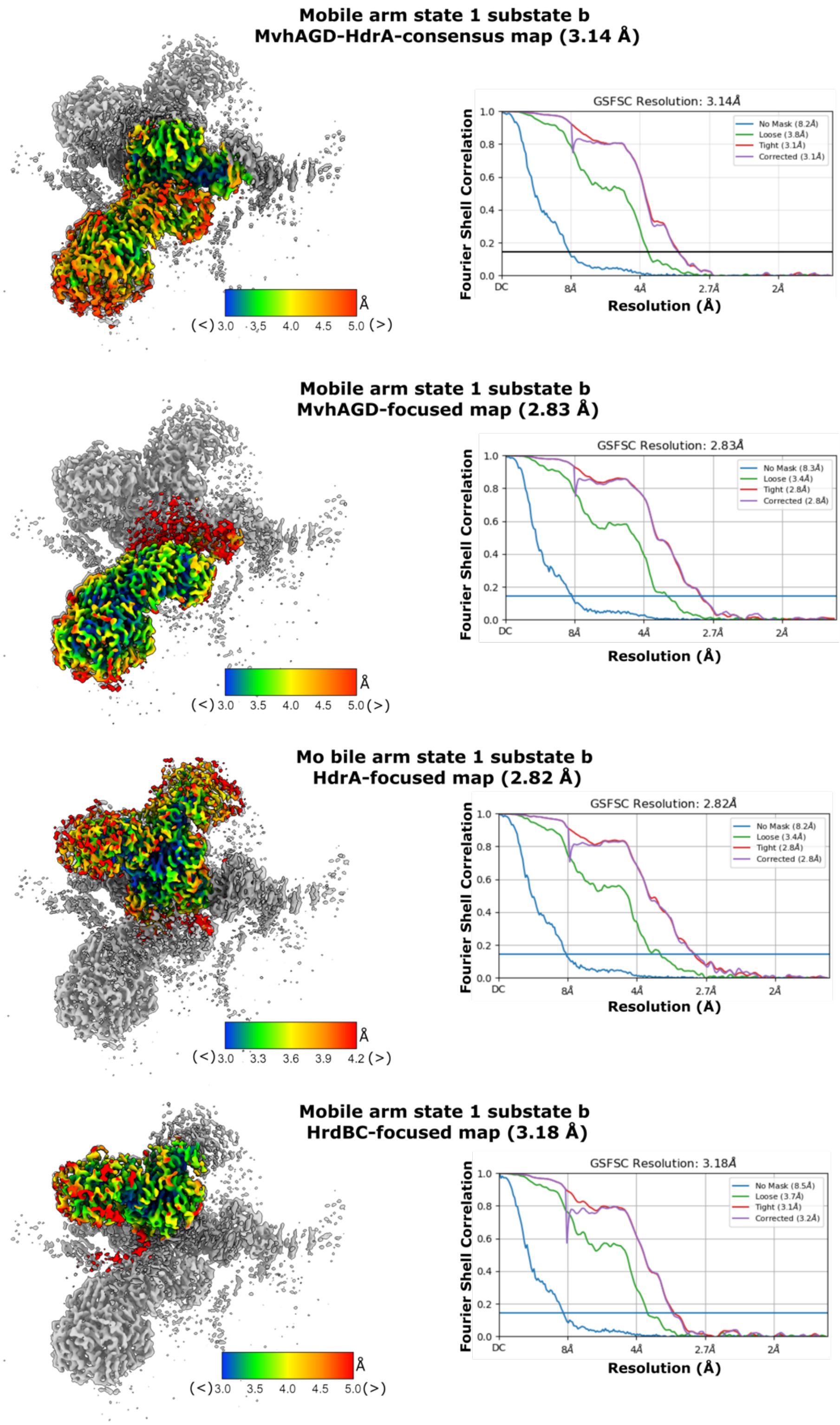


**Fig. S11. Local resolution and FSC curves of the mobile-arm-focused maps in state 1, substate b. Left:** Postprocessed maps of the reconstructions colored by local resolution**. Right:** Gold-standard Fourier Shell Correlation (FSC) curves of the 3D reconstructions indicating the resolution at FSC=0.143.

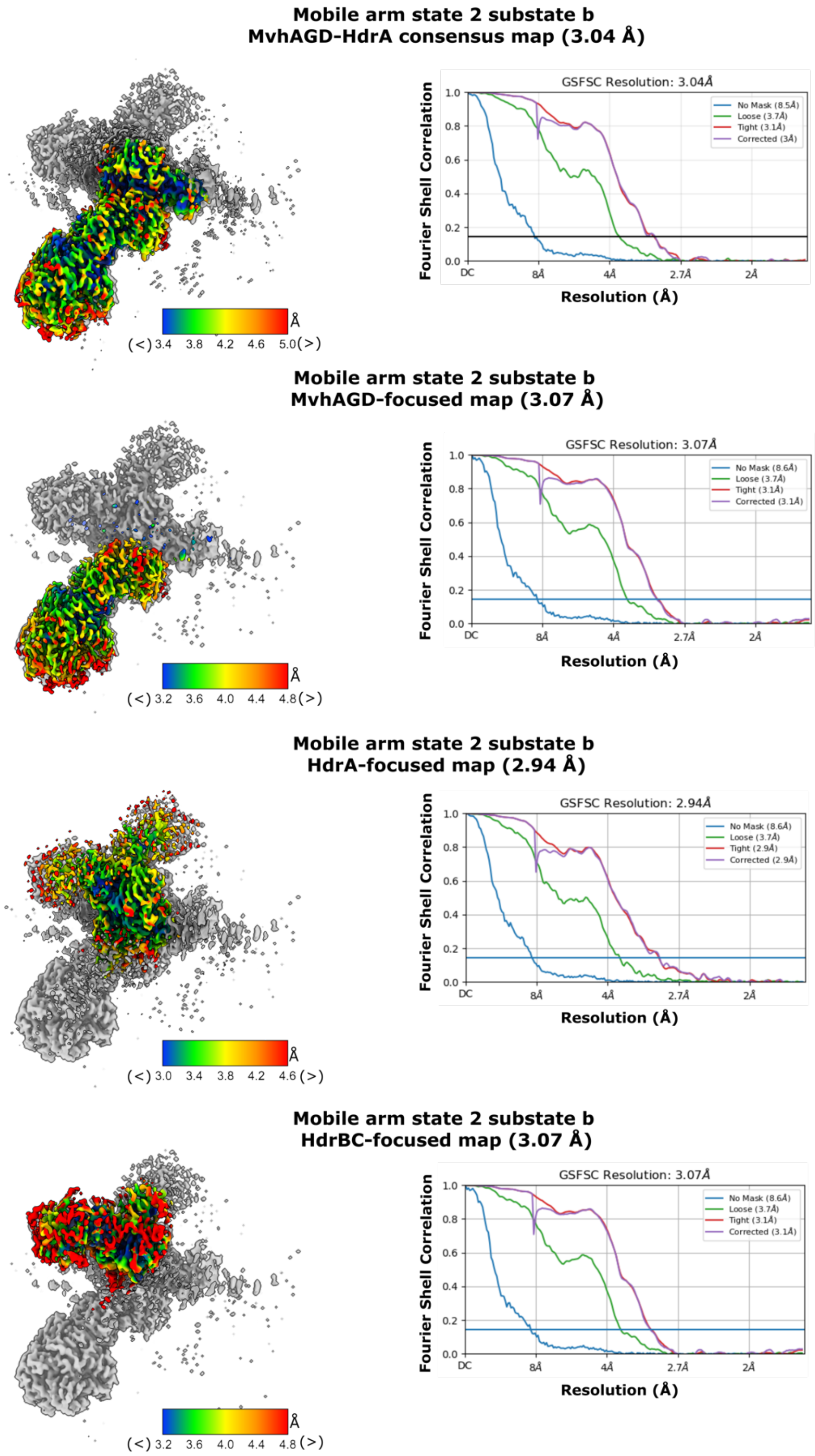


**Fig. S12. Local resolution and FSC curves of the mobile-arm-focused maps in state 2, substate b. Left:** Postprocessed maps of the reconstructions colored by local resolution**. Right:** Gold-standard Fourier Shell Correlation (FSC) curves of the 3D reconstructions indicating the resolution at FSC=0.143.

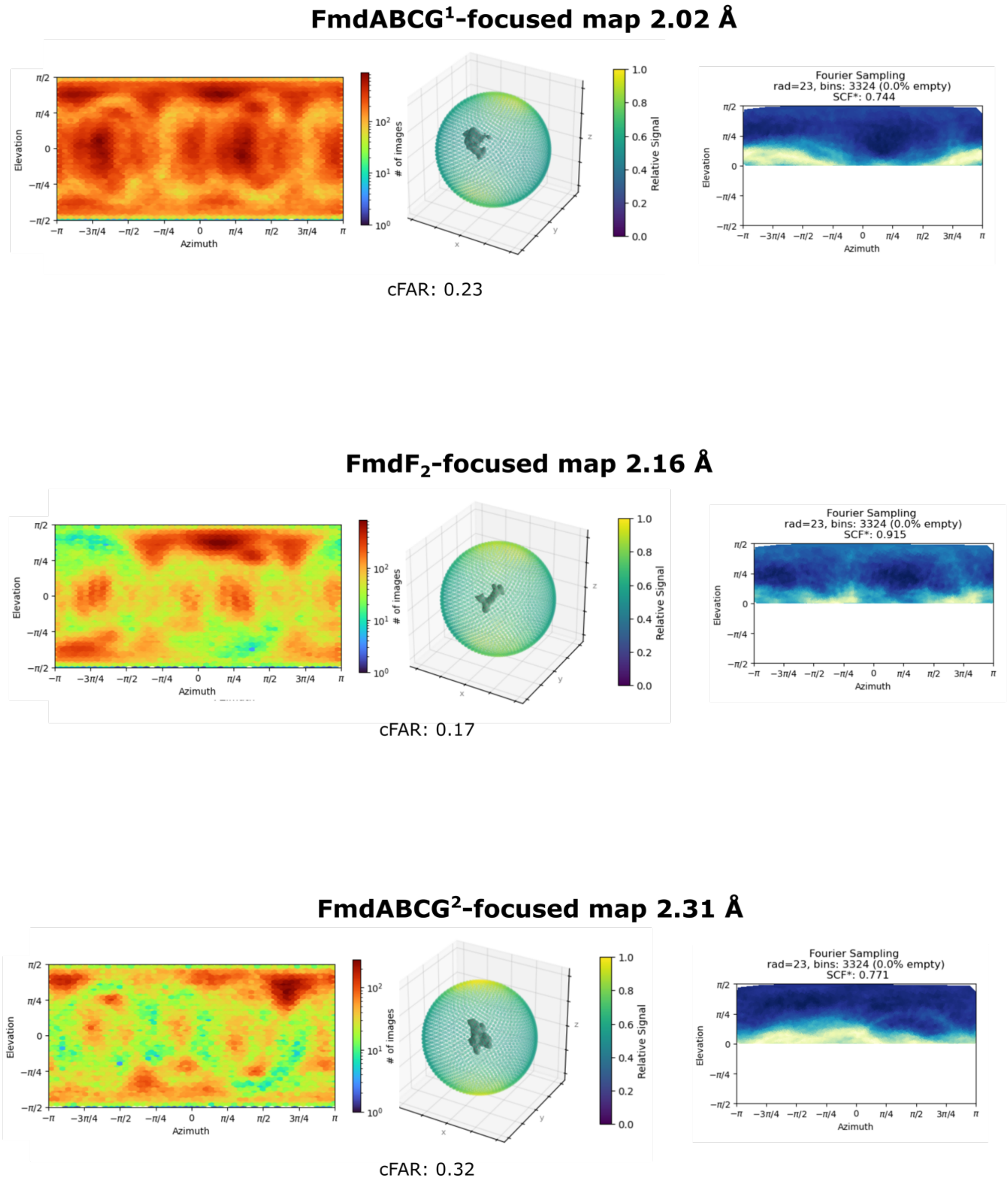


**Fig. S13. Orientation distributions of the Fmd-focused cryo-EM maps.** From left to right, viewing direction distribution plot, relative signal visualized in a 3D coloured scatter plot with a low-pass-filtered volume fit inside, and Fourier sampling. The conical FSC area ratio (cFAR) and the Sampling Compensation Factor (SCF) values are indicated for all maps.

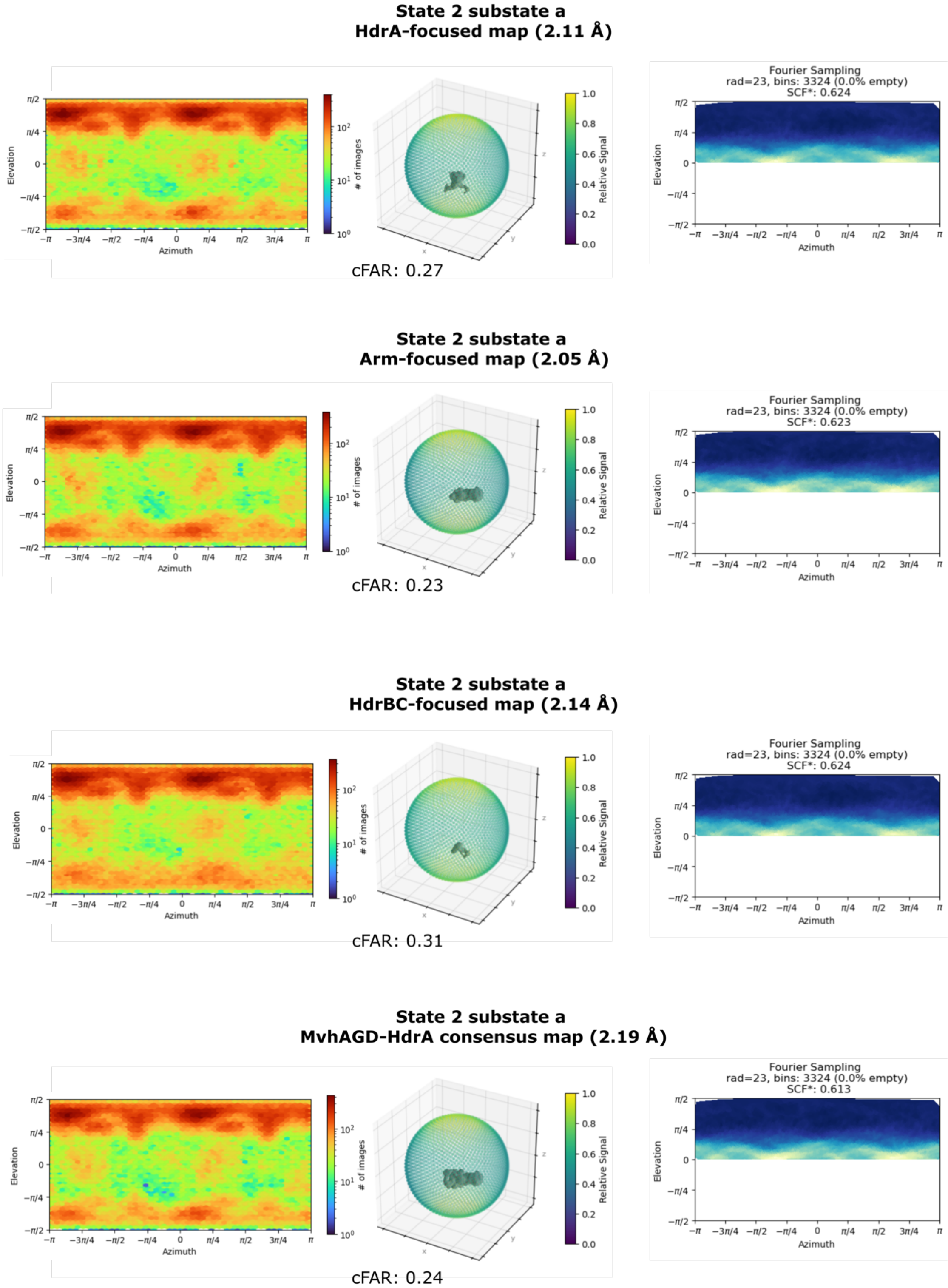


**Fig. S14. Orientation distributions of the cryo-EM maps for the mobile arm in state 2, substate a. From left to right**, viewing direction distribution plot, relative signal visualized in a 3D-coloured scatter plot with a low-pass-filtered volume fit inside, and Fourier sampling. The conical FSC area ratio (cFAR) and the Sampling Compensation Factor (SCF) values are indicated for all maps.

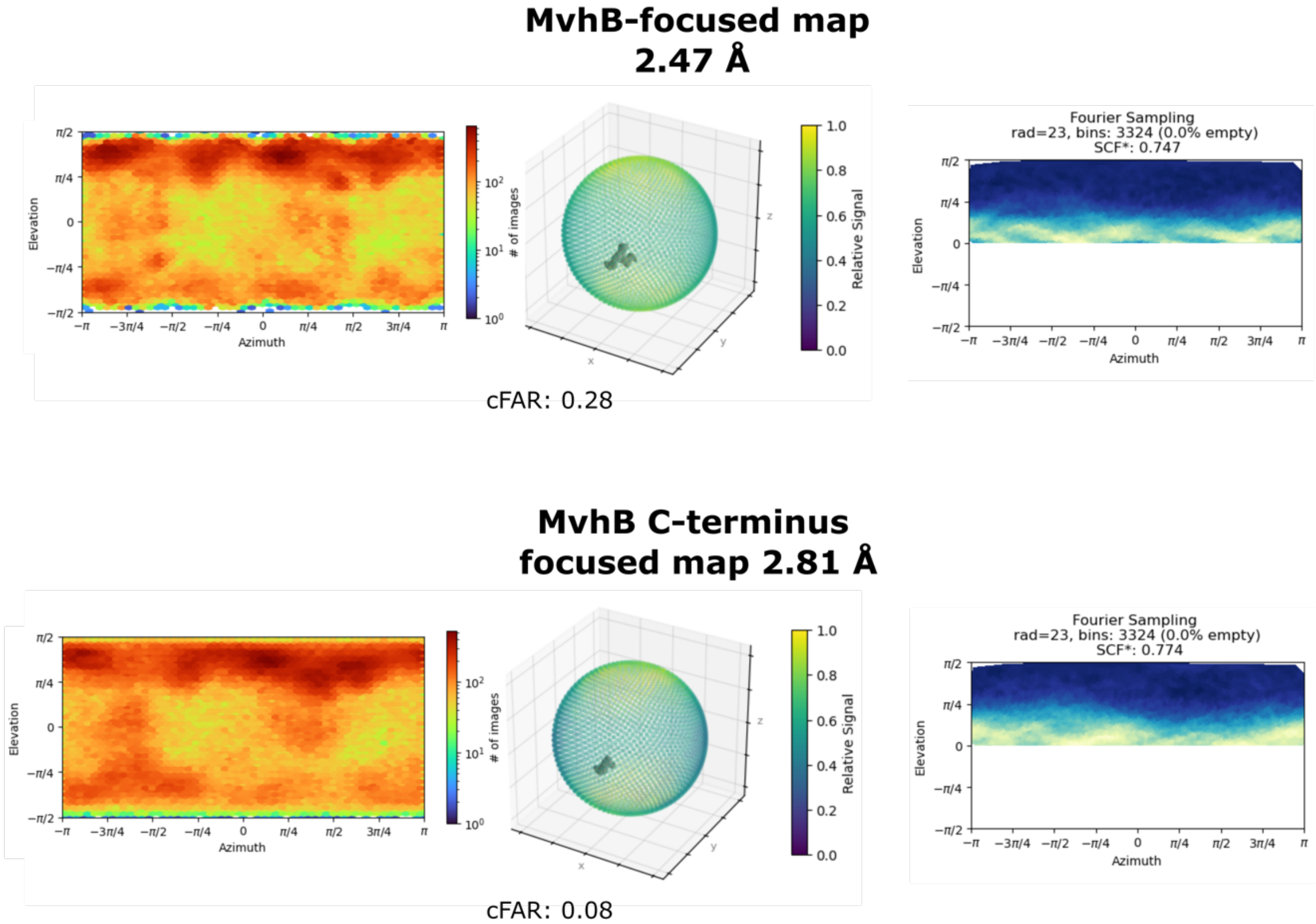


**Fig. S15. Orientation distributions of the MvhB-focused cryo-EM maps.** From left to right, viewing direction distribution plot, relative signal visualized in a 3D-coloured scatter plot with a low-pass-filtered volume fit inside, and Fourier sampling. The conical FSC area ratio (cFAR) and the Sampling Compensation Factor (SCF) values are indicated for all maps.

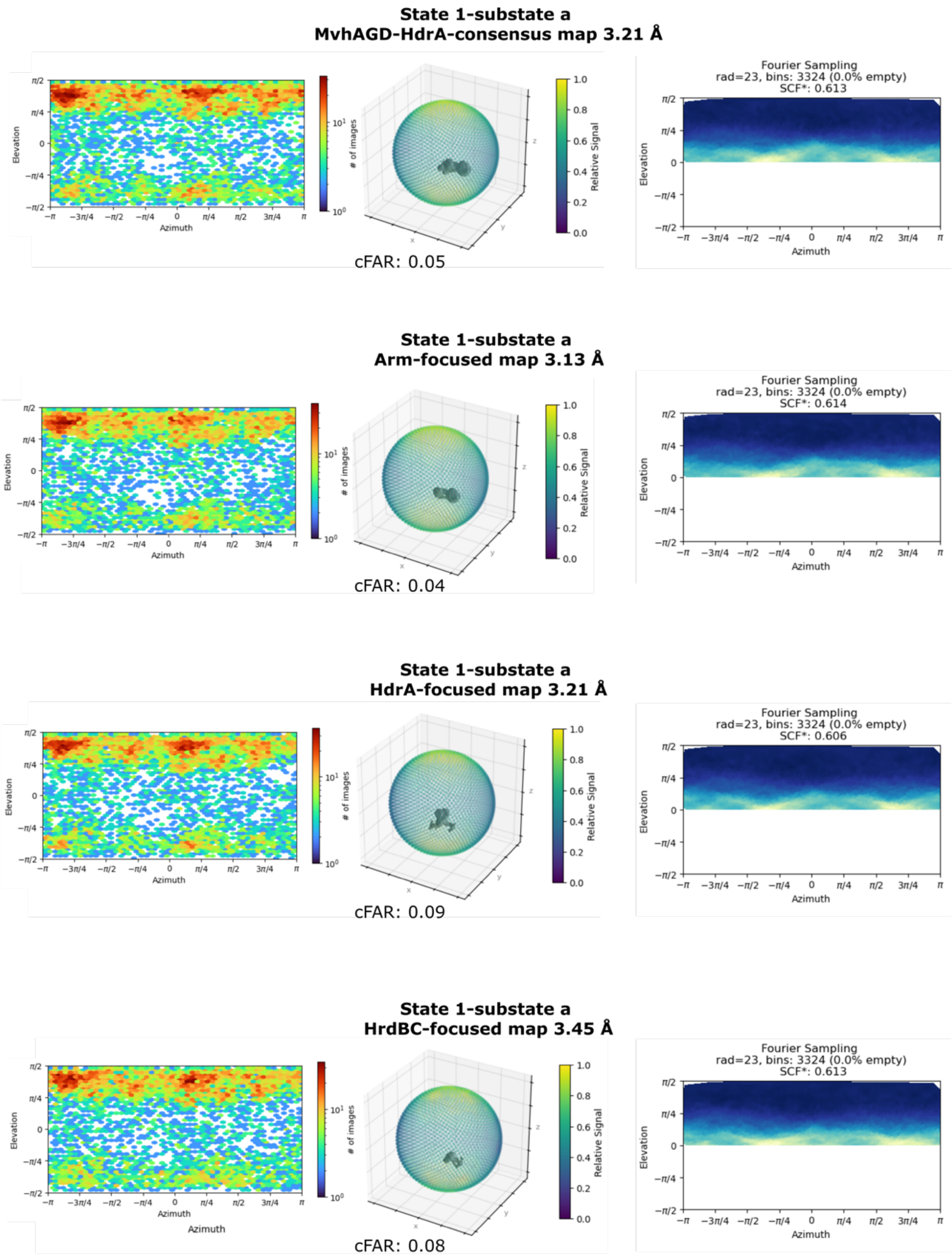


**Fig. S16. Orientation distributions of cryo-EM maps for the mobile arm in state 1, substate a.** From left to right, viewing direction distribution plot, relative signal visualized in a 3D coloured scatter plot with a low-pass-filtered volume fit inside, and Fourier sampling. The conical FSC area ratio (cFAR) and the Sampling Compensation Factor (SCF) values are indicated for all maps.

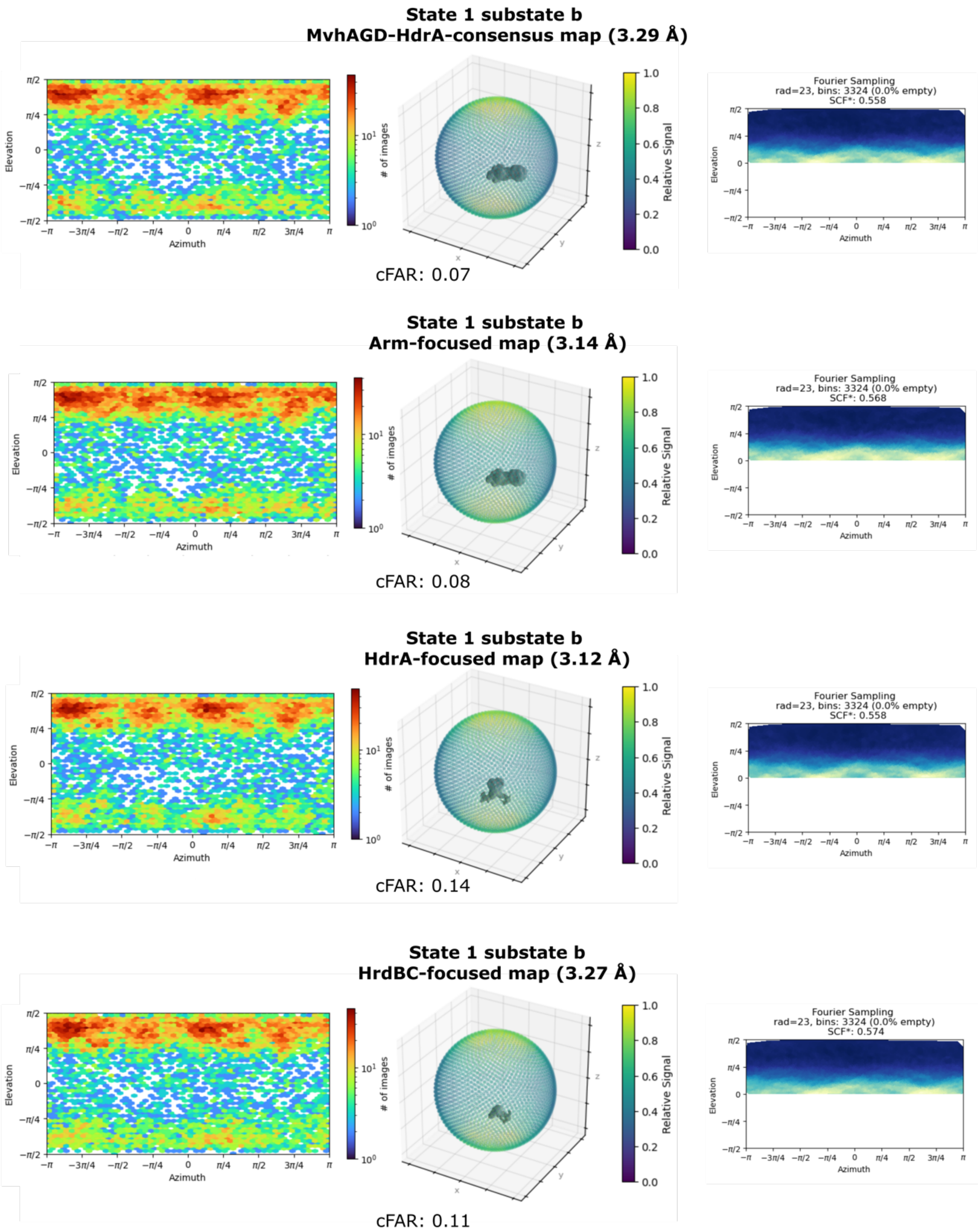


**Fig. S17. Orientation distributions of cryo-EM maps for the mobile arm in state 1, substate b.** From left to right, viewing direction distribution plot, relative signal visualized in a 3D coloured scatter plot with a low-pass-filtered volume fit inside, and Fourier sampling. The conical FSC area ratio (cFAR) and the Sampling Compensation Factor (SCF) values are indicated for all maps.

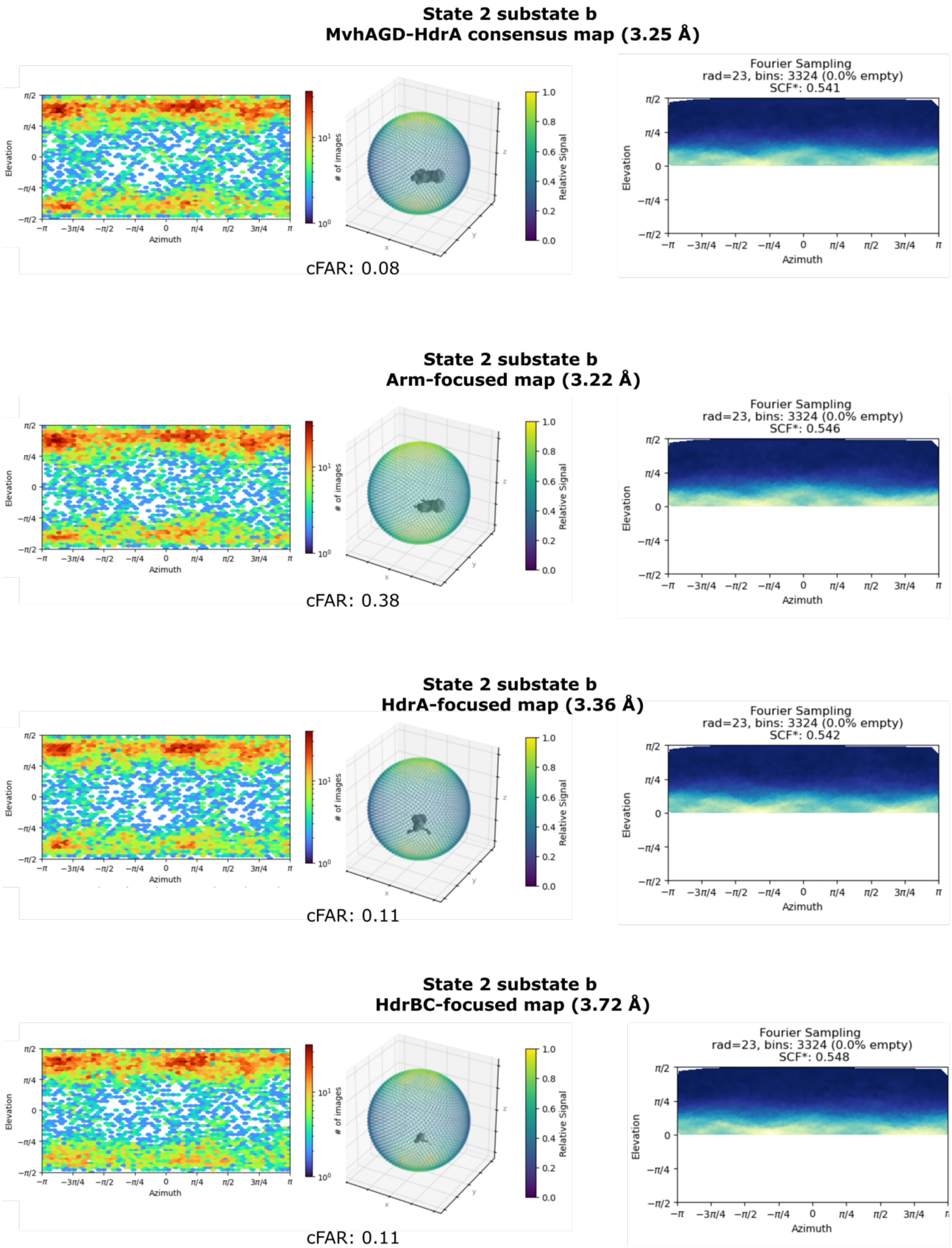


**Fig. S18. Orientation distributions of cryo-EM maps for the mobile arm in state 2, substate b.** From left to right, viewing direction distribution plot, relative signal visualized in a 3D coloured scatter plot with a low-pass-filtered volume fit inside, and Fourier sampling. The conical FSC area ratio (cFAR) and the Sampling Compensation Factor (SCF) values are indicated for all maps.

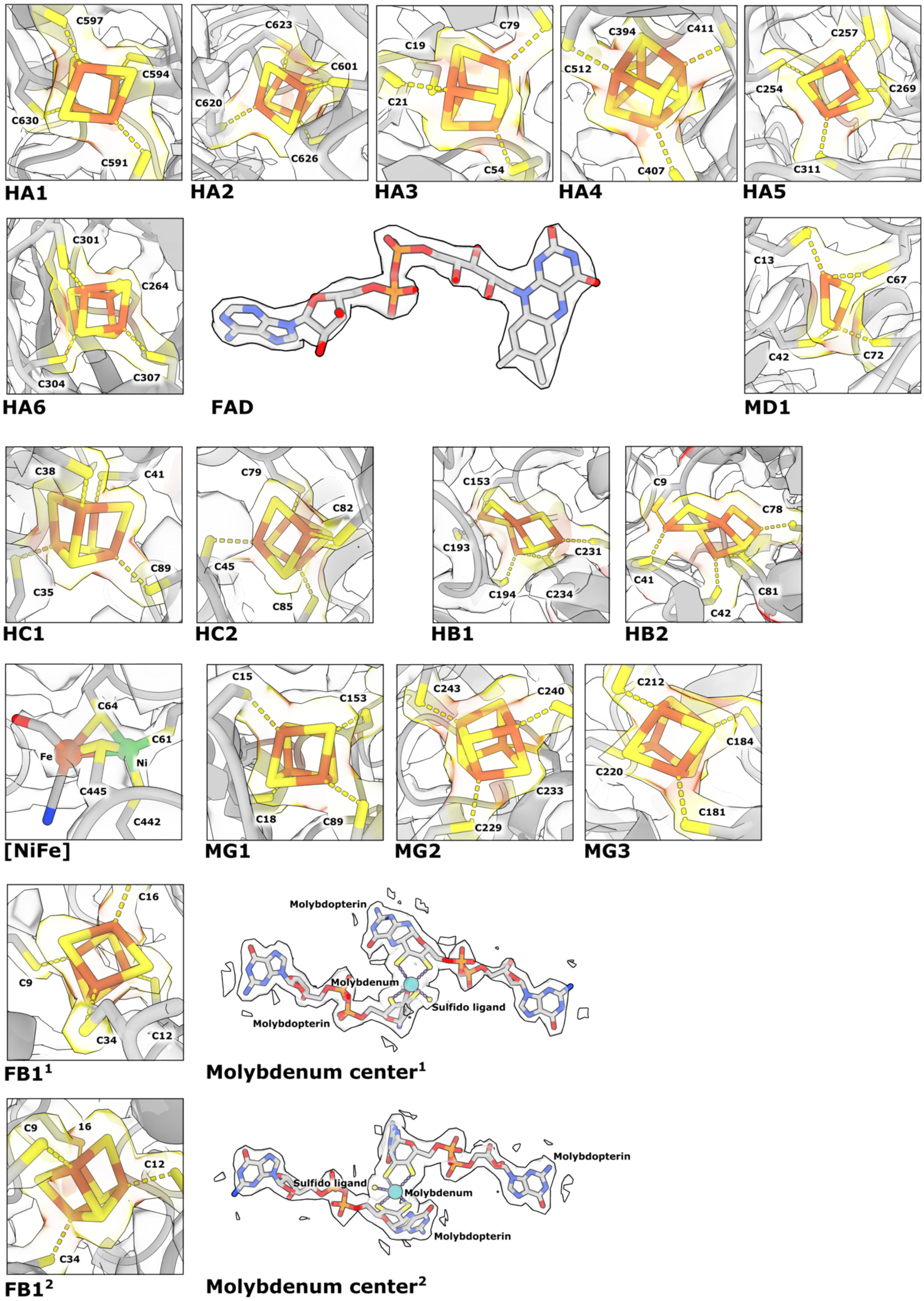


**Fig. S19. Map densities for all the cofactors present in the Mvh-Hdr-Fmd composite map**. Front and back clipping planes were introduced for better visualization.

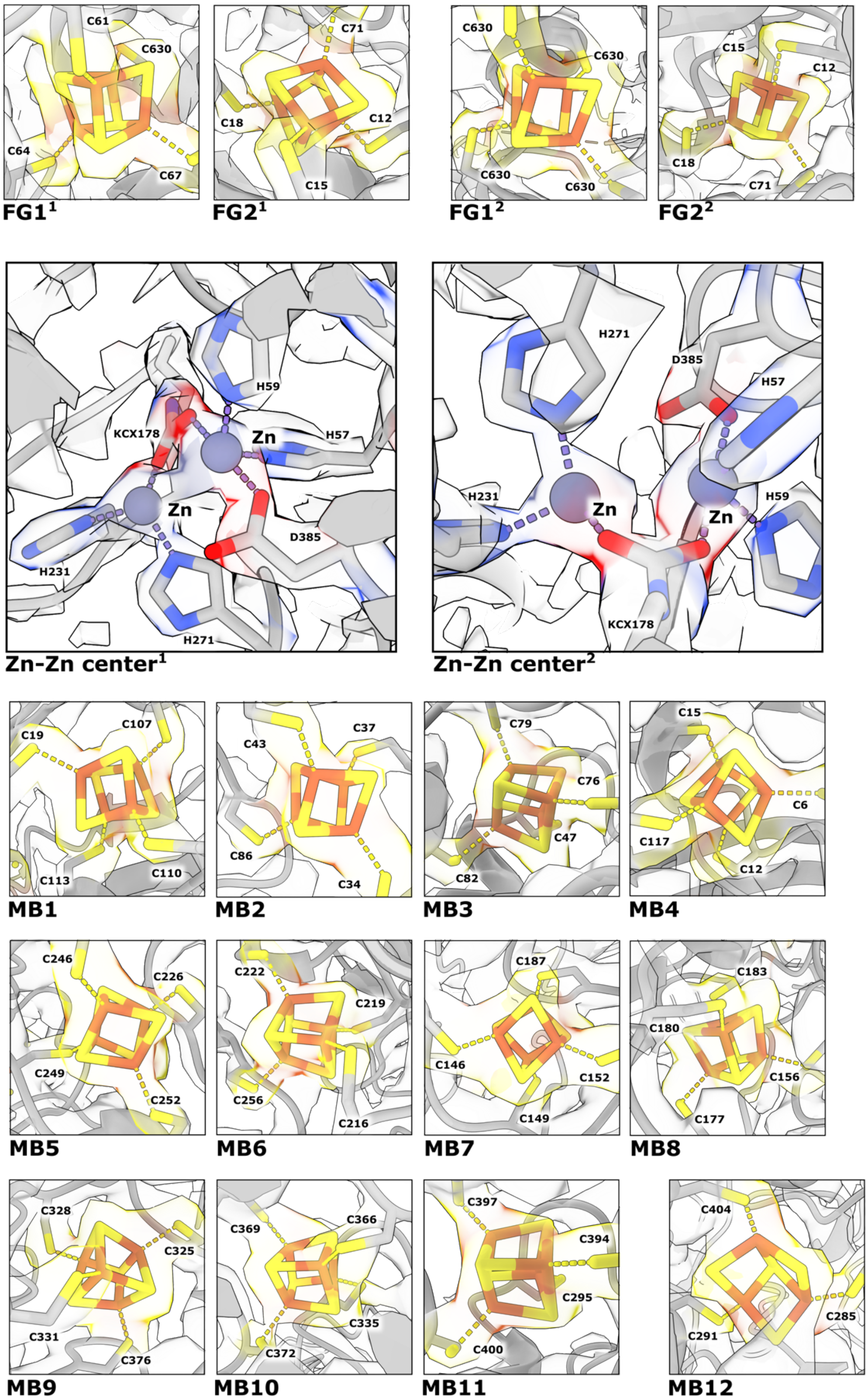


**Fig. S20. Map densities for all the cofactors present in the Mvh-Hdr-Fmd composite map**.
Front and back clipping planes were introduced for better visualization.

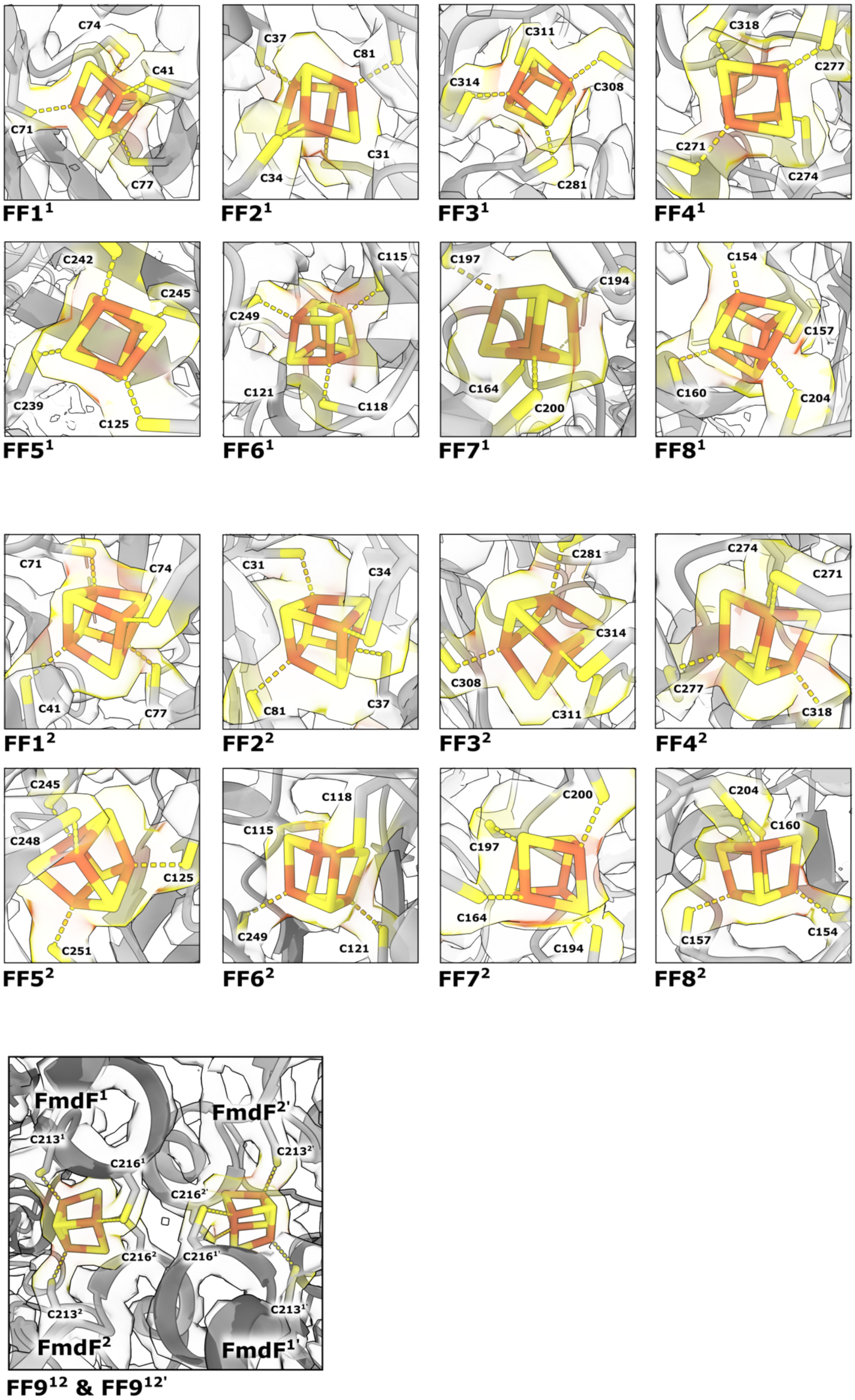


**Fig. S21. Map densities for all the cofactors present in the Mvh-Hdr-Fmd composite map**. Front and back clipping planes were introduced for better visualization.

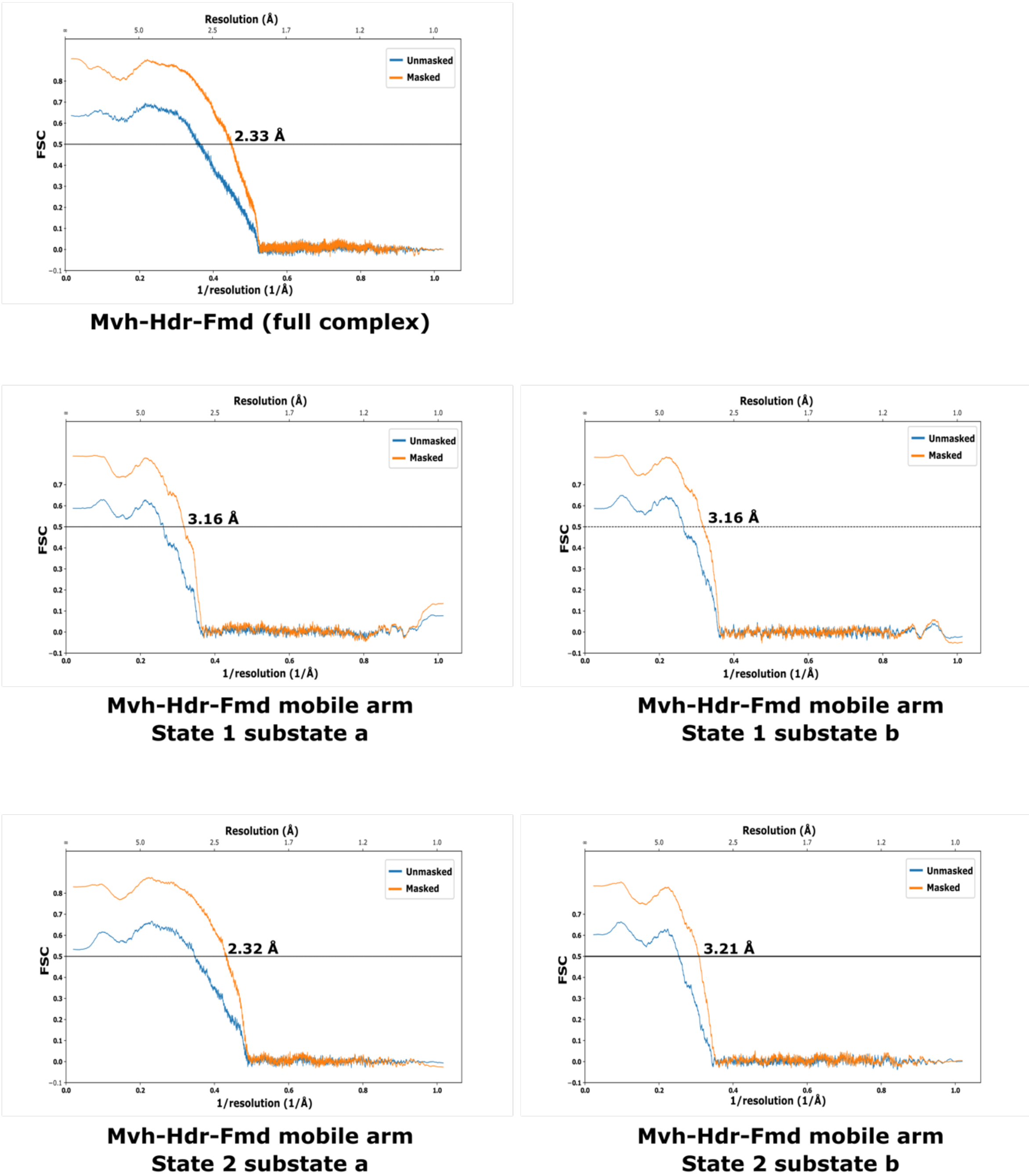


**Fig. S22. Model-map FSC curves.** FSC between refined atomic models and corresponding maps are plotted as calculated in Phenix(*47*). The reported resolutions were determined using correlations at 0.5 threshold, indicated by black horizontal lines.

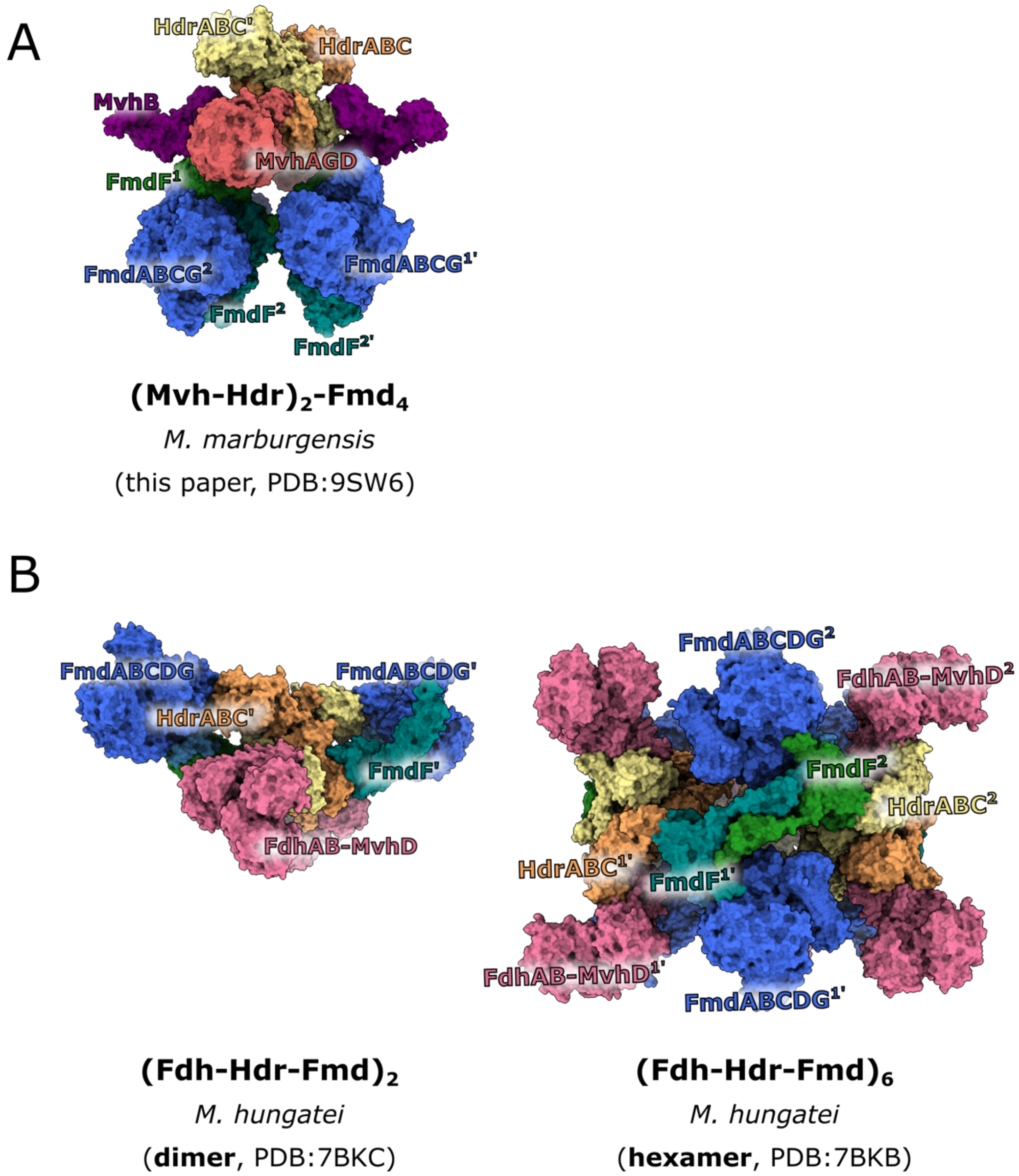


**Fig. S23. The supercomplex structures of Mvh-Hdr-Fmd and Fdh-Hdr-Fmd complexes.** The Mvh-Hdr-Fmd complex of *M. marburgensis* (**A**) displays different symmetry and stoichiometry from the *M. hungatei* Fdh-Hdr-Fmd complex (**B**). In **B**, the dimeric (Fdh-Hdr-Fmd)$_2$ (left) and hexameric (Fdh-Hdr-Fmd)$_6$ (right) forms are shown, where the dimeric form believed to result from partial disassembly of the hexameric form.

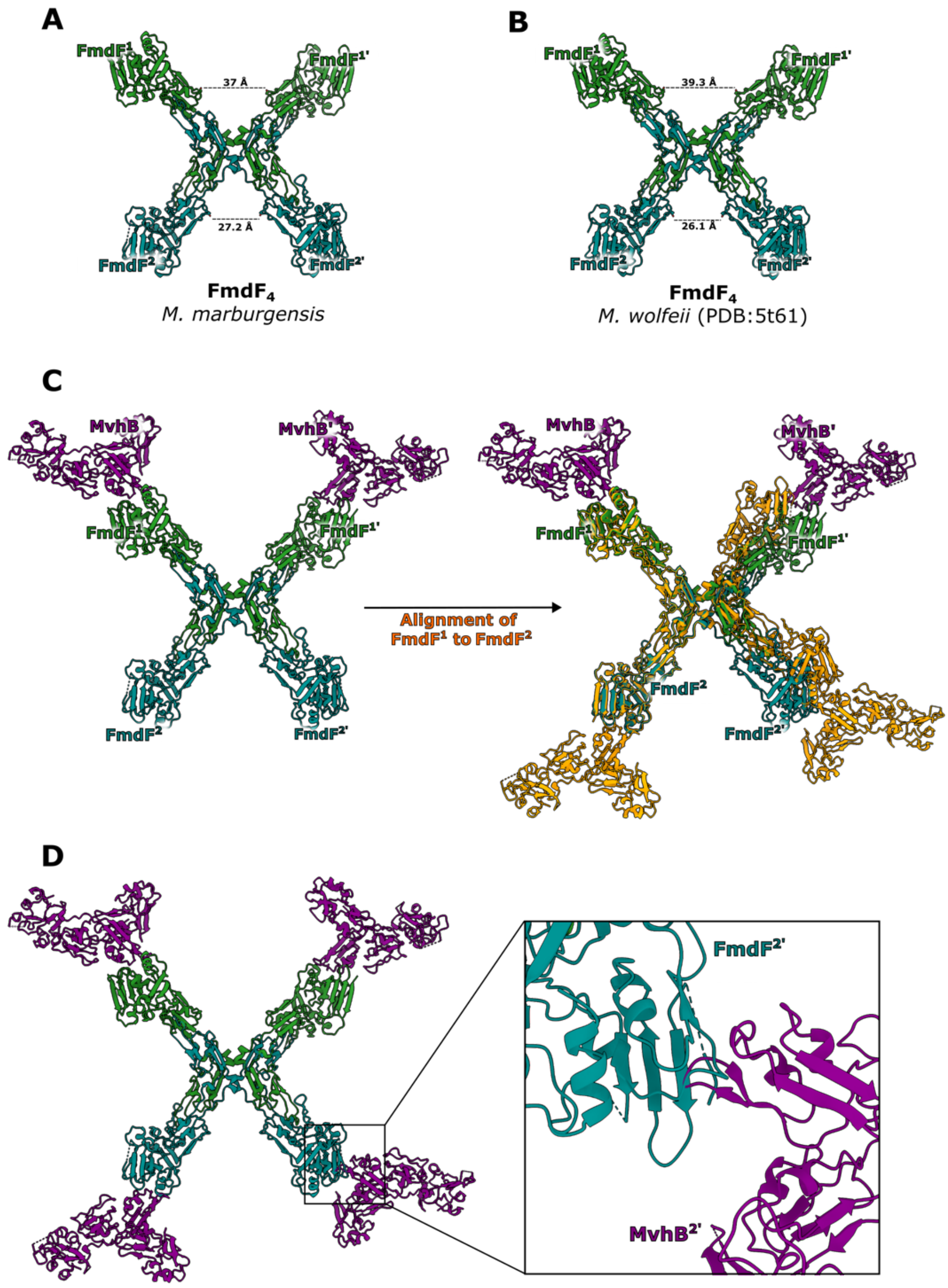


**Fig. S24. Distortion of fourfold symmetry in the FmdF tetrameric assembly may hinder the formation of a complex with (Mvh-Hdr-Fmd)$_4$ stoichiometry.** The FmdF tetramer (Fmd$_4$) of *M. marburgensis* and *M. wolfeii* (PDB:5T61)(*8*) show a distorted D4 symmetry, which can be quantified by measuring the distance between Thr114 residues of the individual subunits (**A**, **B**). Interestingly, this distortion is also observed in a crystal structure of the Fmd tetramer from *M. wolfeii* in the absence of MvhB/Hdr (**B**), and in spite of the fact that all FmdF chains have the same sequence. As a consequence, rotating a second copy of the complex to structurally align FmdF$^2$ onto FmdF$^1$ (C) does not allow for functional interaction of Fmd$^{2'}$ with MvhB' (**C**,**D** with the callout shown in D showing a lack of functional interaction, and formation of clashes, with MvhB**).**

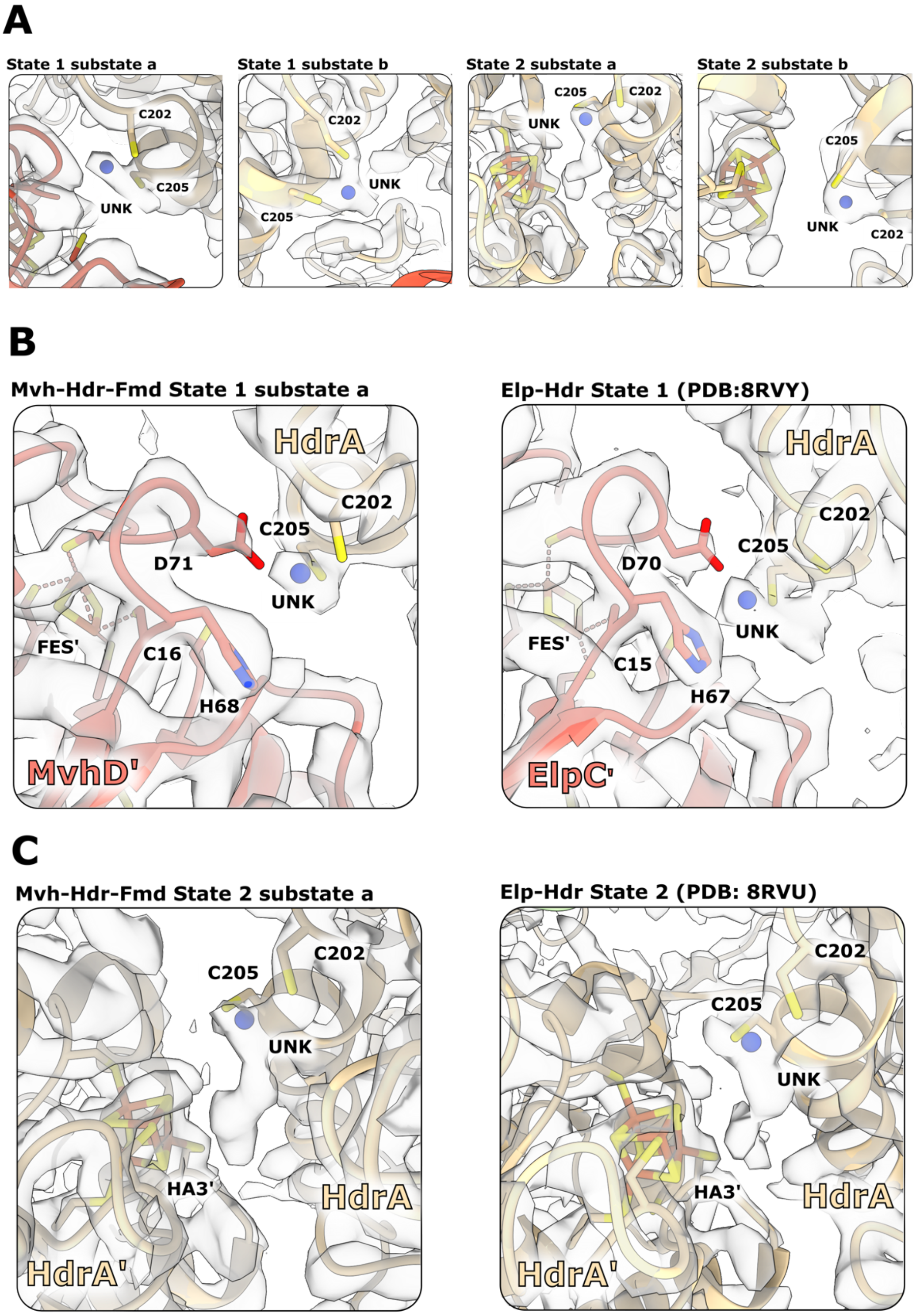


**Fig. S25 Conserved Cys residues of HdrA bind an unknown ligand (UNK).** The ligand is observed in all the different solved mobile arm states in Mvh-Hdr-Fmd from *M. marburgensis* (this study) (**A**). Comparison of the unknown ligand binding site between Mvh-Hdr-Fmd and Elp-Hdr from *M. marburgensis* (*17*) in the equivalent conformational states (**B** and **C**).

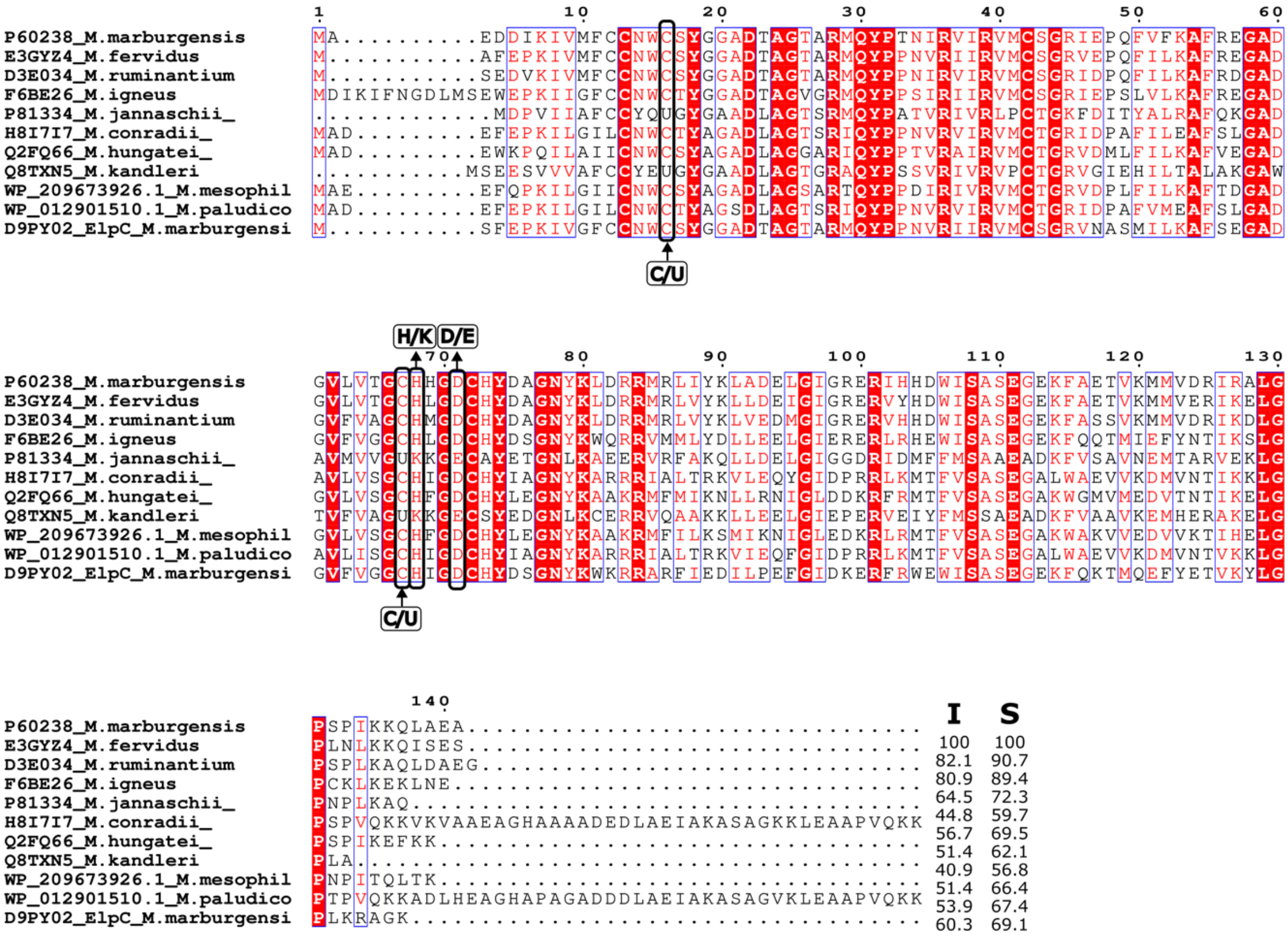


**Fig. S26. Sequence alignment of *M. marburgensis* MvhD with MvhD proteins of other representative species of class I and class II methanogens.** *Methanothermobacter marburgensis* (*M. marburgensis*), *Methanococcus igneus (M. igneus)*, *Methanothermus fervidus (M. fervidus), Methanocaldococcus jannaschii (M. jannaschii), Methanobrevibacter ruminantium* (*M. ruminantium*), *Methanopyrus kandleri* (*M. kandleri*), *Methanospirillum hungatei (M. hungatei)*, *Methanocella paludicola* (*M. paludicola*), *Methanocella conradii* (*M. conradii*), *Methanolinea mesophila* (*M. mesophila*). The residues at 68 and 71 (*M. marburgensis* numbering; highlighted with boxes) are involved in binding an unknown ligand (see fig. S25), in which conservative changes (H or K) at the position 68 and (D or K) at 71 are observed. Percent sequence identity (I) and similarity (S) between *M. marburgensis* MvhD and the other MvhD proteins are indicated.

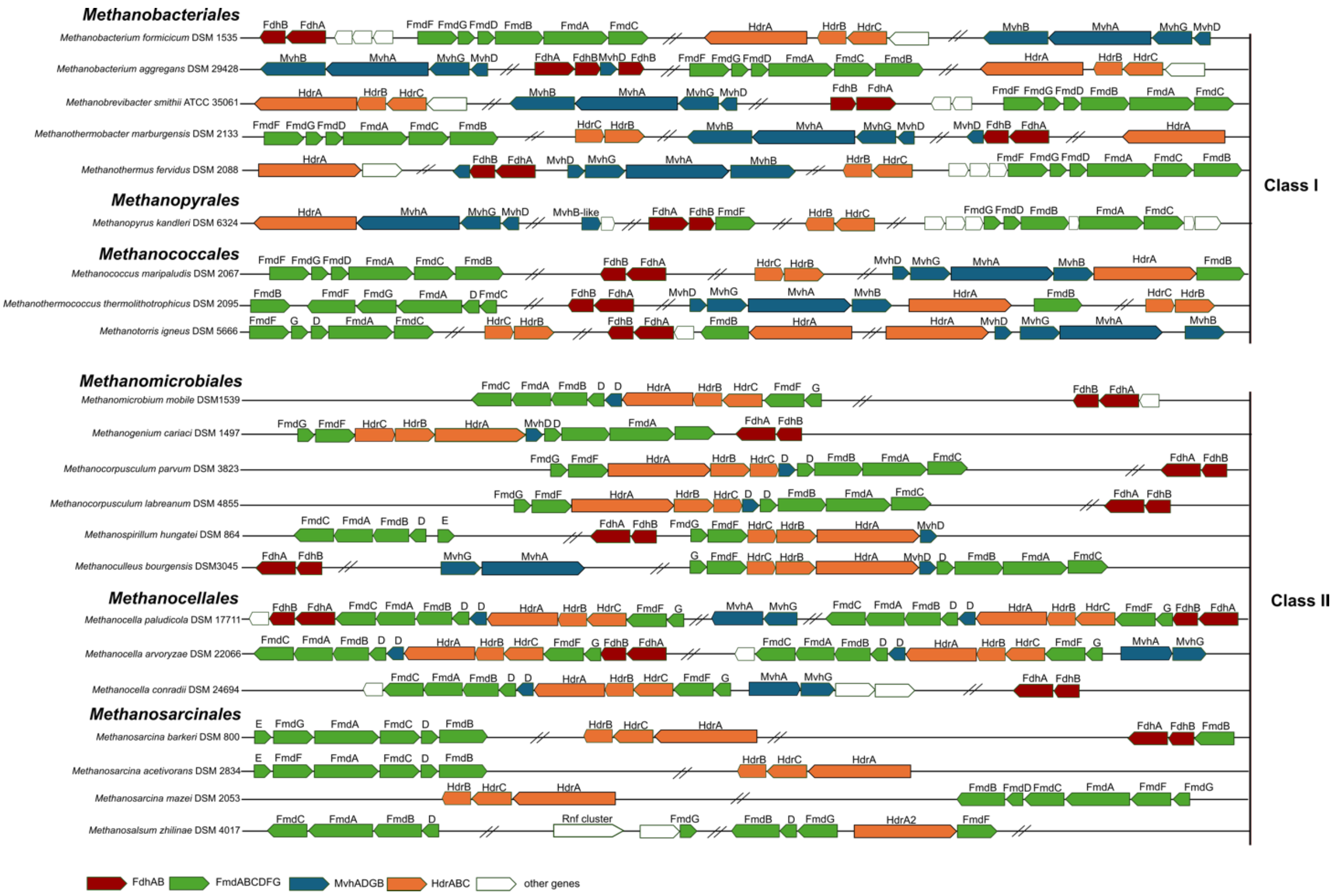


**Fig. S27. The genetic organization of Fmd, Hdr, Mvh, and Fdh genes among selected hydrogenotrophic methanogens from Class I and II.** Where multiple copies of genes are present in the genome, only the copy present in these gene clusters is shown. In the case of *Methanosarcinales* species only the HdrA1B1C1 genes are shown.

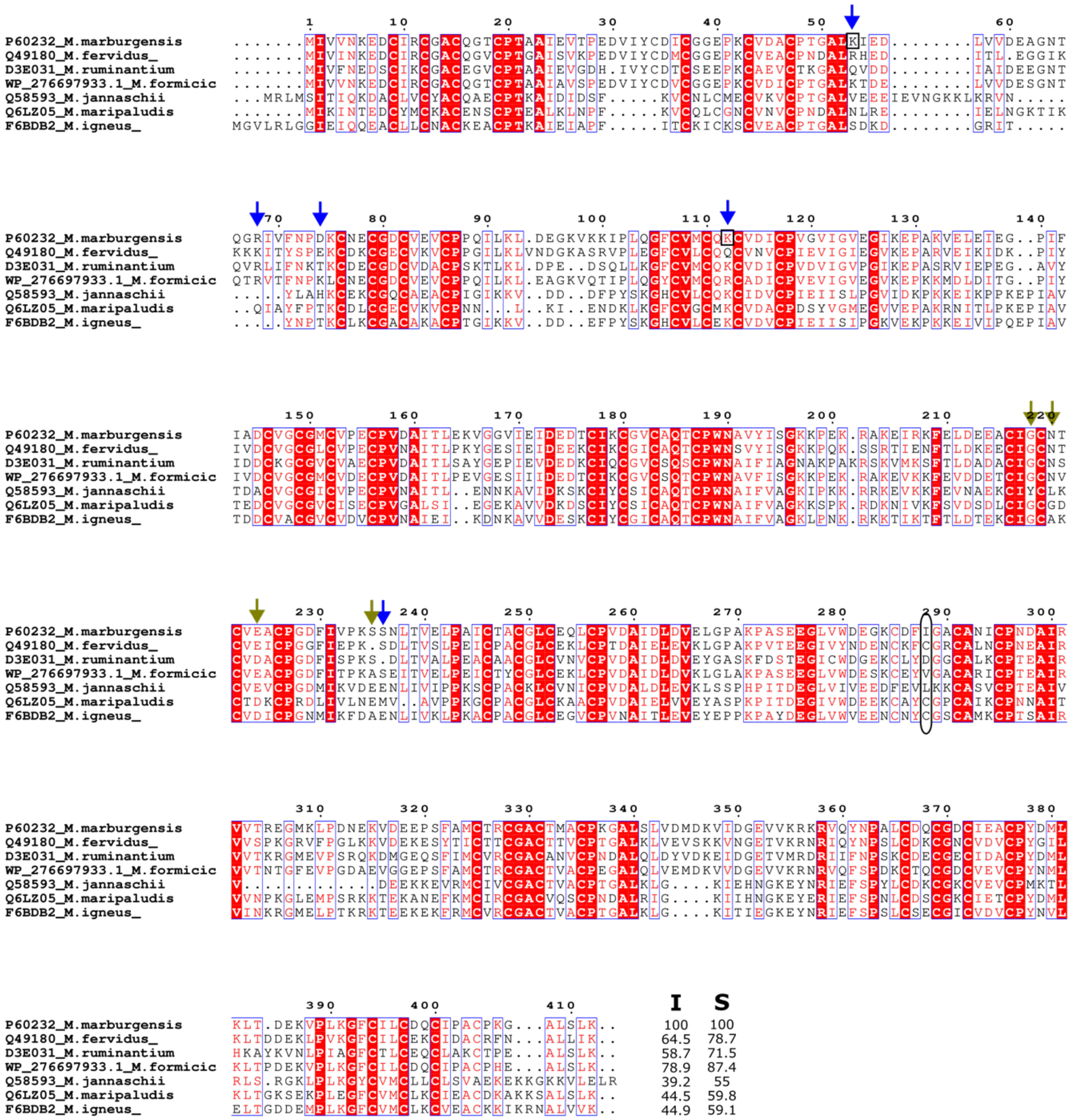


**Fig. S28. Sequence alignment of *M. marburgensis* MvhB with MvhB proteins of other representative species of class I.** Amino acid residues mediating the interaction with HdrA are marked with a blue arrow; amino acids mediating the interaction with FmdF are marked with a green arrow. *Methanococcus maripaludis (M. maripaludis)*, *Methanococcus igneus (M. igneus)*, *Methanobacterium formicicum (M. formicicum)*, *Methanothermus fervidus (M. fervidus), Methanocaldococcus jannaschii (M. jannaschii), Methanobrevibacter ruminantium* (*M. ruminantium*). The numbers indicate the position of amino acid residues in MvhB from *M. marburgensis*. A black oval indicates the position of a cysteine substitution explaining the presence of a [3Fe-4S] cluster in *M. marburgensis* MvhB. Percent sequence identity (I) and similarity (S) between *M. marburgensis* MvhB and the other MvhB proteins are indicated.

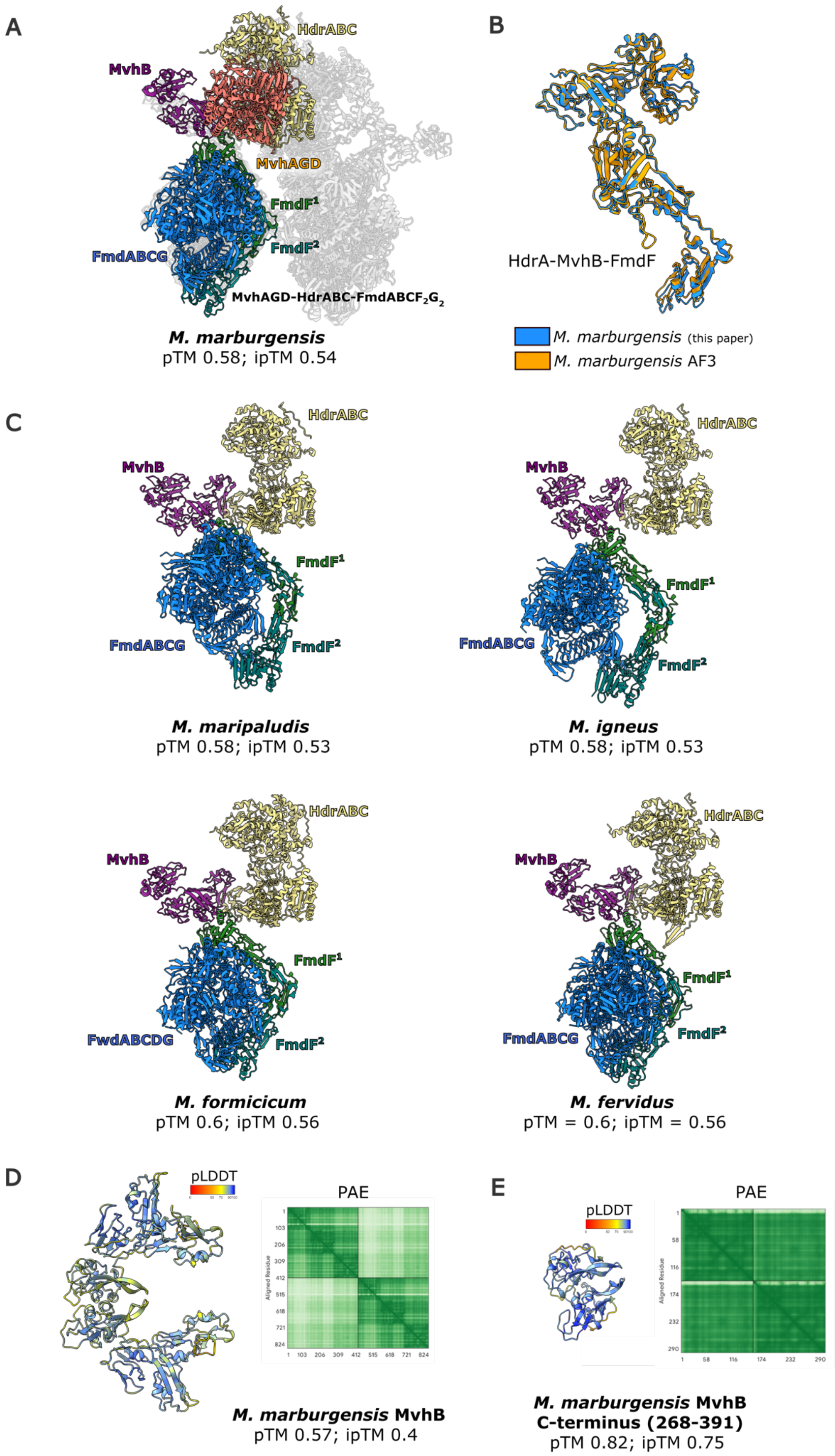
A
HdrABC
MvhB
MvhAGD
FmdF¹
FmdF²
FmdABCG
MvhAGD-HdrABC-FmdABCF₂G₂
M. marburgensis
pTM 0.58; ipTM 0.54
B
HdrA-MvhB-FmdF
M. marburgensis (this paper)
M. marburgensis AF3
C
HdrABC
MvhB
FmdF¹
FmdABCG
FmdF²
M. maripaludis
pTM 0.58; ipTM 0.53
HdrABC
MvhB
FmdF¹
FmdABCG
FmdF²
M. igneus
pTM 0.58; ipTM 0.53
HdrABC
MvhB
FmdF¹
FwdABCDG
FmdF²
M. formicicum
pTM 0.6; ipTM 0.56
HdrABC
MvhB
FmdF¹
FmdABCG
FmdF²
M. fervidus
pTM = 0.6; ipTM = 0.56
D
pLDDT
PAE
Aligned Residue
M. marburgensis MvhB
pTM 0.57; ipTM 0.4
E
pLDDT
PAE
Aligned Residue
M. marburgensis MvhB
C-terminus (268-391)
pTM 0.82; ipTM 0.75

**Fig. S29. MvhB is predicted to mediate the complexation of HdrA with FmdF, consistent with the C2-symmetric (Mvh-Hdr)$_2$-Fmd$_4$ complex, across different class I methanogenic species.** (**A**) AF3 predicts an interaction between HdrA, MvhB and FmdF of *M. marburgensis* (**B**) The AF3 prediction is very accurate, with an RMSD value of just 1.8 Å between the cryo-EM structure (blue) and the predicted HdrA-MvhB-Fmd unit (orange) after removal of the flexible MvhB C-terminal domain and the N- and C-terminal domains of HdrA. These domains are not displayed, as they are not predicted in the correct orientation by AF3. (**C**) AF3 predicts the formation of Mvh-Hdr-Fmd complexes mediated by MvhB in other representative members of Class I methanogens, including *Methanococcus maripaludis*, *Methanococcus igneus*, *Methanobacterium formicicum* and *Methanothermus fervidus*. (**D,E**) AlphaFold3 predicts dimerisation of the exposed MvhB C-terminal domain for predictions with (**D**) the full protein and (**E**) the C-terminal domain alone as input. Predicted template modeling score (pTM) and interface predicted modeling score (ipTM) values are shown. The Uniprot and NCBI codes for the protein sequences used in the AF3 predictions can be found in Table S3.

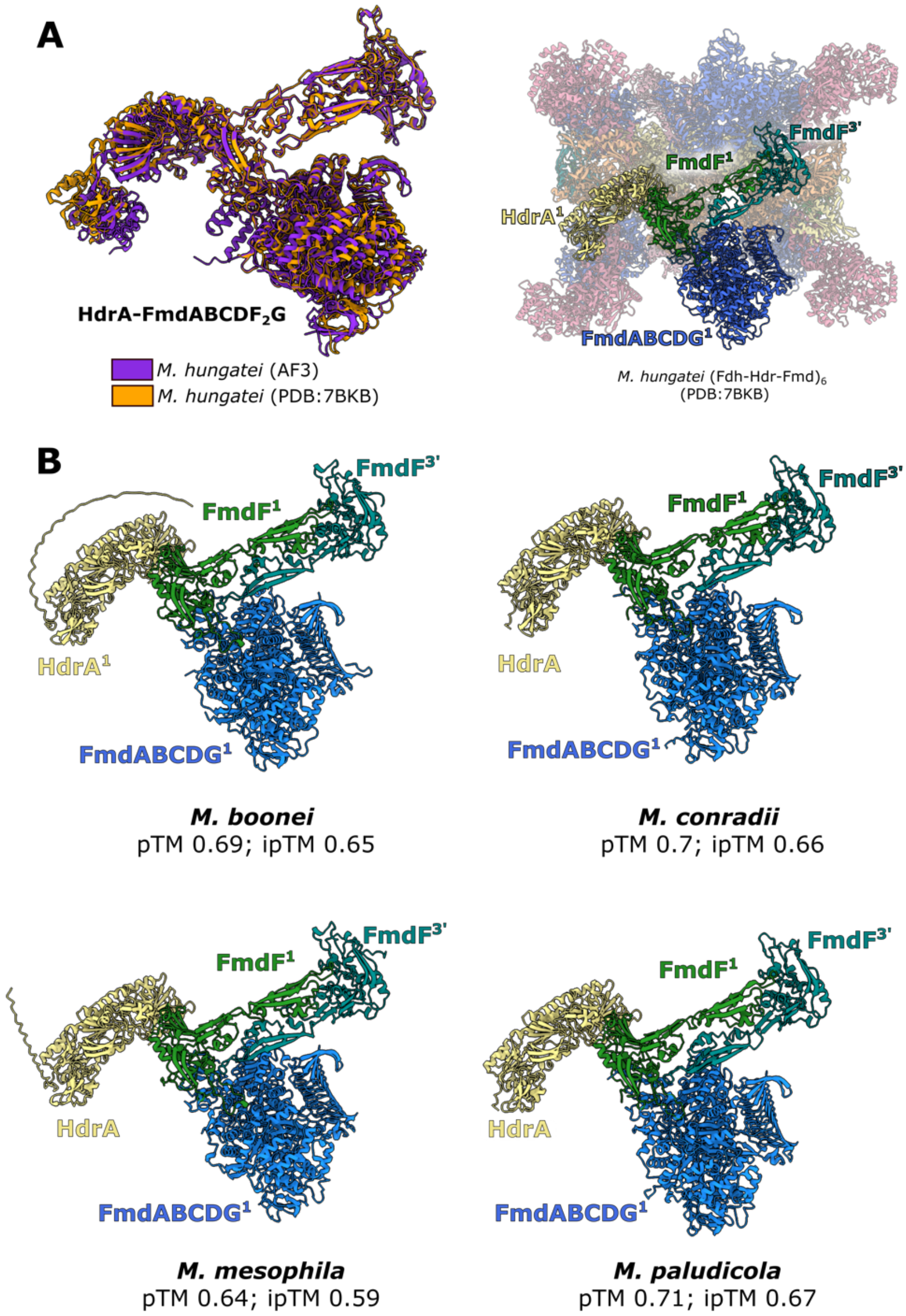


**Fig. S30. The complexation of HdrA, FmdF and FmdABCDG is predicted across class II methanogens.** (**A**) AF3 prediction of the structure of an *M. hungatei* HdrA-FmdABCDF$_2$G subcomplex, overlaid with the PDB model of the same complex (RMSD 4.82 Å with PDB:7BKB(*15*), left panel). The direct interaction between HdrA and dimeric FmdF enables a D3-symmetric hexameric conformation for the Fdh-Hdr-Fmd complex (right panel, PDB:7BKB(*15*)). (**B**) AF3 predicts the direct HdrA-FmdF interaction and FmdF dimerization required for the formation of hexameric Fdh(Mvh)-Hdr-Fmd complexes in other representative members of class IIa methanogens: *Methanoregula boonei*, *Methanocella conradii*, *Methanolinea mesophila* and *Methanocella paludicola*. Predicted template modeling score (pTM) and interface predicted modeling score (ipTM) values are shown. The Uniprot and NCBI codes for the sequences used for the AF3 predictions are listed in Table S3.

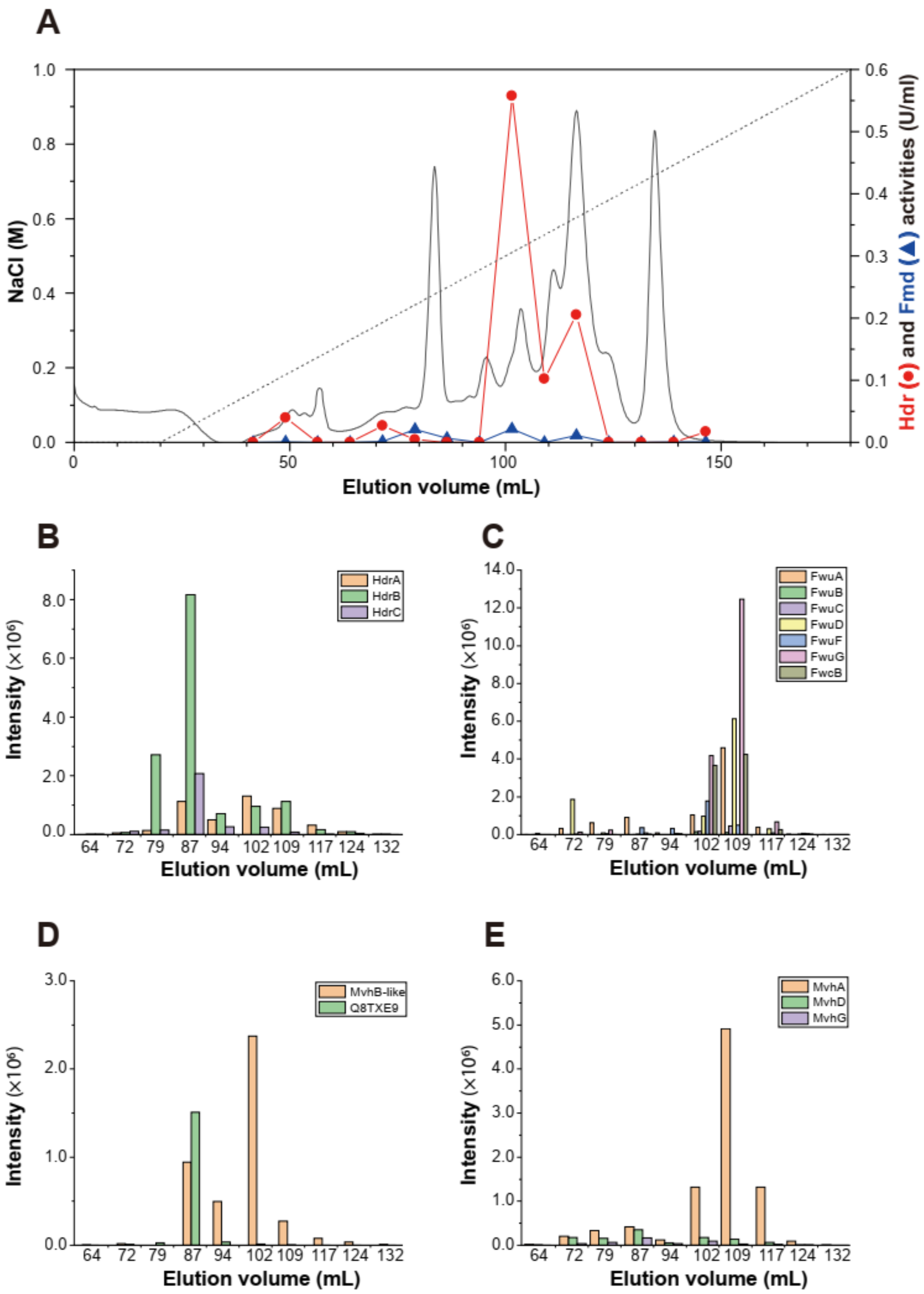


**Fig. S31. Fractionation of the Hdr complex from *M. kandleri* using Q-Sepharose (first experiment).** (**A**) Elution profile of the protein from the Q-Sepharose column with the Hdr and Fmd enzyme activity. UV absorbance (solid line) and NaCl-concentration gradient (dashed line) are shown. (**B–E**) Proteomic intensity in the fractions calculated as Intensity-Based Absolute Quantification (iBAQ); values for HdrABC (**B**), FwuABCDFG and FwuB (**C**), MvhB-like and Fe-S protein (Q8TXE9) (**D**), and MvhADG (**E**). Accession numbers of the proteins in the genome of *M. kandleri* AV19: HdrA, P96801; HdrB, Q8TXT8; HdrC, Q8TXT7; FwuF, Q8TYJ0; FwuA, Q8TV69; FwuC, Q8TV68; FwuD, Q1MVQ4; FwuB, Q8TV71; FwcB, Q8TYN5; FwuG, Q49611; MvhB-like, Q8TWN1; MvhA, Q8TYN0; MvhG, Q8TYM9; and MvhD, Q8TYM8. Q8TXE9 is a possible polyferredoxin.

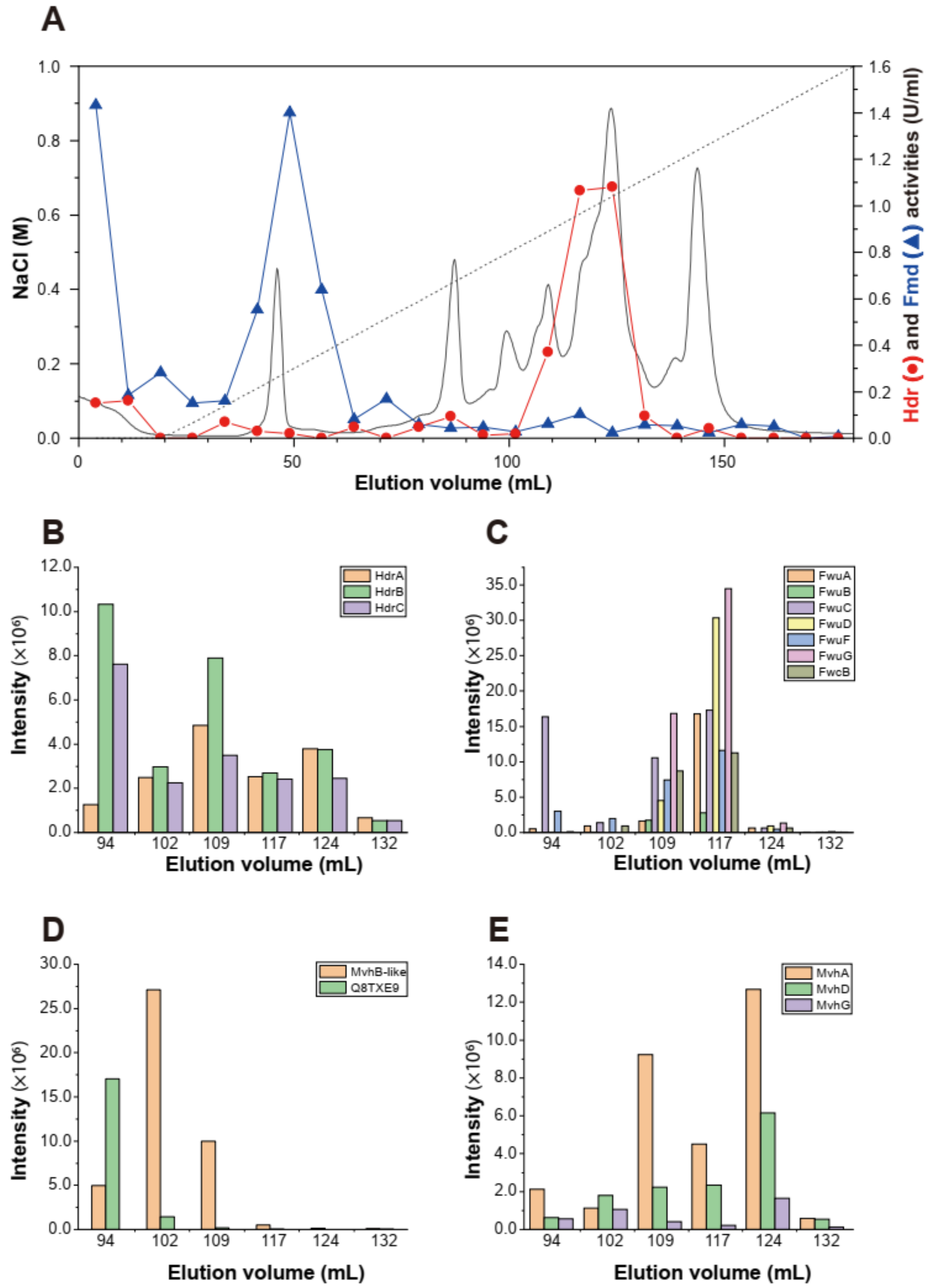


**Fig. S32. Fractionation of the Hdr complex from *M. kandleri* using Q-Sepharose (second experiment).** (**A**) Elution profile of the protein from the Q-Sepharose column with the Hdr and Fmd activity of the fractions. UV absorbance (solid line) and NaCl-concentration gradient (dashed line) are shown. (**B–E**) Proteomic intensity in the fractions calculated as iBAQ, for HdrABC (**B**), FwuABCDFG and FwuB (**C**), MvhB-like and Fe-S protein (Q8TXE9) (**D**), and MvhADG (**E**). For accession numbers of the proteins, see the legend of fig. S31.

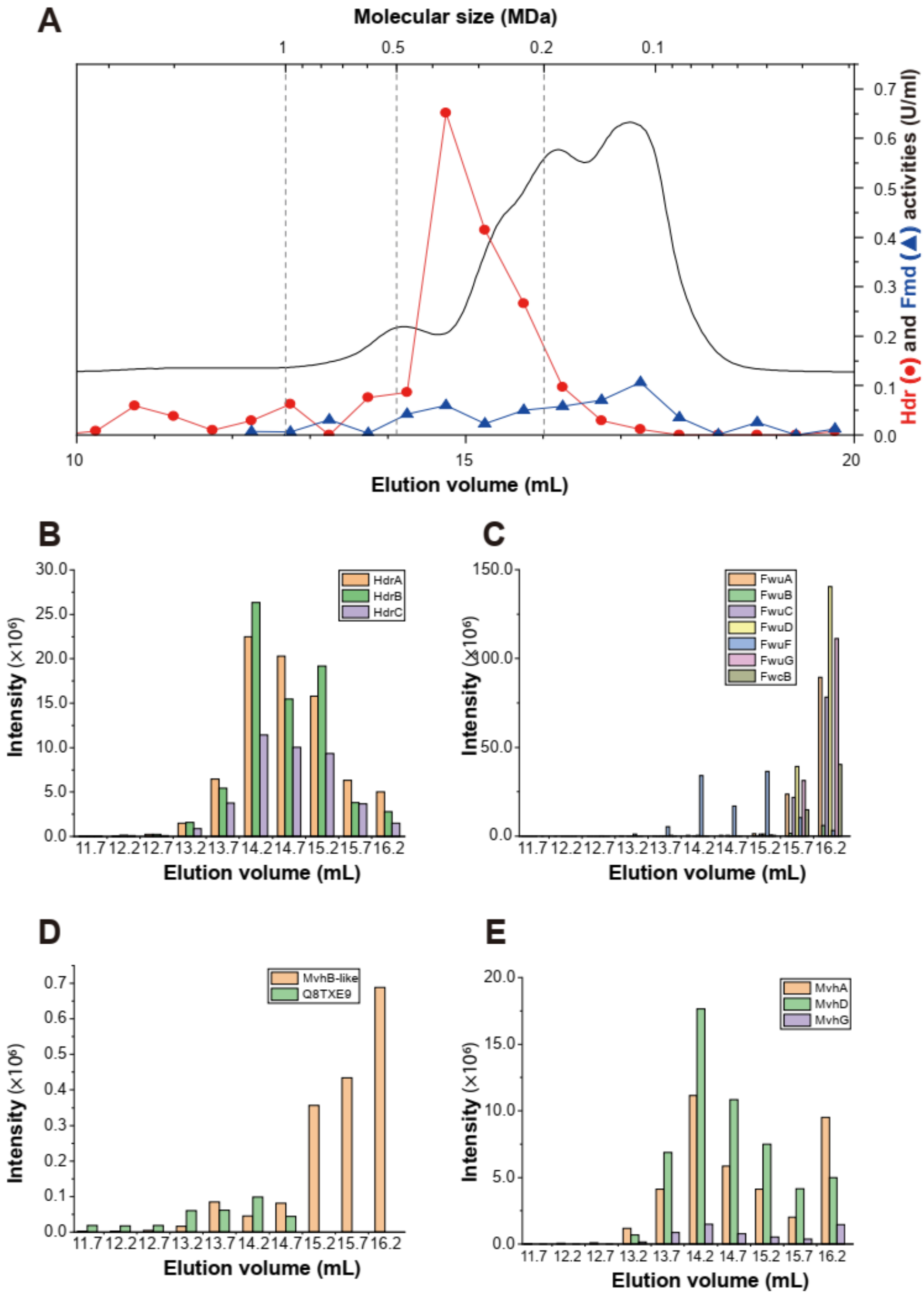


**Fig. S33. Fractionation of the Hdr complex from *M. kandleri* using SEC after Q-Sepharose (second experiment).** (**A**) Elution profile of the protein from the SEC with the Hdr and Fmd activity of the fractions. UV absorbance (solid line) and protein molecular weight (dashed line) are shown. (**B–E**) Proteomic intensity in the fractions calculated as iBAQ for HdrABC (**B**), FwuABCDFG and FwuB (**C**), MvhB-like and Fe-S protein (Q8TXE9) (**D**), and MvhADG (**E**). For accession numbers of the proteins, see the legend of fig. S31.

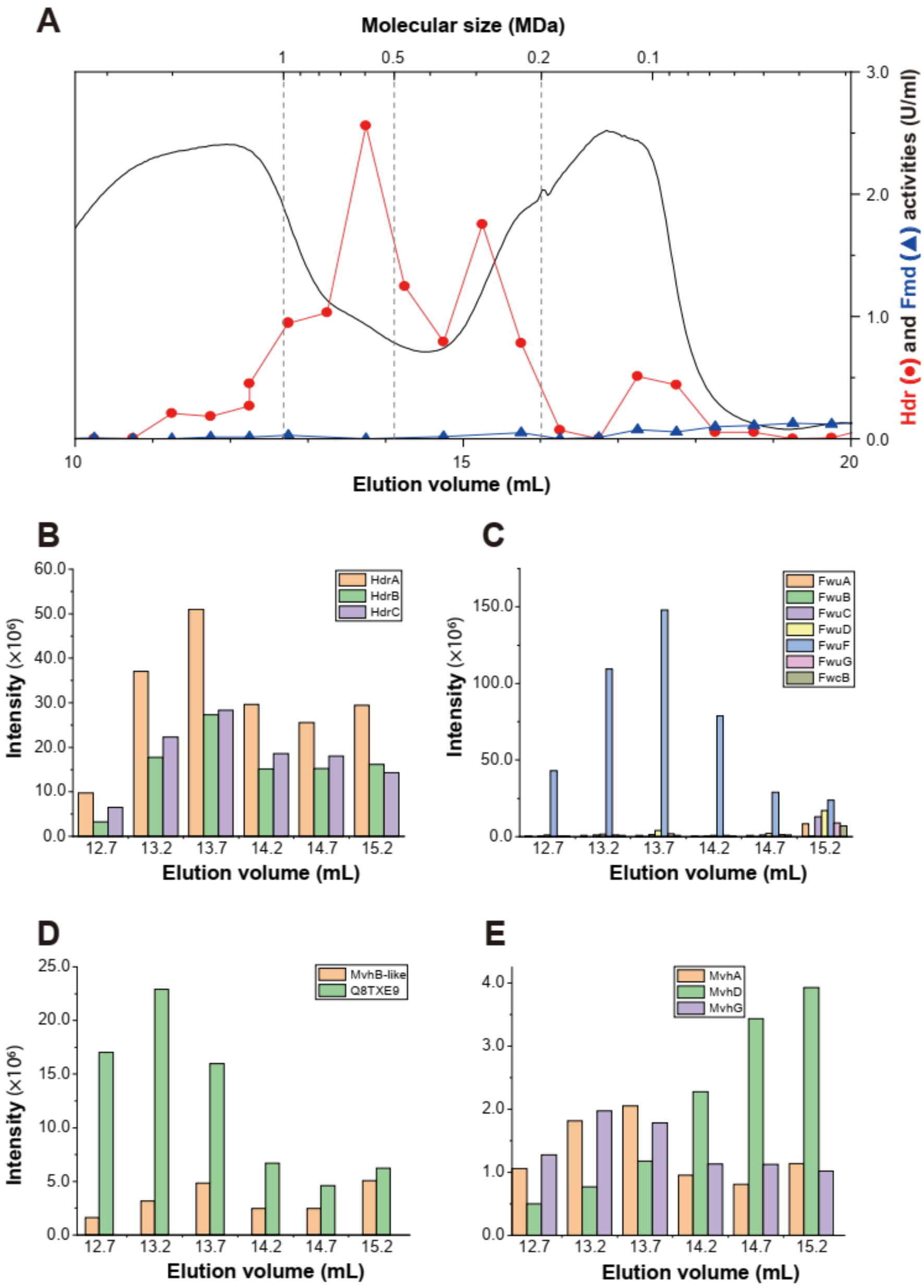


**Fig. S34. Fractionation of the Hdr complex from *M. kandleri* using SEC after ammonium sulfate precipitation (third experiment).** (**A**) Elution profile of the protein from the SEC with the Hdr and Fmd activity of the fractions. UV absorbance (solid line) and protein molecular weight (dashed line) are shown. (**B–E**) Proteomic intensity in the fractions calculated as iBAQ for HdrABC (**B**), FwuABCDFG and FwuB (**C**), MvhB-like and Fe-S protein (Q8TXE9) (**D**), and MvhADG (**E**). For accession numbers of the proteins, see the legend of fig. S31.

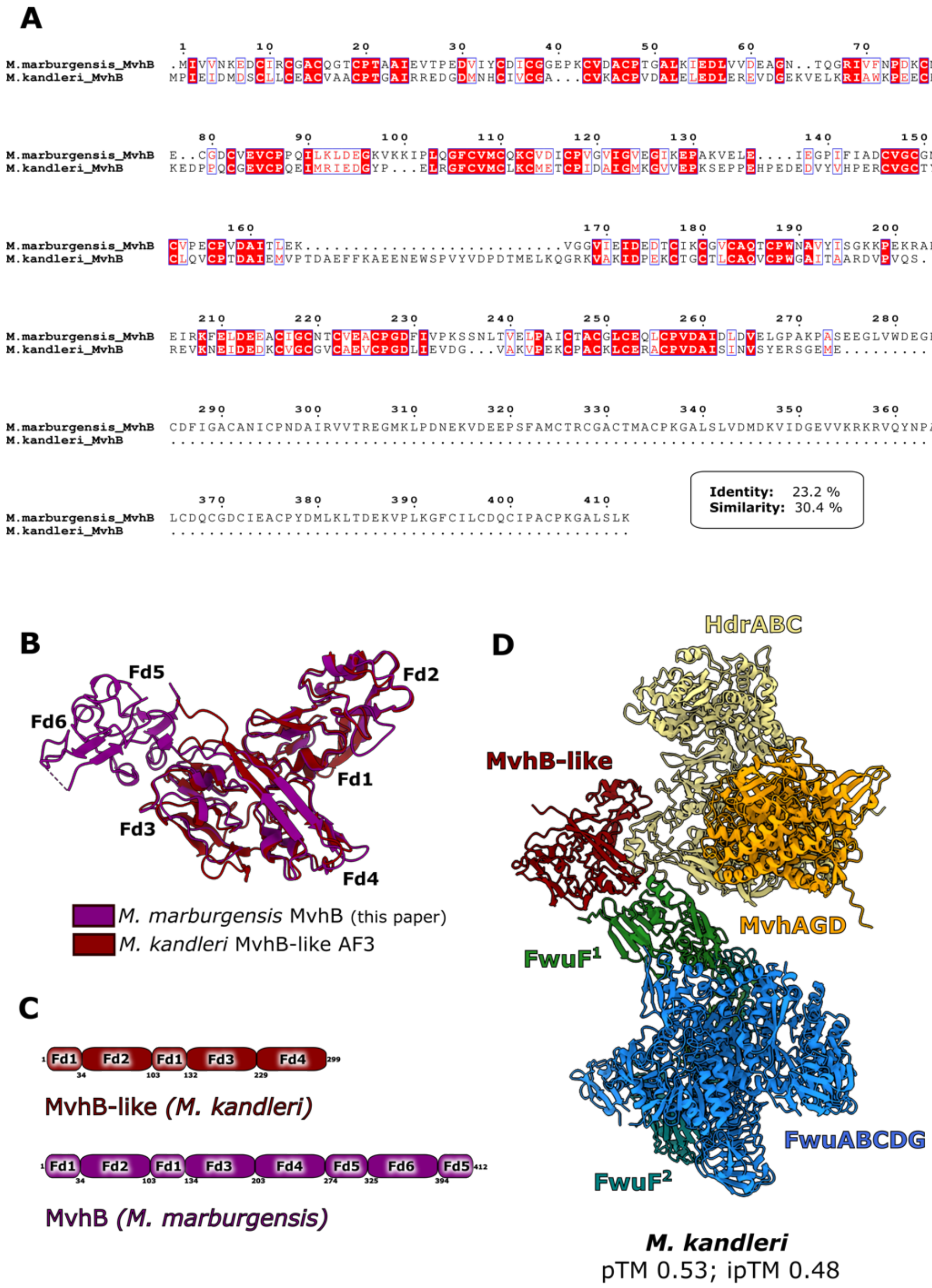


**Fig. S35.** ***M. kandleri*** **encodes an MvhB-like protein that lacks the C-terminal ferredoxin domains Fd5 and Fd6.** (**A**) Although the sequence alignment of *M. marburgensis* MvhB (P60232) and *M. kandleri* MvhB-like protein (Q8TWN1) reveals limited sequence similarity and identity (A), the AF3-predicted structure of M. kandleri MvhB-like protein reveals a highly similar 3D structure and domain organization to M. marburgensis MvhB (RMSD value of 5.351 Å). The *M. kandleri* MvhB-like protein lacks C-terminal ferredoxin domains Fd5 and Fd6 (**B** and **C**). Despite these differences, AF3 predicts the formation of an Mvh-Hdr-Fwu complex in *M. kandleri* mediated by the interaction of the MvhB-like protein with HdrA and FwuF (**D**). Predicted template modeling score (pTM) and interface predicted modeling score (ipTM) values are shown. Percent sequence identity (I) and similarity (S) are indicated.

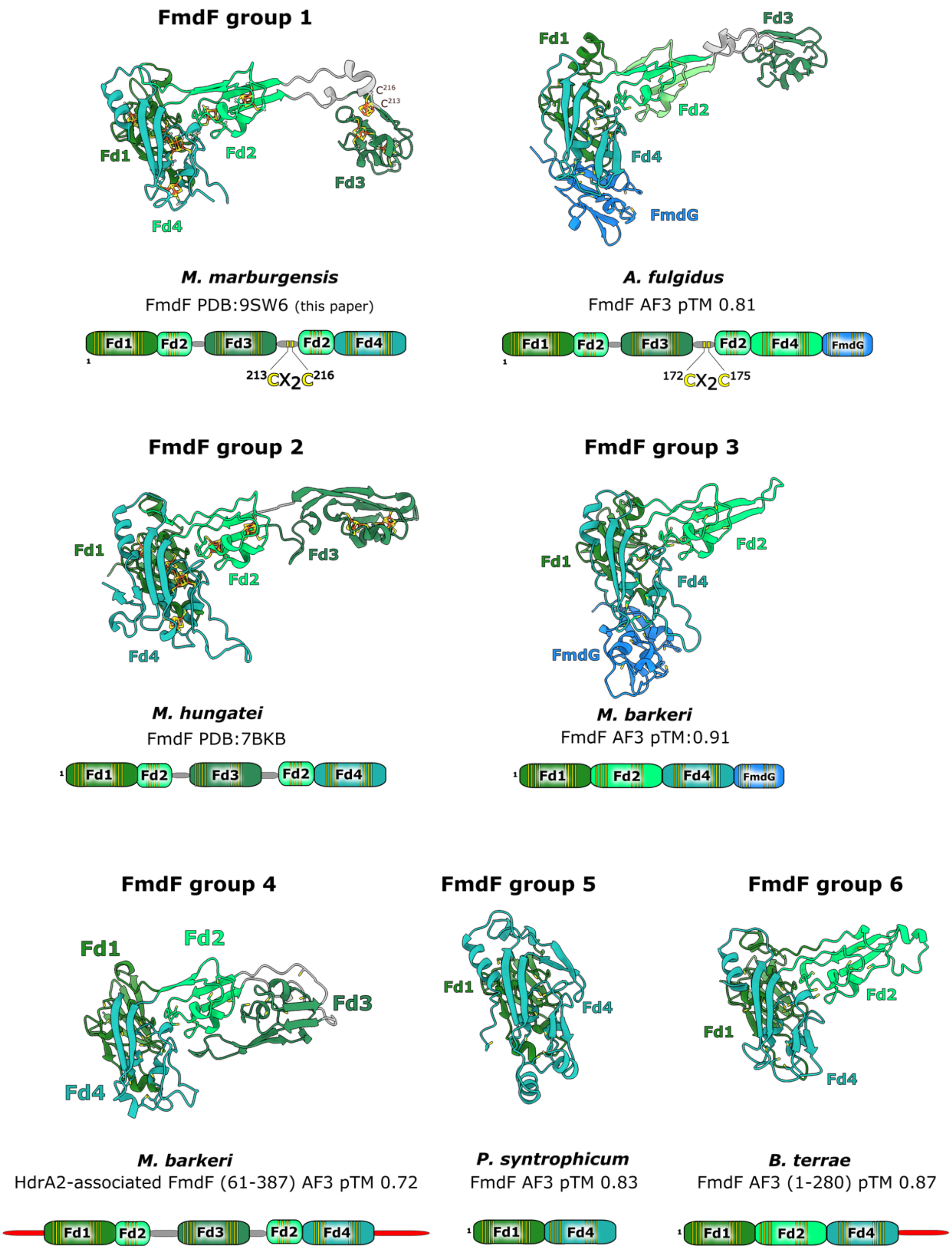


**Fig. S36. Cryo-EM structure of FmdF in this work (PDB IDs 9SW6 and 7BKB(*15*)) and AF3-predicted domain architecture of the FmdF groups.** Parts of the protein sequence that were predicted to be disordered are not shown; residue numbers shown are indicated in parentheses. Predicted template modeling score (pTM) is shown.

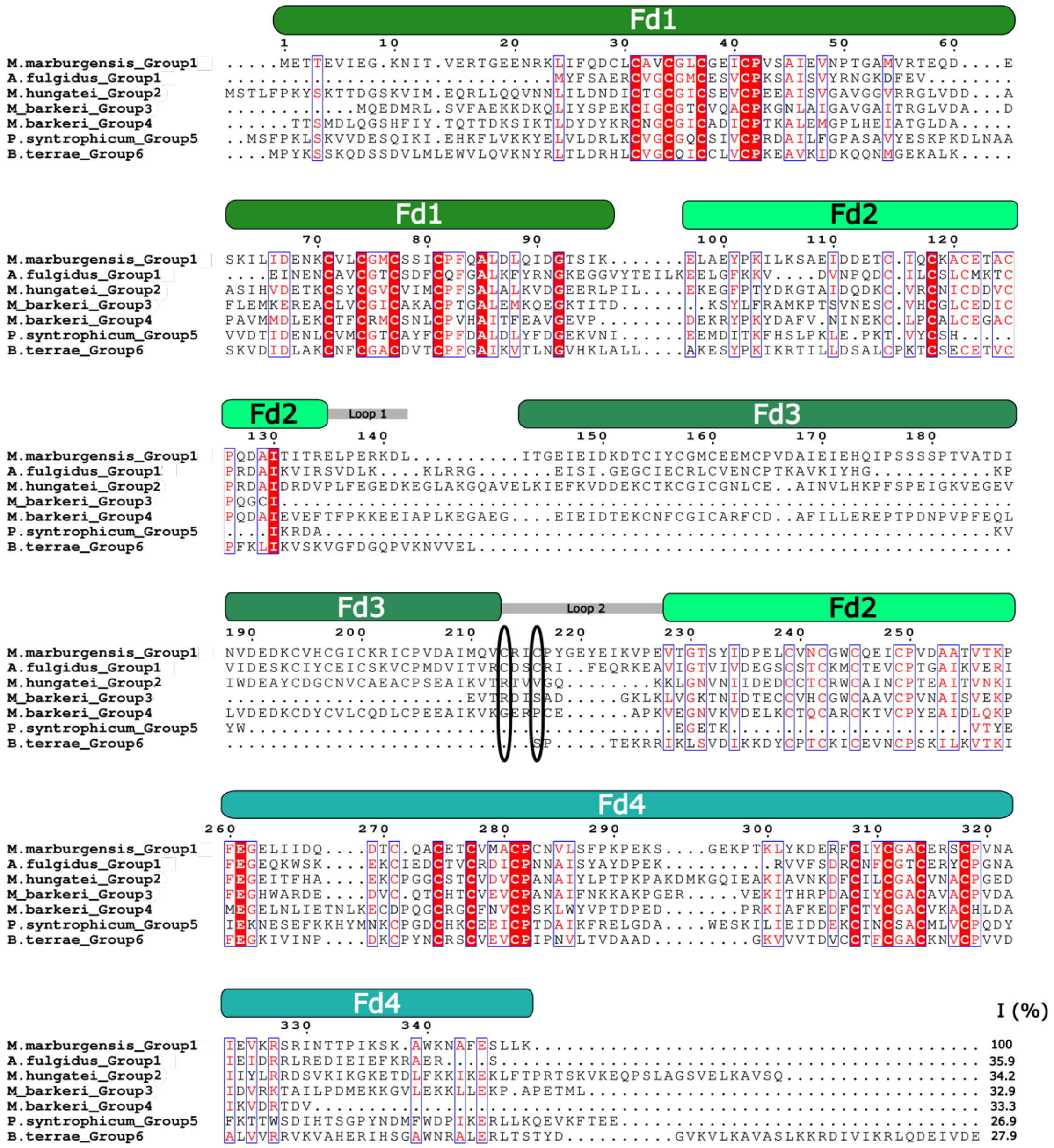

**Fig. S37. Sequence alignment of *M. marburgensis* FmdF with representative FmdF proteins of the different structural groups shown in fig. S38.** Cysteines in Fd3 loop 2 forming FF9 [4Fe-4S] cluster at the tetramerization interface of Group 1 FmdF and the non-conserved residues in Group 2–6 are squared. The domain organization of *M. marburgensis* is shown. (I) percent sequence identity. (Fd) ferredoxin domains. Accession codes for the sequences used in this alignment can be found in Table S4.

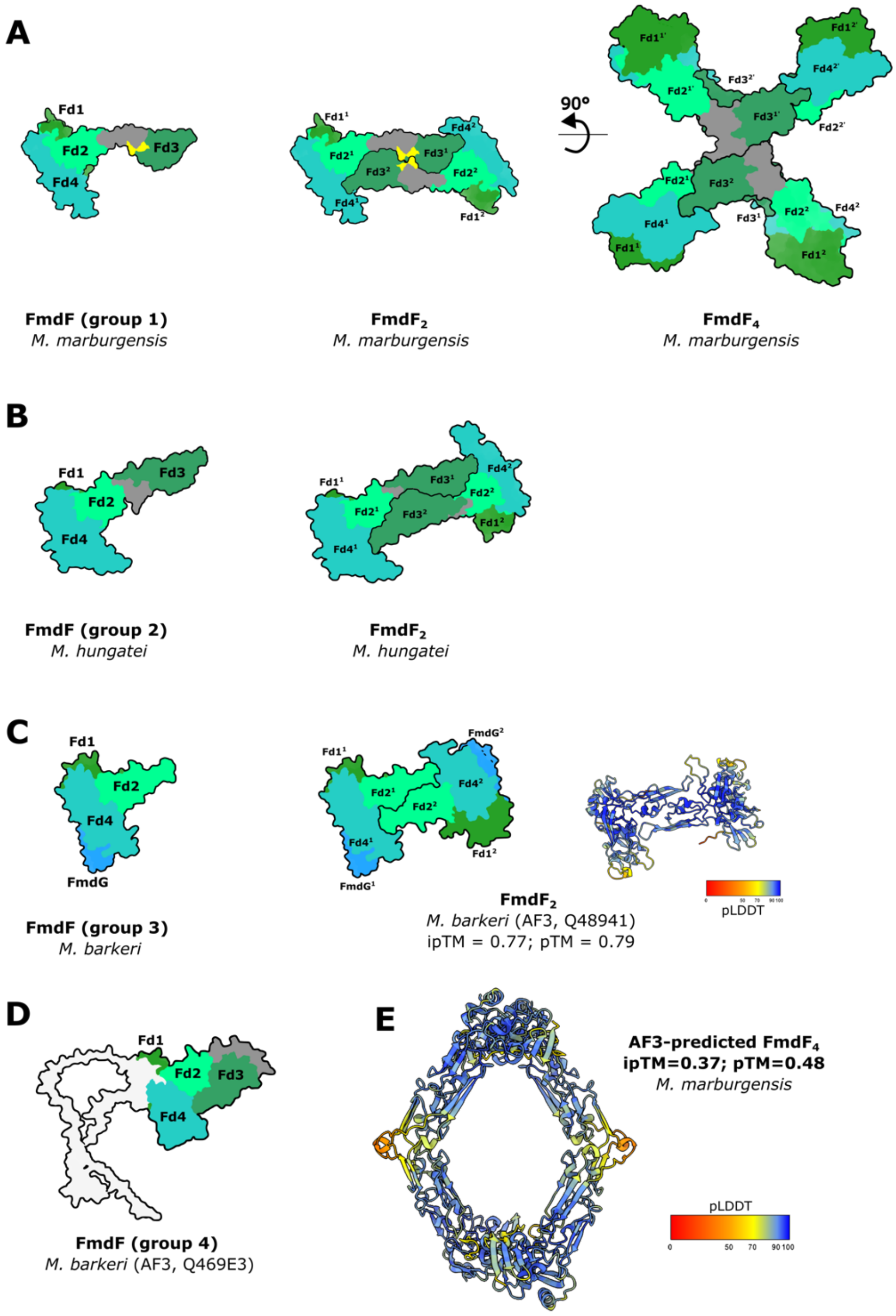


**Fig. S38. The mechanism of domain-swapping-based dimerization in FmdF.** Dimerisations of FmdFs belonging to Group 1 (**A**) and 2 (**B**) is mediated by the interaction of Fd3 domains of one monomer with the Fd2 domain of the other. For Group 3 FmdFs (**C**), dimerization predicted by AF3 is mediated by domain Fd2. In the AF3-predicted structures of FmdF members of Group 4 (**D**), the Fd3 domain of each monomer is interacts with the Fd2 domain of the same polypeptide chain, preventing dimerization. Cluster-ligating cysteines at the tetramerization interface in Group 1 FmdFs in **A** are shown in yellow. The predicted disordered N- and C-termini of Group 4 FmdFs are shown in light grey in **D**. The incorrect AF3-predicted structure of the FmdF tetramer of *M. marburgensis* is shown in E. pTM, predicted template modeling score; ipTM, interface predicted modeling score.

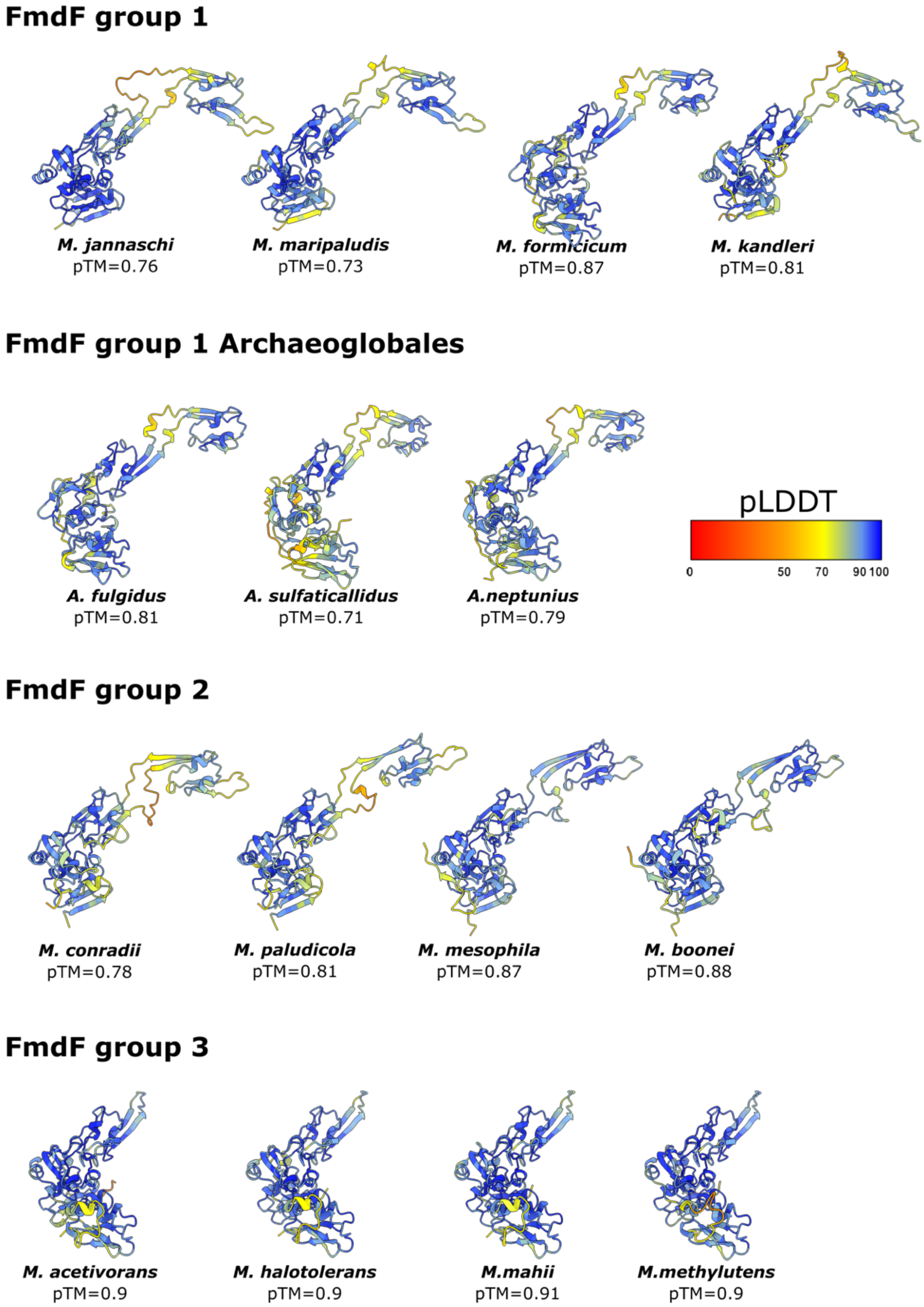


**Fig. S39.** AF3-predicted structures of FmdF groups 1 to 3 from representative species in class I and class II methanogens, sulfate-reducing archaea (Archaeoglobales) and Methanosarcinales. The structures were colored by pLDDT value.

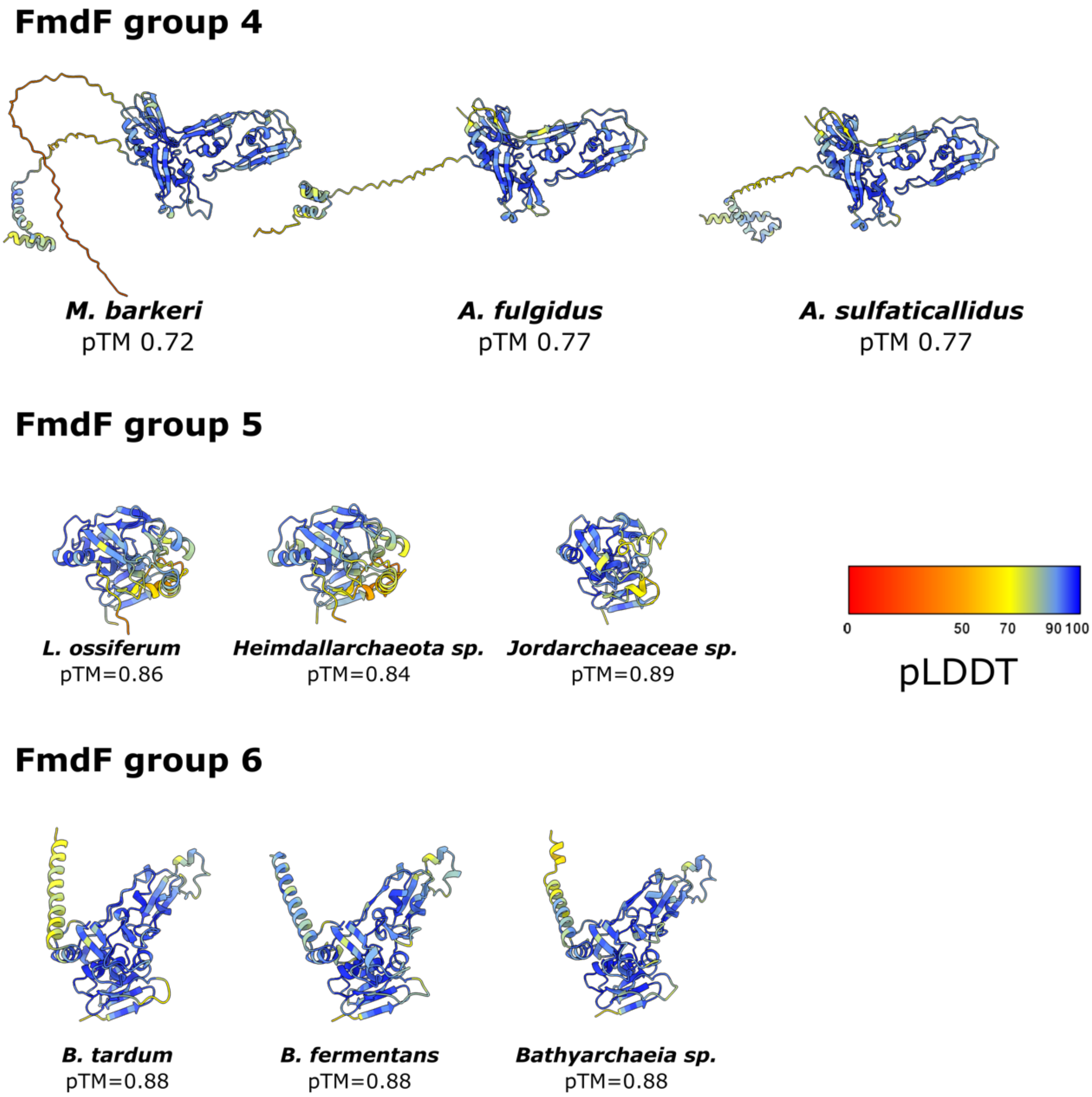


**Fig. S40. AF3-predicted structures of FmdF Groups 4 to 6 from representative species.** The structures were colored by pLDDT value.

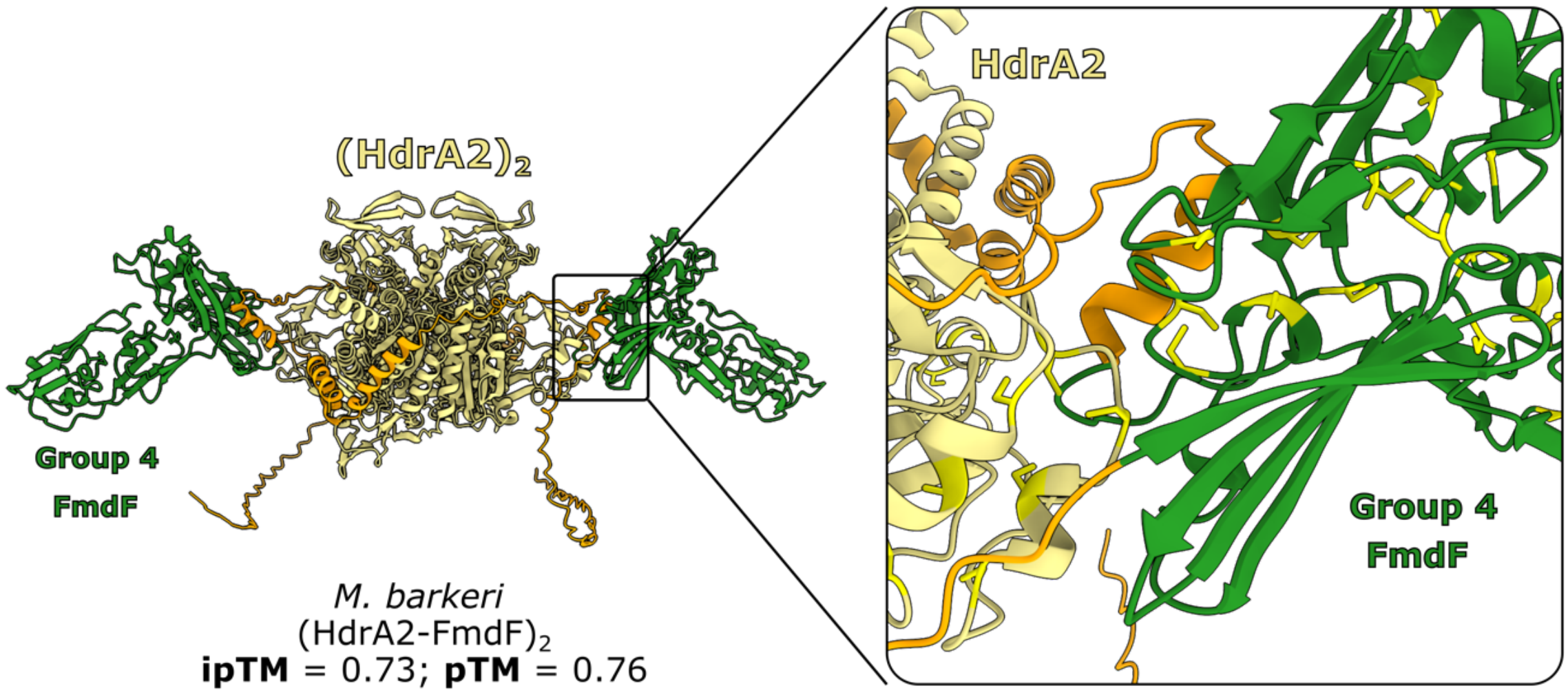


**Fig. S41. A complex between HdrA2 and Group 4 FmdF from *Methanosarcina barkeri* predicted by AF3.** In *Methanosarcinales*, Group 4 members are encoded in gene clusters containing an Hdr homolog (HdrA2) and none of the other Fmd-related genes. The N terminus of FmdF, shown in orange, is predicted to adopt a more structured conformation in the presence of $HdrA_2$. Predicted template modeling score (pTM) and interface predicted modeling score (ipTM) values are shown.

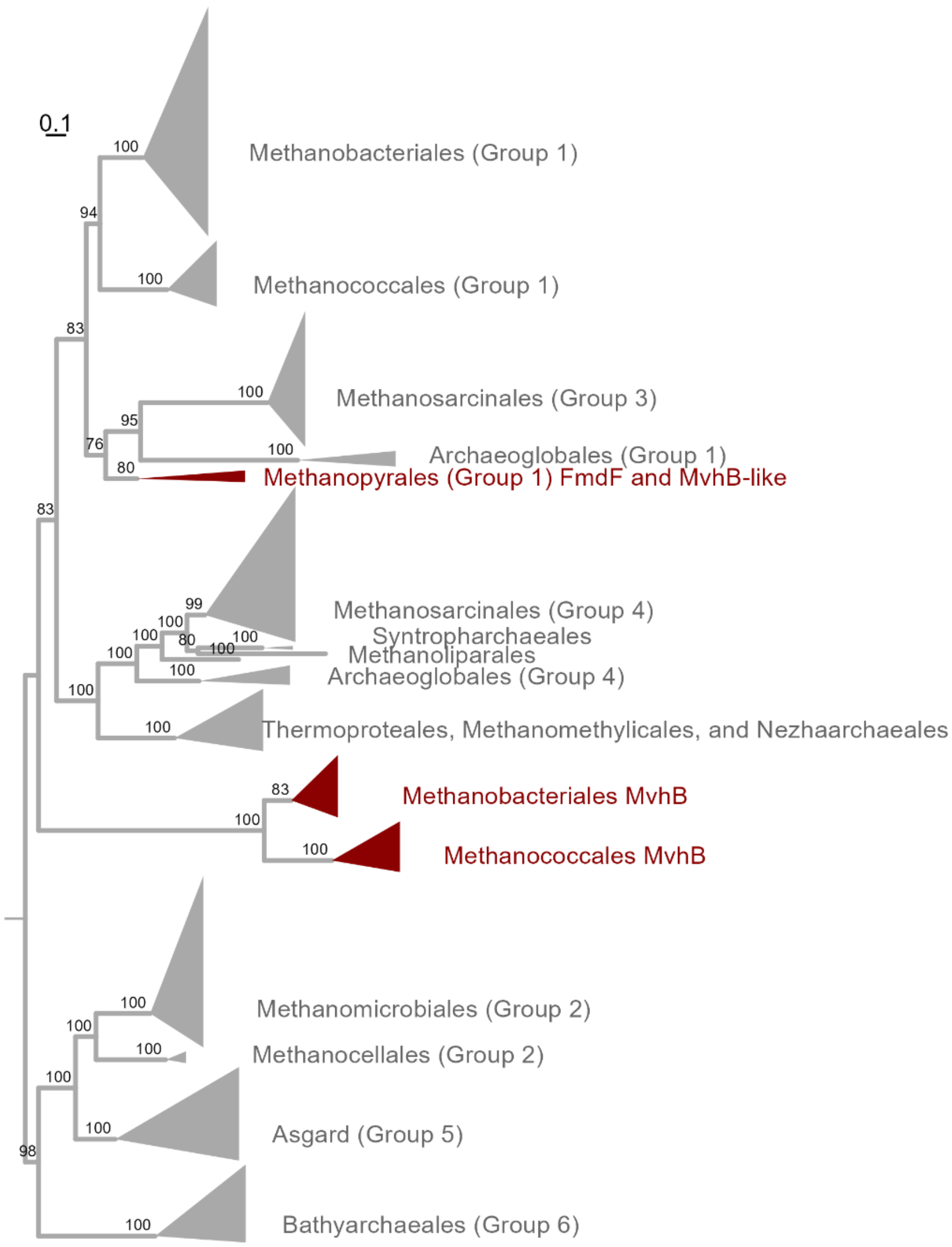


**Fig. S42. Maximum-likelihood phylogeny of FmdF homologs, including MvhB and MvhB-like sequences (red) added to avoid rooting bias.** Group numbers correspond to the groups described in Fig. S36.

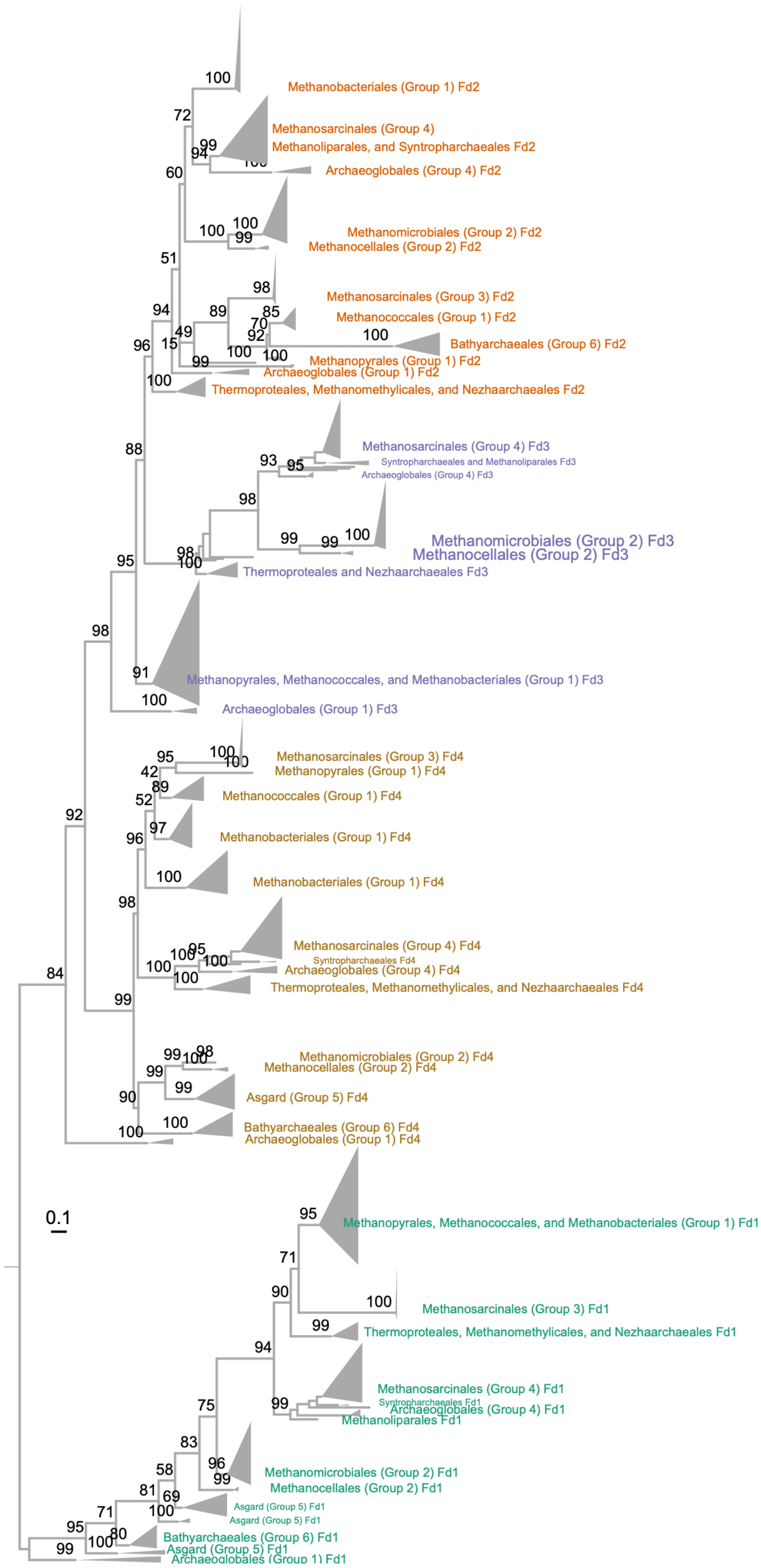


**Fig. S43. Phylogenetic tree based on the sequences of the FmdF domains. The domain sequences were separated in the sequence alignment used for the full FmdF phylogeny reconstruction using the structure of FmdF from *M. marburgensis* as reference. Fd1 (21-99), Fd2 (104-136 and 226-257), Fd3 (137-225), and Fd4 (258-349).**

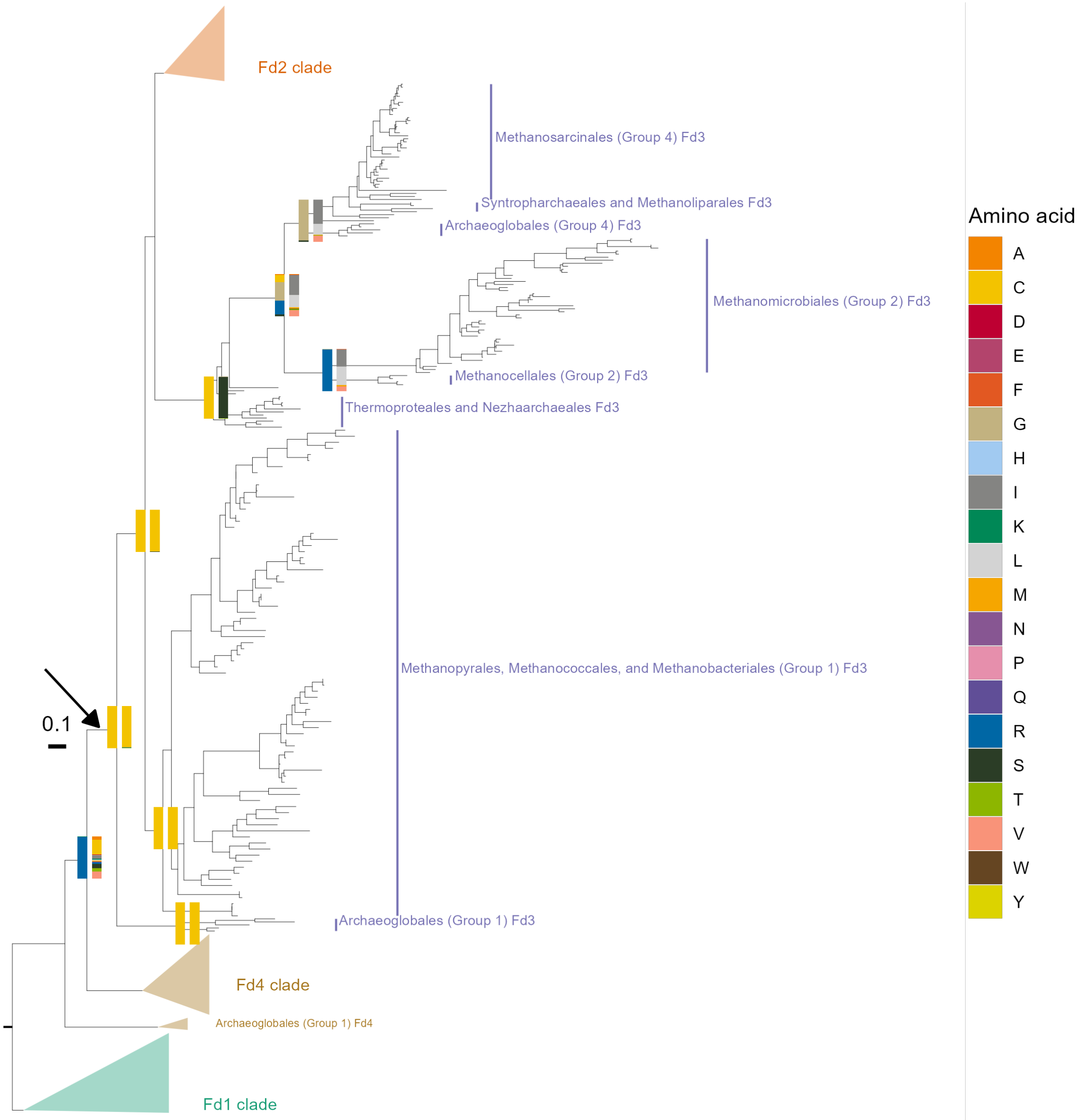


**Fig. S44. Ancestral sequence reconstruction of FmdF individual domains.** Colored bars at key nodes depict the probability of different amino acids occupying the two sites involved in tetramerization of FmdF. The presence of cysteine residues at both sites appears to be required for tetramerization. A black arrow indicates the specific internal node discussed in the main text.

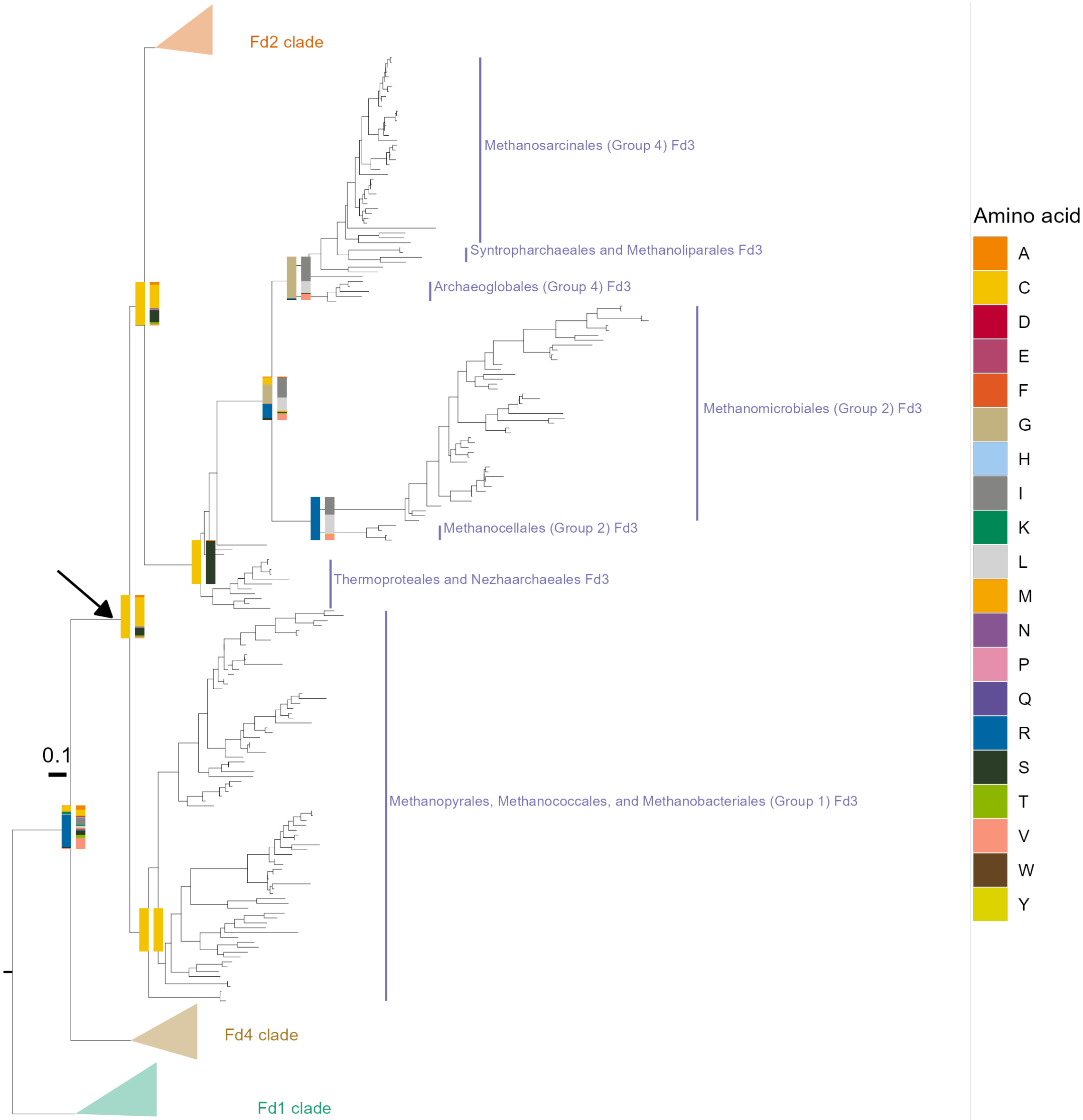


**Fig. S45. Ancestral sequence reconstruction of FmdF individual domains after pruning Archaeoglobales FmdF sequences fused to FmdG, which conflicted with the species tree.** Colored bars at key nodes depict the probability of different amino acids occupying the two sites involved in tetramerization of FmdF. The presence of cysteine residues at both sites appears to be required for tetramerization. A black arrow indicates the specific internal node discussed in the main text.

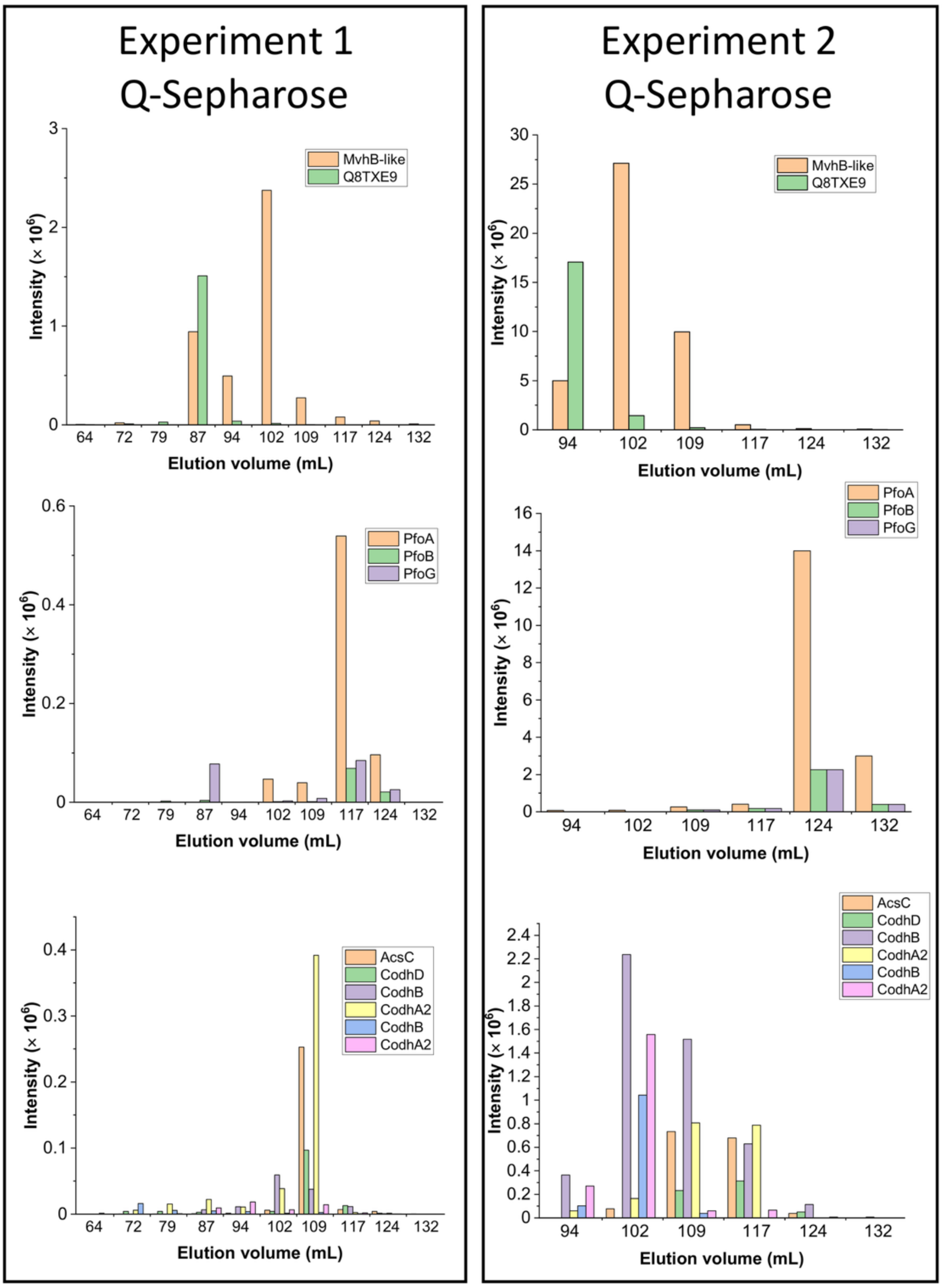


**Fig. S46. Proteomic analysis of *M. kandleri* PFOR, ACS, and CODH subunits in fractions eluted from Q-sepharose, experiments 1 and 2.**

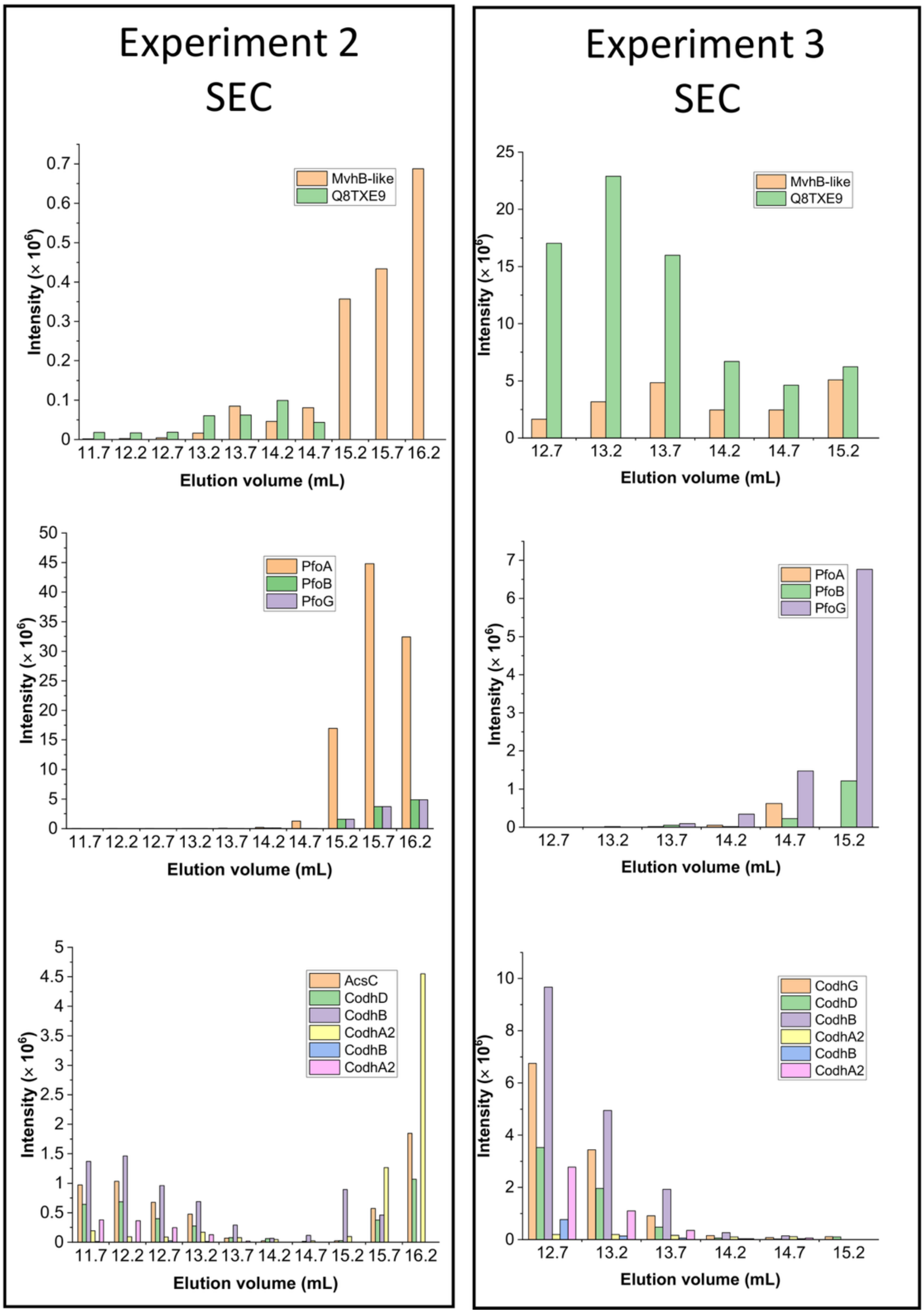


**Fig. S47. Proteomic analysis of *M. kandleri* PFOR, ACS, and CODH subunits in fractions from SEC, experiments 2 and 3.**

Table S1

| | Mobile arm state 1 substate a (EMD-55297) | | | | Mobile arm state 1 substate b (EMD-55298) | | | |
|---|---|---|---|---|---|---|---|---|
| | MvhAGD-focused map (EMD-55280) | HdrA-focused map (EMD-55281) | HdrBC-focused map (EMD-55279) | MvhAGD-HdrA consensus map (EMD-55289) | MvhAGD-focused map (EMD-55282) | HdrA-focused map (EMD-55283) | HdrBC-focused map (EMD-55285) | MvhAGD-HdrA consensus map (EMD-55284) |
| **Data collection and processing** | | | | | | | | |
| **Magnification** | 215,000 x | | | | | | | |
| **Voltage (kV)** | 300 | | | | | | | |
| **Electron exposure (e–/Å$^2$)** | 60 | | | | | | | |
| **Defocus range (µm)** | -0.8 to -2.2 | | | | | | | |
| **Pixel size (Å)** | 0.573 | | | | | | | |
| **Symmetry imposed** | C1 | | | | | | | |
| **Initial particle images (no.)** | 361,297 | | | | | | | |
| **Final particle images (no.)** | 12,602 (C2-expanded) | | | | 17,398 (C2-expanded) | | | |
| **B-factor** | -31.9 | -24.5 | -42 | -32.4 | -34 | -29.4 | -43 | -37 |
| **cFAR** | 0.04 | 0.09 | 0.08 | 0.05 | 0.08 | 0.14 | 0.11 | 0.07 |
| **SFC** | 0.614 | 0.606 | 0.613 | 0.613 | 0.568 | 0.558 | 0.574 | 0.558 |
| **Map resolution (Å) FSC threshold** | 0.143 | | | | | | | |
| **Map resolution range (Å)** | | | | | | | | |
| **Map resolution (Å) FSC 0.143 cut-off** | 2.9 | 2.8 | 3.42 | 2.88 | 2.83 | 2.82 | 3.18 | 3.14 |

Table S1

| | Mobile arm state 2 substate a (EMD-55295) | | | | Mobile arm state 2 substate b (EMD-55296) | | | |
|---|---|---|---|---|---|---|---|---|
| | MvhAGD-focused map (EMD-55287) | HdrA-focused map (EMD-55288) | HdrBC-focused map (EMD-55286) | MvhAGD-HdrA consensus map (EMD-55274) | MvhAGD-focused map (EMD-55275) | HdrA-focused map (EMD-55276) | HdrBC-focused map (EMD-55277) | MvhAGD-HdrA consensus map (EMD-55278) |
| **Data collection and processing** | | | | | | | | |
| **Magnification** | 215,000 x | | | | | | | |
| **Voltage (kV)** | 300 | | | | | | | |
| **Electron exposure (e–/Å²)** | 60 | | | | | | | |
| **Defocus range (µm)** | -0.8 to -2.2 | | | | | | | |
| **Pixel size (Å)** | 0.573 | | | | | | | |
| **Symmetry imposed** | C1 | | | | | | | |
| **Initial particle images (no.)** | 361,297 | | | | | | | |
| **Final particle images (no.)** | 167,927 (C2-expanded) | | | | 14,332 (C2-expanded) | | | |
| **B-factor** | -32.7 | -33.6 | -36.6 | -35.4 | -37.8 | -29.6 | -42.5 | -35.8 |
| **cFAR** | 0.23 | 0.27 | 0.31 | 0.24 | 0.13 | 0.11 | 0.11 | 0.12 |
| **SFC** | 0.623 | 0.624 | 0.624 | 0.613 | 0.546 | 0.542 | 0.548 | 0.541 |
| **Map resolution (Å) FSC threshold** | 0.143 | | | | | | | |
| **Map resolution range (Å)** | | | | | | | | |
| **Map resolution (Å) FSC 0.143 cut-off** | 2.05 | 2.14 | 2.14 | 2.19 | 3.07 | 2.94 | 3.07 | 3.04 |

Table S1

| | Mvh-Hdr-Fmd complex composite map (EMD-55299) | | | | | |
|---|---|---|---|---|---|---|
| | **FmdABCG[1]** EMD-55269 | **FmdABCG[2]** EMD-55271 | **FmdF$_2$** EMD-55272 | **MvhB** EMD-55270 | **MvhB C-terminus** EMD-55273 | **Full complex (consensus map)** EMD-55294 |
| **Data collection and processing** | | | | | | |
| **Magnification** | 215,000 x | | | | | |
| **Voltage (kV)** | 300 | | | | | |
| **Electron exposure (e–/Å$^2$)** | 60 | | | | | |
| **Defocus range (μm)** | -0.8 to -2.2 | | | | | |
| **Pixel size (Å)** | 0.573 | | | | | |
| **Symmetry imposed** | C1 | | | | | C2 |
| **Initial particle images (no.)** | 361,297 | | | | | |
| **Final particle images (no.)** | 263.229 (C2-expanded) | 105,282 (C2-expanded) | 630.434 (C2-expanded) | 322,503 (C2-expanded) | 322,503 (C2-expanded) | 361,297 (not expanded) |
| **B-factor** | -30.7 | -34.1 | -44.1 | -68.7 | -86.7 | -63.7 |
| **cFAR** | 0.23 | 0.32 | 0.17 | 0.28 | 0.08 | 0.17 |
| **SFC** | 0.744 | 0.771 | 0.915 | 0.747 | 0.774 | 0.92 |
| **Map resolution (Å) FSC threshold** | 0.143 | | | | | |
| **Map resolution range (Å)** | | | | | | |
| **Map resolution (Å) FSC 0.143 cut-off** | 1.93 | 2.19 | 2.12 | 2.47 | 2.82 | 2.45 |

**Table S1. Cryo-EM data collection and processing parameters.**

Table S2

| Refinement | State 1 substate a (PDB-9SW4, EMD-55297) | State 1 substate b (PDB-9SW5, EMD-55298) | State 2 substate a (PDB-9SW2, EMD-55295) | State 2 substate b (PDB-9SW3, EMD-55296) | Mvh-Hdr-Fmd (whole model, PDB-9SW6, EMD-55299) |
|---|---|---|---|---|---|
| Model resolution (Å) (masked/unmasked) | 3.16/3.82 | 3.16/3.78 | 2.32/2.68 | 3.21/3.95 | 2.33/2.82 |
| FSC threshold | 0.5 | 0.5 | 0.5 | 0.5 | 0.5 |
| Initial model used (PDB code) | State 2 substate a | State 2 substate a | None (Model-Angelo, sequence-based) | State 2 substate a | State 2 substate a |
| **Map sharpening *B* factor ($Å^2$)** | - | - | - | - | - |
| **Model composition** | | | | | |
| **Non-hydrogen atoms** | 15463 | 15404 | 15756 | - | 46976 |
| **Protein residues** | 1993 | 1977 | 2003 | - | 6012 |
| **Ligands** | | | | | |
| **SF4** | 11 | 11 | 11 | 11 | 45 |
| **9S8** | 2 | 2 | 2 | 2 | 2 |
| **FES** | 1 | 1 | 1 | 1 | 1 |
| **FAD** | 1 | 1 | 1 | 1 | 1 |
| **$H_2S$** | - | - | - | - | 2 |
| **Zn** | - | - | - | - | 4 |
| **MGD** | - | - | - | - | 4 |
| **NFU** | 1 | 1 | 1 | 1 | 1 |
| **F3S** | - | - | - | - | 1 |
| **Mo** | - | - | - | - | 2 |

Table S2

| | | | | | |
|---|---|---|---|---|---|
| **UNX** | 1 | 1 | 1 | 1 | 1 |
| **Waters** | - | - | 94 | - | 344 |
| ***B* factors (Å$^2$) mean** | | | | | |
| Protein | 53.9 | 47.31 | 62.31 | 83.08 | 39.06 |
| Ligand | 47.41 | 40.35 | 47.18 | 81.74 | 26.62 |
| Water | - | - | 49.04 | | 30.31 |
| **R.m.s.d deviations** | | | | | |
| Bond lengths (Å) | 0.003 | 0.005 | 0.004 | 0.004 | 0.008 |
| Bond angles (°) | 0.574 | 1.48 | 0.658 | 0.724 | 2.296 |
| **Validation** | | | | | |
| MolProbity score | 1.06 | 1.48 | 1.28 | 1.56 | 1.72 |
| Clash score | 7.02 | 5.75 | 3.42 | 6.15 | 8.99 |
| Poor rotamers (%) | 0.55 | 0.12 | 0.48 | 0.37 | 1.04 |
| **Ramach. plot** | | | | | |
| Favored (%) | 96.71 | 97.07 | 97.18 | 96.55 | 0.08 |
| Allowed (%) | 3.24 | 2.87 | 2.77 | 3.40 | 3.36 |
| Disallowed (%) | 0.05 | 0.05 | 0.05 | 0.05 | 96.56 |
| **Rama-Z** | | | | | |
| Whole | -0.48 | 0.12 | 0.82 | 0.68 | 0.24 |
| Helix | 0.08 | 0.72 | 1.53 | 1.59 | 0.97 |
| Sheet | 0.09 | 0.12 | 0.63 | 0.34 | 0.20 |
| Loop | -0.68 | -0.43 | -0.32 | -0.56 | -0.35 |
| **Q-score** | 0.70 | 0.71 | 0.81 | 0.67 | 0.82 |

**Table S2. Atomic model refinement and validation parameters.**

| Species/Subunits | MvhA | MvhG | MvhD | MvhB | HdrA | HdrB | HdrC | FmdA | FmdB /FwdB | FmdC /FwdC | FwdD | FmdF | FmdG |
|---|---|---|---|---|---|---|---|---|---|---|---|---|---|
| **Class I methanogens** | | | | | | | | | | | | | |
| *M. marburgensis* | P60227 | P60239 | P60238 | P60232 | Q50756 | Q50755 | Q50754 | ADL57752.1 | ADL58895.1 | ADL58894.1 | - | ADL57749.1 | ADL57750.1 |
| *M. maripaludis* | - | - | - | Q6LZ05 | WP_011170769 | Q6LYD8 | Q6LY39 | Q6LXU8 | Q6LZW1 | Q6LZW3 | - | Q6LXV1 | Q6LXV0 |
| *M. igneus* | - | - | - | F6BDB2 | WP_013799332 | F6BAD2 | F6BAD3 | F6BBL9 | F6BAR4 | F6BAR3 | - | F6BBL6 | F6BBL7 |
| *M. formicicum* | - | - | - | WP_276697933.1 | WP_004029400 | WP_004030536.1 | WP_048084665.1 | WP_281774550.1 | WP_004029669.1 | WP_004029668.1 | WP_004029666.1 | WP_004029664.1 | WP_004029665.1 |
| *M. fervidus* | - | - | - | Q49180 | ADP76987 | E3GZ06 | E3GZ07 | F6BBL9 | F6BE21 | F6BAR3 | - | E3GW07 | E3GW08 |
| *M. kandleri* | Q8TYN0 | Q8TYM9 | Q8TXN5 | Q8TWN1 | Q8TYP4 | Q8TXT8 | Q8TXT7 | Q8TV69 | Q8TYN5 | Q8TV68 | Q1MVQ4 | Q8TYJ0 | Q49611 |
| **Class II methanogens** | | | | | | | | | | | | | |
| *M. hungatei* | - | - | - | - | Q2FKZ1 | Q2FKZ2 | Q2FKZ3 | Q2FRL9 | Q2FRM0 | Q2FRL8 | Q2FRF6 | Q2FKZ4 | Q2FKZ5 |
| *M. boonei* | - | - | - | - | WP_232385642 | - | - | A7I8C9 | WP_012107027.1 | A7I8D0 | WP_012106420.1 | WP_012106130 | WP_012106131.1 |
| *M. mesophila* | - | - | - | - | WP_209673925 | - | - | WP_209674940.1 | WP_209674939.1 | WP_209673930.1 | WP_209673927.1 | WP_209673922 | WP_209673921.1 |
| *M. conradii* | - | - | - | - | WP_014407029 | - | - | H8I7I4 | WP_174591765.1 | H8I7I3 | WP_014407027.1 | H8I7J1 | WP_014407033.1 |
| *M. paludicola* | - | - | - | - | WP_012900327 | - | - | WP_012900323.1 | WP_012900324.1 | WP_012900322.1 | D1YYX4 | WP_012900330 | WP_012901514.1 |

**Table S3. Accession codes for protein and gene sequences used for AF3 predictions in this study,** shown in fig. S29, S30, S35.

| Species/Subunits | Accession code | AF3 prediction | |
|---|---|---|---|
| | | Dimer | Tetramer |
| **Group 1** | | | |
| *M. marburgensis* | *Q59575* | *Yes* | *No* |
| *M. maripaludis* | *Q6LXV1* | *Yes* | *Yes* |
| *M. jannaschi* | *WP_010870679* | *Yes* | *Yes* |
| *M. formicicum* | *WP_004029664.1* | *Yes* | *Yes* |
| *M. kandleri* | Q8TYJ0 | Yes | Yes |
| **Group 1 *Archaeoglobales*** | | | |
| *A. fulgidus* | WP_231487669.1 | Yes | No |
| *A. sulfaticallidus* | N0BA36 | Yes | No |
| *A. neptunius* | WP_202319219.1 | Yes | No |
| **Group 2** | | | |
| *M. boonei* | WP_012106130 (A7I5U8) | Yes | No |
| *M. hungatei* | Q2FKZ4 | Yes | No |
| *M. mesophila* | WP_209673922 | Yes | No |
| *M. conradii* | H8I7J1 | Yes | No |
| *M. paludicola* | WP_012900330 | Yes | No |
| **Group 3** | | | |
| *M. halotolerans* | A0A4E0PTW5 | Yes | No |
| *M. acetivorans* | Q8TIH5 | Yes | No |
| *M. mahii* | D5E6I3 | Yes | No |
| *M. methylutens* | A0A0E3SRG6 | Yes | No |
| *M. barkeri* | Q48941 | Yes | No |
| **Group 4** | | | |
| *A. fulgidus* | O28629 | No | No |
| *A. sulfaticallidus* | N0BDP2 | No | No |
| *M. barkeri* | Q469E3 FmdF | No | No |
| | Q469E2 HdrA2 | - | - |
| **Group 5** | | | |
| *L. osssiferum* | UYP43905 | No | No |
| *Heimdallarchaeota sp.* | MCK5342856 | No | No |
| *Jordarchaearceae sp.* | MEM3649397 | No | No |
| *P. syntrophicum* | WP_162306737 | No | No |
| **Group 6** | | | |
| *B. tardum* | MDG6222876 | No | No |
| *B. fermentans* | MCL1970375 | No | No |
| *Bathyarchaeia sp.* | MFA7398055 | No | No |
| *B. terrae* | MCL2691112 | No | No |
| | | | |

**Table S4. Accession codes for protein and gene sequences of FmdF, and predicted formation of dimeric and tetrameric states by AlphaFold3,** shown in fig. S36, S39-41.

**Table S5.** Enrichment of a possible Mvh-Hdr-Fmd complex from *M. kandleri*. Five grams of cells were disrupted by French press and fractionated according to the methods described in the methods section.

| | Fractions | Enzyme | Activity (U) | Protein (mg) | Specific activity (U/mg) | Purifi-cation (-fold) | Yield (%) |
|---|---|---|---|---|---|---|---|
| I[1] | CE | Hdr | 3.2 | 9.2 | 0.35 | 1 | 100 |
| | | Fmd | 4.3 | 9.2 | 0.47 | 1 | 100 |
| I | Q (14, 15, 16) | Hdr | 0.8 | 3.4 | 0.24 | 0.69 | 25 |
| | Q (11−16) | Fmd | 0.4 | 5.5 | 0.07 | 0.07 | 9 |
| | | | | | | | |
| II[2] | CE | Hdr | 11 | 46 | 0.24 | 1 | 100 |
| | | Fmd | 140 | 46 | 3.0 | 1 | 100 |
| II | Q (FT, 16, 17) | Hdr | 17 | 15 | 1.1 | 4.6 | >100 |
| | Q (FT, 1, 6, 7, 8) | Fmd | 180 | 41 | 4.4 | 1.5 | >100 |
| II | SEC (30, 31, 32)[4] | Hdr | 2.5 | 1,9 | 1.3 | 5.4 | 23 |
| | | | | | | | |
| III[3] | CE | Hdr | 66 | 64 | 1.0 | 1 | 100 |
| | | Fmd | 16 | 64 | 0.25 | 1 | 100 |
| III | AS at 60%[4,5] | Hdr | 13 | 12 | 1.1 | 1.1 | 20 |
| | | Fmd | 0.9 | 12 | 0.08 | 0.3 | 5 |
| III | SEC (12-16.5) | Hdr | 5.4 | 3.5 | 1.5 | 1.5 | 8 |
| | | Fmd | 0.11 | 3.5 | 0.03 | 0.12 | 0.7 |

[1]Fractionation using Q-Sepharose (Q) from the cell extract after ultracentrifugation, experiment 1; [2]Fractionation using Q-Sepharose and size-exclusion chromatography (SEC) from the cell extract after ultracentrifugation, experiment 2; [3]Fractionation by ammonium sulfate precipitation from supernatant of a low-speed centrifugation, experiment 3. [4]The precipitated fractions containing most Hdr activity were concentrated and applied to SEC; as the fractions showed very low Fmd activity after precipatation, the Fmd activity of SEC fractions was not determined. [5]The fraction precipitated by 60% saturated ammonium sulfate.

## REFERENCES


1. J. A. Rosentreter, A. V. Borges, B. R. Deemer, M. A. Holgerson, S. Liu, C. Song, J. Melack, P. A. Raymond, C. M. Duarte, G. H. Allen, D. Olefeldt, B. Poulter, T. I. Battin, B. D. Eyre, Half of global methane emissions come from highly variable aquatic ecosystem sources. *Nat. Geosci.* **14**, 225–230 (2021).

2. M. Saunois, A. R. Stavert, B. Poulter, P. Bousquet, J. G. Canadell, R. B. Jackson, P. A. Raymond, E. J. Dlugokencky, S. Houweling, P. K. Patra, P. Ciais, V. K. Arora, D. Bastviken, P. Bergamaschi, D. R. Blake, G. Brailsford, L. Bruhwiler, K. M. Carlson, M. Carrol, S. Castaldi, N. Chandra, C. Crevoisier, P. M. Crill, K. Covey, C. L. Curry, G. Etiope, C. Frankenberg, N. Gedney, M. I. Hegglin, L. Höglund-Isaksson, G. Hugelius, M. Ishizawa, A. Ito, G. Janssens-Maenhout, K. M. Jensen, F. Joos, T. Kleinen, P. B. Krummel, R. L. Langenfelds, G. G. Laruelle, L. Liu, T. Machida, S. Maksyutov, K. C. McDonald, J. McNorton, P. A. Miller, J. R. Melton, I. Morino, J. Müller, F. Murguia-Flores, V. Naik, Y. Niwa, S. Noce, S. O'Doherty, R. J. Parker, C. Peng, S. Peng, G. P. Peters, C. Prigent, R. Prinn, M. Ramonet, P. Regnier, W. J. Riley, J. A. Rosentreter, A. Segers, I. J. Simpson, H. Shi, S. J. Smith, L. P. Steele, B. F. Thornton, H. Tian, Y. Tohjima, F. N. Tubiello, A. Tsuruta, N. Viovy, A. Voulgarakis, T. S. Weber, M. van Weele, G. R. van der Werf, R. F. Weiss, D. Worthy, D. Wunch, Y. Yin, Y. Yoshida, W. Zhang, Z. Zhang, Y. Zhao, B. Zheng, Q. Zhu, Q. Zhu, Q. Zhuang, The global methane budget 2000–2017. *Earth Syst. Sci. Data* **12**, 1561–1623 (2020).

3. A. J. Kohtz, N. Petrosian, V. Krukenberg, Z. J. Jay, M. Pilhofer, R. Hatzenpichler, Cultivation and visualization of a methanogen of the phylum Thermoproteota. *Nature* **632**, 1118–1123 (2024).

4. K. W. Seitz, N. Dombrowski, L. Eme, A. Spang, J. Lombard, J. R. Sieber, A. P. Teske, T. J. G. Ettema, B. J. Baker, Asgard archaea capable of anaerobic hydrocarbon cycling. *Nat. Commun.* **10**, 1822 (2019).

5. Y. Wang, G. Wegener, T. A. Williams, R. Xie, J. Hou, C. Tian, Y. Zhang, F. Wang, X. Xiao, A methylotrophic origin of methanogenesis and early divergence of anaerobic multicarbon alkane metabolism. *Sci. Adv.* **7**, eabj1453 (2021).

6. P. S. Garcia, S. Gribaldo, G. Borrel, Diversity and evolution of methane-related pathways in Archaea. *Annu. Rev. Microbiol.* **76**, 727–755 (2022).

7. G. Borrel, P. S. Adam, S. Gribaldo, Methanogenesis and the Wood-Ljungdahl pathway: An ancient, versatile, and fragile association. *Genome Biol. Evol.* **8**, 1706–1711 (2016).

8. T. Wagner, U. Ermler, S. Shima, The methanogenic $CO_2$ reducing-and-fixing enzyme is bifunctional and contains 46 [4Fe-4S] clusters. *Science* **354**, 114–117 (2016).

9. T. Wagner, J. Koch, U. Ermler, S. Shima, Methanogenic heterodisulfide reductase (HdrABC-MvhAGD) uses two noncubane [4Fe-4S] clusters for reduction. *Science* **357**, 699–703 (2017).

10. A. K. Kaster, J. Moll, K. Parey, R. K. Thauer, Coupling of ferredoxin and heterodisulfide reduction via electron bifurcation in hydrogenotrophic methanogenic archaea. *Proc. Natl. Acad. Sci. U.S.A.* **108**, 2981–2986 (2011).

11. C. E. Lubner, D. P. Jennings, D. W. Mulder, G. J. Schut, O. A. Zadvornyy, J. P. Hoben, M. Tokmina-Lukaszewska, L. Berry, D. M. Nguyen, G. L. Lipscomb, B. Bothner, A. K. Jones, A.-F. Miller, P. W. King, M. W. W. Adams, J. W. Peters, Mechanistic insights into energy conservation by flavin-based electron bifurcation. *Nat. Chem. Biol.* **13**, 655–659 (2017).

12. W. Buckel, R. K. Thauer, Flavin-based electron bifurcation, a new mechanism of biological energy coupling. *Chem. Rev.* **118**, 3862–3886 (2018).

13. W. Buckel, R. K. Thauer, Flavin-based electron bifurcation, ferredoxin, flavodoxin, and anaerobic respiration with protons (Ech) or $NAD^+$ (Rnf) as electron acceptors: A historical review. *Front. Microbiol.* **9**, 401 (2018).

14. R. K. Thauer, Methyl (alkyl)-coenzyme M reductases: Nickel F-430-containing enzymes involved in anaerobic methane formation and in anaerobic oxidation of methane or of short chain alkanes. *Biochemistry* **58**, 5198–5220 (2019).

15. T. Watanabe, O. Pfeil-Gardiner, J. Kahnt, J. Koch, S. Shima, B. J. Murphy, Three-megadalton complex of methanogenic electron-bifurcating and $CO_2$-fixing enzymes. *Science* **373**, 1151–1156 (2021).

16. K. C. Costa, P. M. Wong, T. Wang, T. J. Lie, J. A. Dodsworth, I. Swanson, J. A. Burn, M. Hackett, J. A. Leigh, Protein complexing in a methanogen suggests electron bifurcation and electron delivery from formate to heterodisulfide reductase. *Proc. Natl. Acad. Sci. U.S.A.* **107**, 11050–11055 (2010).

17. S. Nomura, P. San Segundo-Acosta, E. Protasov, M. Kaneko, J. Kahnt, B. J. Murphy, S. Shima, Electron flow in hydrogenotrophic methanogens under nickel limitation. *Nature* **644**, 490–496 (2025).

18. T. Watanabe, S. Shima, MvhB-type polyferredoxin as an electron-transfer chain in putative redox-enzyme complexes. *Chem. Lett.* **50**, 353–360 (2021).

19. S. Nomura, N. Paczia, J. Kahnt, S. Shima, Isolation of an $H_2$-dependent electron-bifurcating $CO_2$-reducing megacomplex with MvhB polyferredoxin from *Methanothermobacter marburgensis*. *FEBS J.* **291**, 2449–2460 (2024).

20. R. Hedderich, S. P. Albracht, D. Linder, J. Koch, R. K. Thauer, Isolation and characterization of polyferredoxin from *Methanobacterium thermoautotrophicum*. The mvhB gene product of the methylviologen-reducing hydrogenase operon. *FEBS Lett.* **298**, 65–68 (1992).

21. M. Kurr, R. Huber, H. König, H. W. Jannasch, H. Fricke, A. Trincone, J. K. Kristjansson, K. O. Stetter, *Methanopyrus kandleri*, gen. and sp.-nov. represents a novel group of hyperthermophilic methanogens, growing at 110°C. *Arch. Microbiol.* **156**, 239–247 (1991).

22. A. I. Slesarev, K. V. Mezhevaya, K. S. Makarova, N. N. Polushin, O. V. Shcherbinina, V. V. Shakhova, G. I. Belova, L. Aravind, D. A. Natale, I. B. Rogozin, R. L. Tatusov, Y. I. Wolf, K. O. Stetter, A. G. Malykh, E. V. Koonin, S. A. Kozyavkin, The complete genome of hyperthermophile *Methanopyrus kandleri* AV19 and monophyly of archaeal methanogens. *Proc. Natl. Acad. Sci. U.S.A.* **99**, 4644–4649 (2002).

23. D. Prakash, Y. Wu, S. J. Suh, E. C. Duin, Elucidating the process of activation of methyl-coenzyme M reductase. *J. Bacteriol.* **196**, 2491–2498 (2014).

24. F. Ramírez-Amador, S. Paul, A. Kumar, C. Lorent, S. Keller, S. Bohn, T. Nguyen, S. Lometto, D. Vlegels, J. Kahnt, D. Deobald, F. Abendroth, O. Vázquez, G. Hochberg, S. Scheller, S. T. Stripp, J. M. Schuller, Structure of the ATP-driven methyl-coenzyme M reductase activation complex. *Nature* **642**, 814–821 (2025).

25. R. K. Thauer, A. K. Kaster, M. Goenrich, M. Schick, T. Hiromoto, S. Shima, Hydrogenases from methanogenic archaea, nickel, a novel cofactor, and $H_2$ storage. *Annu. Rev. Biochem.* **79**, 507–536 (2010).

26. T. J. Lie, K. C. Costa, B. Lupa, S. Korpole, W. B. Whitman, J. A. Leigh, Essential anaplerotic role for the energy-converting hydrogenase Eha in hydrogenotrophic methanogenesis. *Proc. Natl. Acad. Sci. U.S.A.* **109**, 15473–15478 (2012).

27. R. Conrad, C. Erkel, W. Liesack, Rice Cluster I methanogens, an important group of *Archaea* producing greenhouse gas in soil. *Curr. Opin. Biotech.* **17**, 262–267 (2006).

28. S. Sakai, H. Imachi, Y. Sekiguchi, A. Ohashi, H. Harada, Y. Kamagata, Isolation of key methanogens for global methane emission from rice paddy fields: A novel isolate affiliated with the clone cluster rice cluster I. *Appl. Environ. Microbiol.* **73**, 4326–4331 (2007).

29. H. M. Dietrich, R. D. Righetto, A. Kumar, W. Wietrzynski, R. Trischler, S. K. Schuller, J. Wagner, F. M. Schwarz, B. D. Engel, V. Müller, J. M. Schuller, Membrane-anchored HDCR nanowires drive hydrogen-powered $CO_2$ fixation. *Nature* **607**, 823–830 (2022).

30. P. López-García, Y. Zivanovic, P. Deschamps, D. Moreira, Bacterial gene import and mesophilic adaptation in archaea. *Nat. Rev. Microbiol.* **13**, 447–456 (2015).

31. T. A. Williams, G. J. Szöllosi, A. Spang, P. G. Foster, S. E. Heaps, B. Boussau, T. J. G. Ettema, T. M. Embley, Integrative modeling of gene and genome evolution roots the archaeal tree of life. *Proc. Natl. Acad. Sci. U.S.A.* **114**, E4602–E4611 (2017).

32. T. Shibuya, M. Yoshizaki, M. Sato, K. Shimizu, K. Nakamura, S. Omori, K. Suzuki, K. Takai, H. Tsunakawa, S. Maruyama, Hydrogen-rich hydrothermal environments in the

Hadean ocean inferred from serpentinization of komatiites at 300 °C and 500 bar. *Prog. Earth Planet. Sci.* **2**, 46 (2015).

33. E. R. R. Moody, S. Álvarez-Carretero, T. A. Mahendrarajah, J. W. Clark, H. C. Betts, N. Dombrowski, L. L. Szánthó, R. A. Boyle, S. Daines, X. Chen, N. Lane, Z. H. Yang, G. A. Shields, G. J. Szöllosi, A. Spang, D. Pisani, T. A. Williams, T. M. Lenton, P. C. J. Donoghue, The nature of the last universal common ancestor and its impact on the early Earth system. *Nat. Ecol. Evol.* **8**, 1654–1666 (2024).

34. J. Ellermann, R. Hedderich, R. Bocher, R. K. Thauer, The final step in methane formation. Investigations with highly purified methyl-CoM reductase (component C) from *Methanobacterium thermoautotrophicum* (strain Marburg). *Eur. J. Biochem.* **172**, 669–677 (1988).

35. P. Schönheit, J. Moll, R. K. Thauer, Growth parameters ($K_s$, $\mu_{max}$, $Y_s$) of *Methanobacterium thermoautotrophicum*. *Arch. Microbiol.* **127**, 59–65 (1980).

36. J. Bhandari, D. Kompaniiets, A. K. Singh, C. Bator, J. Porta, B. Liu, Efficient strategies and troubleshooting for single particle cryoEM data collection using EPU. *BMC Methods* **2**, 3 (2025).

37. H. Guo, E. Franken, Y. Deng, S. Benlekbir, G. Singla Lezcano, B. Janssen, L. Yu, Z. A. Ripstein, Y. Z. Tan, J. L. Rubinstein, Electron-event representation data enable efficient cryoEM file storage with full preservation of spatial and temporal resolution. *IUCrJ* **7**, 860–869 (2020).

38. D. Kimanius, L. Dong, G. Sharov, T. Nakane, S. H. W. Scheres, New tools for automated cryo-EM single-particle analysis in RELION-4.0. *Biochem. J.* **478**, 4169–4185 (2021).

39. S. Q. Zheng, E. Palovcak, J. P. Armache, K. A. Verba, Y. Cheng, D. A. Agard, MotionCor2: Anisotropic correction of beam-induced motion for improved cryo-electron microscopy. *Nat. Methods* **14**, 331–332 (2017).

40. A. Rohou, N. Grigorieff, CTFFIND4: Fast and accurate defocus estimation from electron micrographs. *J. Struct. Biol.* **192**, 216–221 (2015).

41. A. Punjani, J. L. Rubinstein, D. J. Fleet, M. A. Brubaker, cryoSPARC: Algorithms for rapid unsupervised cryo-EM structure determination. *Nat. Methods* **14**, 290–296 (2017).

42. T. Bepler, A. Morin, M. Rapp, J. Brasch, L. Shapiro, A. J. Noble, B. Berger, Positive-unlabeled convolutional neural networks for particle picking in cryo-electron micrographs. *Nat. Methods* **16**, 1153–1160 (2019).

43. D. Kimanius, K. Jamali, M. E. Wilkinson, S. Lövestam, V. Velazhahan, T. Nakane, S. H. W. Scheres, Data-driven regularization lowers the size barrier of cryo-EM structure determination. *Nat. Methods* **21**, 1216–1221 (2024).

44. K. Jamali, L. Käll, R. Zhang, A. Brown, D. Kimanius, S. H. W. Scheres, Automated model building and protein identification in cryo-EM maps. *Nature* **628**, 450–457 (2024).

45. P. Emsley, K. Cowtan, Coot: Model-building tools for molecular graphics. *Acta Crystallogr. D Biol.* **60**, 2126–2132 (2004).

46. E. F. Pettersen, T. D. Goddard, C. C. Huang, E. C. Meng, G. S. Couch, T. I. Croll, J. H. Morris, T. E. Ferrin, UCSF ChimeraX: Structure visualization for researchers, educators, and developers. *Protein Sci.* **30**, 70–82 (2021).

47. P. V. Afonine, B. K. Poon, R. J. Read, O. V. Sobolev, T. C. Terwilliger, A. Urzhumtsev, P. D. Adams, Real-space refinement in PHENIX for cryo-EM and crystallography. *Acta Crystallogr. D Struct. Biol.* **74**, 531–544 (2018).

48. C. J. Williams, J. J. Headd, N. W. Moriarty, M. G. Prisant, L. L. Videau, L. N. Deis, V. Verma, D. A. Keedy, B. J. Hintze, V. B. Chen, S. Jain, S. M. Lewis, W. B. Arendall III, J. Snoeyink, P. D. Adams, S. C. Lovell, J. S. Richardson, D. C. Richardson, MolProbity: More and better reference data for improved all-atom structure validation. *Protein Sci.* **27**, 293–315 (2018).

49. G. Pintilie, K. Zhang, Z. Su, S. Li, M. F. Schmid, W. Chiu, Measurement of atom resolvability in cryo-EM maps with Q-scores. *Nat. Methods* **17**, 328–334 (2020).

50. J. Abramson, J. Adler, J. Dunger, R. Evans, T. Green, A. Pritzel, O. Ronneberger, L. Willmore, A. J. Ballard, J. Bambrick, S. W. Bodenstein, D. A. Evans, C.-C. Hung, M. O'Neill, D. Reiman, K. Tunyasuvunakool, Z. Wu, A. Žemgulytė, E. Arvaniti, C. Beattie, O. Bertolli, A. Bridgland, A. Cherepanov, M. Congreve, A. I. Cowen-Rivers, A. Cowie, M. Figurnov, F. B. Fuchs, H. Gladman, R. Jain, Y. A. Khan, C. M. R. Low, K. Perlin, A. Potapenko, P. Savy, S. Singh, A. Stecula, A. Thillaisundaram, C. Tong, S. Yakneen, E. D. Zhong, M. Zielinski, A. Žídek, V. Bapst, P. Kohli, M. Jaderberg, D. Hassabis, J. M. Jumper, Accurate structure prediction of biomolecular interactions with AlphaFold 3. *Nature* **630**, 493–500 (2024).

51. The UniProt Consortium, UniProt: The universal protein knowledgebase. *Nucleic Acids Res.* **45**, D158–D169 (2017).

52. X. Robert, P. Gouet, Deciphering key features in protein structures with the new ENDscript server. *Nucleic Acids Res.* **42**, W320–W324 (2014).

53. G. E. Crooks, G. Hon, J. M. Chandonia, S. E. Brenner, WebLogo: A sequence logo generator. *Genome Res.* **14**, 1188–1190 (2004).

54. E. Setzke, R. Hedderich, S. Heiden, R. K. Thauer, $H_2$: Heterodisulfide oxidoreductase complex from *Methanobacterium thermoautotrophicum*. Composition and properties. *Eur. J. Biochem.* **220**, 139–148 (1994).

55. C. Afting, A. Hochheimer, R. K. Thauer, Function of $H_2$-forming methylenetetrahydromethanopterin dehydrogenase from *Methanobacterium thermoautotrophicum* in coenzyme $F_{420}$ reduction with $H_2$. *Arch. Microbiol.* **169**, 206–210 (1998).

56. C. B. Messner, V. Demichev, N. Bloomfield, J. S. L. Yu, M. White, M. Kreidl, A. S. Egger, A. Freiwald, G. Ivosev, F. Wasim, A. Zelezniak, L. Jurgens, N. Suttorp, L. E. Sander, F. Kurth,

K. S. Lilley, M. Mülleder, S. Tate, M. Ralser, Ultra-fast proteomics with Scanning SWATH. *Nat. Biotechnol.* **39**, 846–854 (2021).

57. B. Schwanhäusser, D. Busse, N. Li, G. Dittmar, J. Schuchhardt, J. Wolf, W. Chen, M. Selbach, Global quantification of mammalian gene expression control. *Nature* **473**, 337–342 (2011).

58. P. A. Chaumeil, A. J. Mussig, P. Hugenholtz, D. H. Parks, GTDB-Tk: A toolkit to classify genomes with the genome taxonomy database. *Bioinformatics* **36**, 1925–1927 (2020).

59. C. Rinke, M. Chuvochina, A. J. Mussig, P. A. Chaumeil, A. A. Davín, D. W. Waite, W. B. Whitman, D. H. Parks, P. Hugenholtz, A standardized archaeal taxonomy for the Genome Taxonomy Database. *Nat. Microbiol.* **6**, 946–959 (2021).

60. S. Kalyaanamoorthy, B. Q. Minh, T. K. F. Wong, A. von Haeseler, L. S. Jermiin, ModelFinder: Fast model selection for accurate phylogenetic estimates. *Nat. Methods* **14**, 587–589 (2017).

61. B. Q. Minh, H. A. Schmidt, O. Chernomor, D. Schrempf, M. D. Woodhams, A. von Haeseler, R. Lanfear, IQ-TREE 2: New models and efficient methods for phylogenetic inference in the genomic era. *Mol. Biol. Evol.* **37**, 1530–1534 (2020).

62. D. T. Hoang, O. Chernomor, A. von Haeseler, B. Q. Minh, L. S. Vinh, UFBoot2: Improving the ultrafast bootstrap approximation. *Mol. Biol. Evol.* **35**, 518–522 (2018).

63. K. P. Schliep, phangorn: Phylogenetic analysis in R. *Bioinformatics* **27**, 592–593 (2011).

64. D. L. Swofford, W. P. Maddison, Reconstructing ancestral character states under Wagner parsimony. *Math. Biosci.* **87**, 199–229 (1987).

65. G. J. Mander, A. J. Pierik, H. Huber, R. Hedderich, Two distinct heterodisulfide reductase-like enzymes in the sulfate-reducing archaeon *Archaeoglobus profundus*. *Eur. J. Biochem.* **271**, 1106–1116 (2004).

66. J. A. Vorholt, M. Vaupel, R. K. Thauer, A selenium-dependent and a selenium-independent formylmethanofuran dehydrogenase and their transcriptional regulation in the hyperthermophilic *Methanopyrus kandleri*. *Mol. Microbiol.* **23**, 1033–1042 (1997).

67. R. A. Schmitz, S. P. J. Albracht, R. K. Thauer, A molybdenum and a tungsten isoenzyme of formylmethanofuran dehydrogenase in the thermophilic archaeon *Methanobacterium wolfei*. *Eur. J. Biochem.* **209**, 1013–1018 (1992).

68. P. A. Bertram, R. A. Schmitz, D. Linder, R. K. Thauer, Tungstate can substitute for molybdate in sustaining growth of *Methanobacterium thermoautotrophicum*. *Arch. Microbiol.* **161**, 220–228 (1994).

69. S. Shima, D. A. Hérault, A. Berkessel, R. K. Thauer, Activation and thermostabilization effects of cyclic 2,3-diphosphoglycerate on enzymes from the hyperthermophilic *Methanopyrus kandleri*. *Arch. Microbiol.* **170**, 469–472 (1998).

70. S. Shima, C. Tziatzios, D. Schubert, H. Fukada, K. Takahashi, U. Ermler, R. K. Thauer, Lyotropic-salt-induced changes in monomer/dimer/tetramer association equilibrium of formyltransferase from the hyperthermophilic *Methanopyrus kandleri* in relation to the activity and thermostability of the enzyme. *Eur. J. Biochem.* **258**, 85–92 (1998).

71. D. Liebschner, P. V. Afonine, M. L. Baker, G. Bunkoczi, V. B. Chen, T. I. Croll, B. Hintze, L. W. Hung, S. Jain, A. J. McCoy, N. W. Moriarty, R. D. Oeffner, B. K. Poon, M. G. Prisant, R. J. Read, J. S. Richardson, D. C. Richardson, M. D. Sammito, O. V. Sobolev, D. H. Stockwell, T. C. Terwilliger, A. G. Urzhumtsev, L. L. Videau, C. J. Williams, P. D. Adams, Macromolecular structure determination using X-rays, neutrons and electrons: Recent developments in Phenix. *Acta Crystallogr. D Struct. Biol.* **75**, 861–877 (2019).
